# Anti-Inflammatory Lindolin Alkaloids Repress the Transcription of the Microsomal Prostaglandin E_2_ Synthase‑1 Gene in Macrophages

**DOI:** 10.1021/acs.jnatprod.5c01488

**Published:** 2026-02-11

**Authors:** Paul M. Jordan, Johannes Rassbach, Melina Gräfe, Lukas K. Peltner, Karsten Willing, Lukas Zenkel, Kerstin Günther, Robin Sonnabend, Lars Regestein, Oliver Werz, Markus Gressler

**Affiliations:** † Pharmaceutical/Medicinal Chemistry, Institute of Pharmacy, 9378 Friedrich Schiller University Jena, Philosophenweg 14, 07743 Jena, Germany; ‡ Jena Center for Soft Matter (JCSM), Friedrich Schiller University Jena, Philosophenweg 7, 07743 Jena, Germany; § Pharmaceutical Microbiology, Institute of Pharmacy, Friedrich Schiller University Jena, Winzerlaer Strasse 2, 07745 Jena, Germany; ∥ Pharmaceutical Microbiology, Leibniz Institute for Natural Product Research and Infection Biology, 28406 Hans Knöll Institute, Beutenbergstrasse 11a, 07745 Jena, Germany; ⊥ Bio Pilot Plant, Leibniz Institute for Natural Product Research and Infection, Biology, Hans Knöll Institute, Beutenbergstrasse 11a, 07745 Jena, Germany

## Abstract

The known indole-3-acetyl anthranilate lindolin A from the early-diverging fungus *Linderina pennispora*, along with three novel semisynthetic congeners, were identified as selective inhibitors of prostaglandin E_2_ (PGE_2_) formation. Lindolins specifically suppress the expression of the microsomal prostaglandin E_2_ synthase-1 (mPGES-1) gene in M1-polarized human macrophages, resulting in markedly reduced mPGES-1 protein levels and a consequent decrease in PGE_2_ production, while leaving other lipid mediators largely unaffected. The biosynthesis of lindolins involves the promiscuous *N*-acyltransferase LinB, which accepts a variety of substituted anthranilic acids as acceptor substrates, thereby enabling the *in vitro* generation of an analog library. Structure–activity relationship studies revealed that four lindolin analogs with enhanced anti-inflammatory activity possess an *ortho*-substituted carboxyl group, a structural feature shared with the clinically used antiallergic drug tranilast. Due to their high selectivity toward the *mPGES-1* gene expression, lindolins represent promising lead structures for the development of selective mPGES-1 inhibitors with a novel mode of action. Moreover, these findings highlight early-diverging fungi as underestimated source of compounds with pharmaceutical potential.

## Graphical abstract

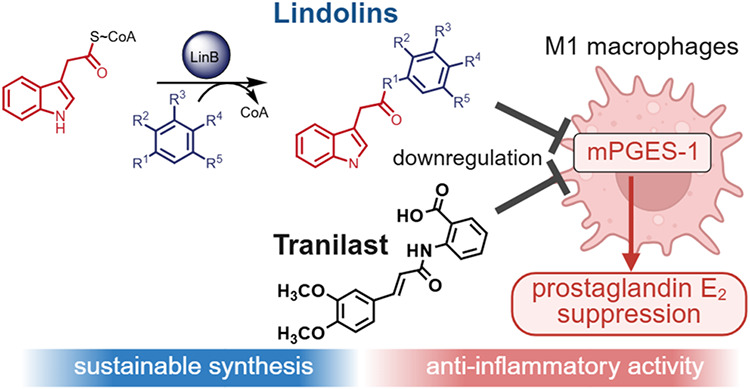

Prostaglandins (PG) are bioactive lipid mediators (LM) involved in numerous physiological processes, including gastrointestinal protection, blood pressure regulation, ovulation, and natriuresis.^1^ They also play a central role in pathological conditions such as inflammation, where prostaglandin E_2_ (PGE_2_) is the key mediator in acute inflammatory responses, pain, and fever.^2^ Upon cell stimulation, phospholipase A_2_ releases arachidonic acid (AA) from membrane phospholipids (Figure 1A). AA is subsequently converted by the cyclooxygenase (COX) isoforms 1 and 2 to the unstable intermediate PGH_2_, which is then isomerized to PGE_2_ by one of three prostaglandin E synthases: cytosolic cPGES, microsomal mPGES-1, and mPGES-2.^3^ In contrast to the constitutively expressed *cPGES* and *mPGES-2*, the expression of *mPGES-1* is strongly induced during inflammation and hence, functionally coupled with COX-2.^4,5^

**Figure 1:**
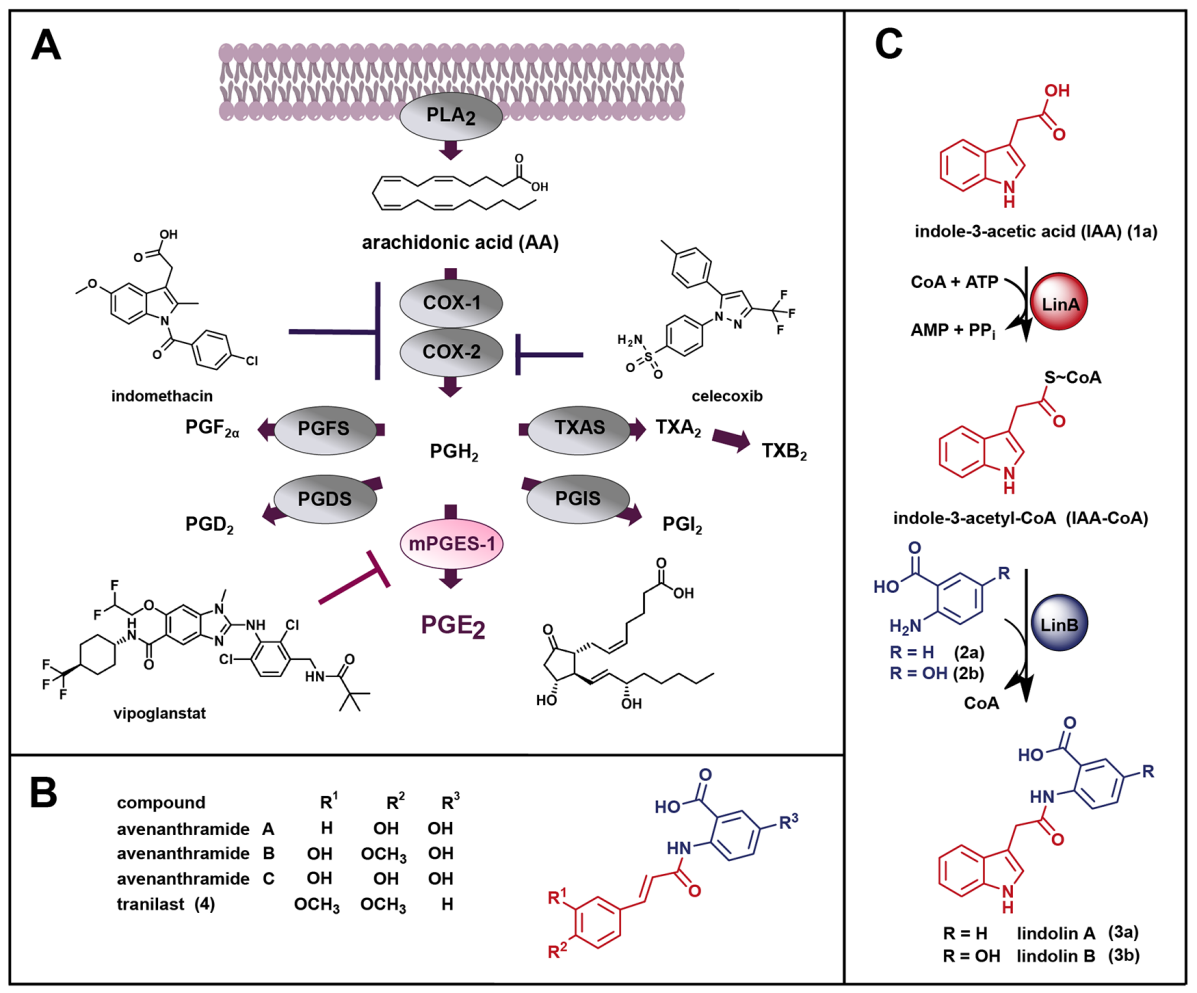
(A) Arachidonic acid cascade and biosynthesis of prostanoids with pharmaceutical relevant targets for anti-inflammatory drugs. COX, cyclooxygenase; PGDS, prostaglandin D_2_ synthase; PGD_2_, prostaglandin D_2_; cPGES/mPGES, cytosolic/microsomal prostaglandin E_2_ synthase; PGE_2_, prostaglandin E_2_; PGFS, prostaglandin F_2_ synthase; PGF_2α_, prostaglandin F_2α_; PGH_2_, prostaglandin H_2_; PGIS, prostacyclin synthase; PGI_2_, prostacyclin; PLA_2_, phospholipase A_2_; TXAS, thromboxane A_2_ synthase; TXA_2_/TXB_2_, thromboxane A_2_/B_2_. (B) Chemical structures of avenanthramide A–C and tranilast (**4**). (C) Biosynthetic route for lindolin A (**3a**) and lindolin B (**3b**) starting from indole-3-acetic acid (IAA, **1a**) and (5-hydroxy)­anthranilate (**2a**, **2b**).

Conventional nonsteroidal anti-inflammatory drugs (NSAIDs) such as indomethacin reduce PGE_2_ biosynthesis by inhibiting COX enzymes, but are associated with adverse gastrointestinal side effects due to impaired PGE_2_ formation in the digestive tract.^6,7^ Although selective COX-2 inhibitors such as celecoxib were developed to address these limitations, they instead cause cardiovascular risks. Therefore, mPGES-1 has emerged as a promising PGE_2_-specific drug target without interfering with constitutive PGE_2_ formation and other prostanoid pathways.^8,9^

Within recent years, synthetic mPGES-1 inhibitors such as vipoglanstat have emerged, some of which have advanced into (pre)­clinical trials.^10–13^ Despite this progress, natural product- based mPGES-1 inhibitors remain largely underdeveloped.^14,15^

Promising examples, however, are the plant-derived avenanthramides (Figure 1B) showing multiple anti-inflammatory effects including reduced COX-2 expression and decreased PGE_2_ levels in skeletal muscle cells.^16,17^ These diphenolic compounds served as structural template for the development of the antiallergic drug tranilast (Figure 1B) which has been introduced into clinical use in East Asia since 1982.^18,19^ Tranilast (**4**) is known for its pleiotropic anti-inflammatory effects, including the reduction of PGE_2_ and PGD_2_ levels in monocytes, macrophages^20,21^ and mast cells;^22^ the downregulation of *COX-2* expression in fibroblasts;^23^ the suppression of fibrinolytic pathways by targeting TGF-β in liver,^24,25^ kidney,^26,27^ lung^28,29^ and heart cells;^30,31^ and the inhibition of collagen production in hypertrophic scars.^32^ Accordingly, tranilast has a broad range of medical applications, but it acts nonselectively.

Structurally related indole alkaloids, the lindolins (Figure 1C), were recently isolated from *Linderina pennispora*.^33^ *L. pennispora* is a representative of the so-called early-diverging fungi (EDF) that have emerged as a novel, yet largely untapped source of bioactive compounds.^33–37^ EDF interact with a variety of organisms, including bacterial endosymbionts,^34,38–40^ plants (*e.g.*, as arbuscular mycorrhizae)^41^ or other fungi either intraspecifically during sexual reproduction^42^ or interspecifically *via* mycoparasitism.^43^ Genetic analyses of several EDF genera (*e.g.*, *Mortierella*, *Basidiobolus*) suggest that genes encoding nonribosomal peptide synthetases (NRPS) were likely acquired *via* horizontal gene transfer from bacterial endosymbionts.^44–46^

In parallel, EDF evolved their own enzymatic machineries for natural product biosynthesis.^33^ Lindolins from *L. pennispora* represent a prominent example for an NRPS-independent dipeptide formation in fungi: Lindolins are derived from indole-3-acetic acid (IAA, **1a**) and anthranilate (**2a**) and are biosynthesized by a two-step enzymatic pathway involving an IAA-coenzyme A (IAA-CoA) ligase (LinA) and a unique IAA-CoA:anthranilate *N*-indole-3-acetyltransferase (LinB) (Figure 1C).^33^ The *linB* gene is present in genomes of >170 species within the EDF order Kickxellales, but is absent from all other sequenced genomes across the kingdoms of life, indicating an independent evolution of secondary metabolism in EDF.^33^ The two main products, lindolins A and B (**3a, 3b**), exhibit moderate activity against plant-pathogenic oomycetes.^33^ However, their anti-inflammatory potential has not yet been investigated.

Motivated by their structural resemblance to tranilast (**4**), we investigated the anti-inflammatory properties of lindolins and a set of semisynthetic analogs. Using a sustainable enzyme-catalyzed route, a library of 11 lindolin derivatives was generated, of which four compounds showed selective inhibitory activity on PGE_2_ production in pro-inflammatory M1 monocyte-derived macrophages. Structure–activity relationships pointed to an *ortho*-substituted carboxyl group in lindolins as the critical pharmacophore. Mechanistic studies revealed a specific downregulation of *mPGES-1* expression at both the mRNA and protein levels, indicating that lindolins represent a novel class of PGE_2_-selective, natural product-based anti-inflammatory agents.

## Results and Discussion

### Substrate Promiscuity of LinB Enables Biocatalytic Access to a Diverse Lindolin Library

In order to sustainably produce a lindolin library by biocatalysis, both biosynthetic enzymes—the ligase LinA and the transferase LinB—were heterologously produced as His_6_-tagged fusion proteins in lab- and pilot-scale fermentations of *Escherichia coli* (20 and 5 L, respectively). Metal affinity chromatography yielded 3.2 g of pure LinA, and 0.8 g of LinB (Figures S1–S3 and Table S1).

The LinA/LinB enzyme system was employed *in vitro* to explore its substrate flexibility using a panel of donor and acceptor substrates. LinA proved highly specific, accepting only its natural donor substrate indole-3-acetic acid (**1a**) among the 11 tested carboxylic acids (**1a–1k**) (Figure 2A, 2B).

**Figure 2:**
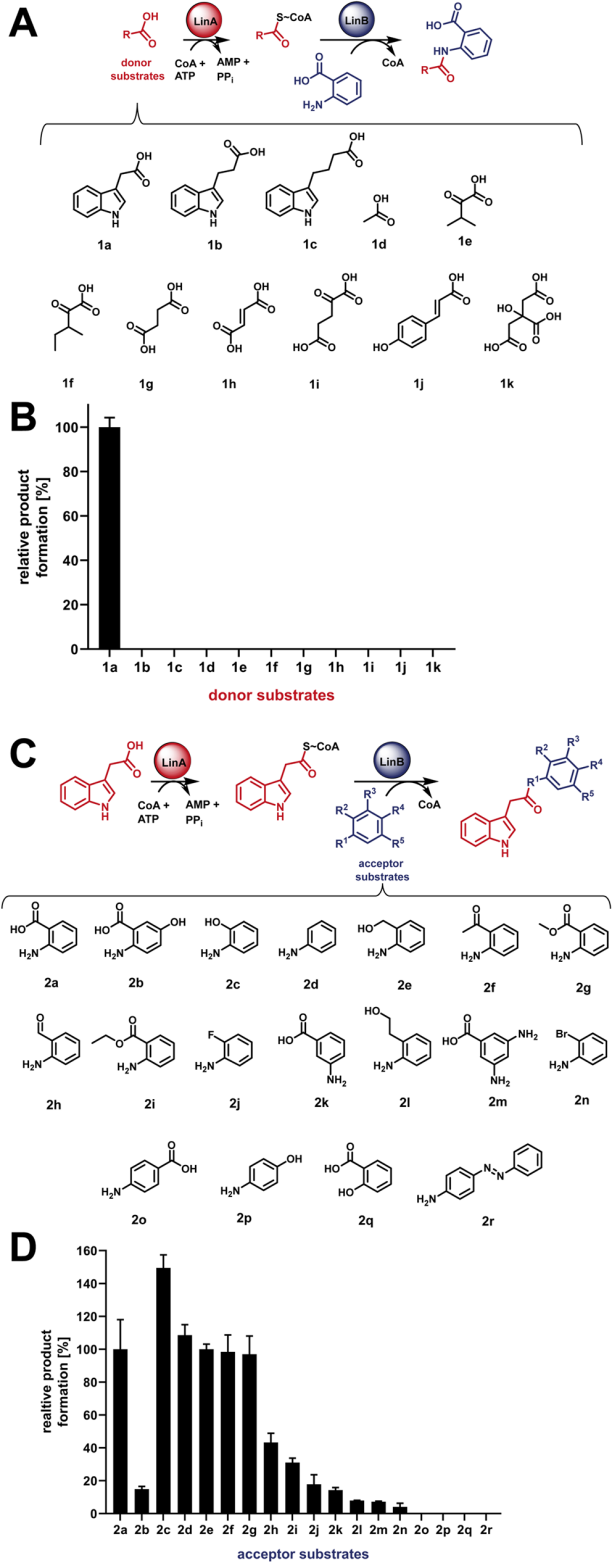
Promiscuity of the LinA/LinB enzyme system. Various donor (A, B) and acceptor substrates (C, D) were tested. (B) The LinA/LinB enzyme system is strictly specific for the donor substrate indole-3-acetic acid (**1a**). (D) However, it shows plasticity on the acceptor substrates (**2a**–**2n**). Note that **1f** was tested as a racemic mixture.

In contrast, LinB exhibited eminent promiscuity toward anthranilate derivatives as acceptor substrates. In addition to the two natural acceptor substrates anthranilate (**2a**) and 5-hydroxyanthranilate (**2b**) – yielding lindolin A (**3a**) and B (**3b**) respectively—12 out of the 16 additionally tested acceptor substrates (**2c**–**2r**) led to the formation of novel lindolin derivatives in varying amounts (Figure 2C,2D). LinB prefers *ortho*-substituted anthranilate derivatives, while *meta*-substituted derivatives were turned over only marginally (*e.g.*, **2k**) and *para*-substituted analogs (**2o**) completely abolished product formation. Notably, conversion of anthranilic acid (**2a**), but not its hydroxy acid counterpart salicylic acid (**2q**), suggests that LinB catalyzes amidation rather than esterification, indicating a mechanistic preference.

Steric constraints were also evident: Anthranilate analogs with shorter (one or two atoms) side chains (**2c**, **2e**, **2g**) were more efficiently accepted than bulkier analogs with three- to four-membered substituents (**2i**, **2l**). Halogen-substituted substrates were tolerated in a size-dependent manner (H ≈ OH > *F* > Br; *e.g.*, **2d, 2c, 2j, 2n**). These findings not only highlight the catalytic flexibility of LinB but also provide a rationale for the natural occurrence of lindolin B (**3b**) in *L. pennispora*.^33^

From 50 to 200 mL upscaled LinA/LinB enzymatic reactions, the two natural lindolins (**3a** and **3b**), along with the most abundant synthetic lindolins C–K (**3c**–**3k**), were isolated by HPLC (Table S2). Yields of compounds **3c**–**3k** ranged from 1.8 to 6.8 mg per 100 mL assay (Tables S3–S4). The chemical structures were confirmed through comprehensive NMR analyses (Figures 3, S4–S93, Tables S3, S5–S13).

**Figure 3:**
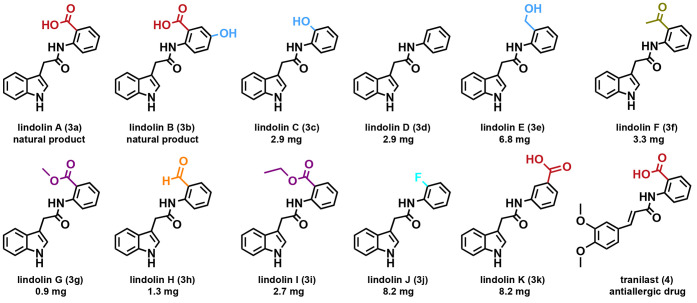
Structures and total yields of the lindolin derivatives **3a**–**3k** produced *in vitro*, along with the anti-inflammatory drug tranilast (**4**). Total yields are given as the mass of isolated compound per reaction volume (**3c, 3d, 3f, 3g**: 50 mL; **3e, 3h, 3i**: 100 mL; **3j, 3k**: 200 mL).

Current synthetic approaches toward lindolin-like compounds either rely on time-consuming two-step routes involving highly reactive intermediates such as indole-3-acetyl chloride^47^ or CDI-mediated activation of indole-3-acetic acid.^48^ Alternatively, one-pot reactions with high yields have been developed, but these mechanistically require hazardous solvents such as *N,N*-dimethylformamide and dichloromethane.^49^ In contrast, the efficient single-step turnover of indole donor and anthranilate acceptor substrates by LinA/LinB in an aqueous system constitutes a more sustainable route, at least for the production of compounds **3a–3k**.

Previous bioactivity assays against the plant pathogenic oomycete *Phytophthora megasperma* revealed growth inhibition by the natural **3a** at a MIC of 100 μM.^33^ We therefore tested also **3b** and the semisynthetic analogs. In comparison to **3a**, the congeners **3b, 3d** and **3h** exhibited approximately 2- to 4-fold lower activity (Figure S94). None of the remaining congeners displayed antioomycetic activity, highlighting the specific potency of native lindolins against *Phytophthora* species.

### Lindolin Congeners Exhibit Anti-Inflammatory Activity Primarily by Reducing PGE_2_ Formation in Human M1-like Macrophages

Due to their structural similarity to tranilast (**4**) (Figure 3) potential impact of lindolins on cytokine production and PG biosynthesis was assumed. To investigate this hypothesis, we employed pro-inflammatory monocyte-derived M1 macrophages as suitable model systems to study effects on cytokine release and PG biosynthetic pathways.

We first examined whether lindolin derivatives (**3a**–**3k**) or tranilast (**4**) affect the LPS/IFNγ-induced polarization of naïve (M0) macrophages toward the pro-inflammatory M1 phenotype (Figure 4A) and assessed the potential interference with the release of pro-inflammatory cytokines, including IL-1β, TNF-α and IL-6 (Figure S95). As expected,^50^ LPS/IFNγ-priming resulted in only marginal IL-1β release, while TNF-α and IL-6 were robustly induced in M1 macrophages (Figure S95). As **4** has been reported to inhibit the inflammasome in murine macrophages,^51^ **3a**–**3k** and **4** were further evaluated for potential effects on cytokine release (Figure S95). However, **4** did not suppress TNF-α or IL-6 release, which is consistent with previous findings.^51^ Likewise, **3a**–**3k** did not impact TNF-α release and only slightly, though not significantly, reduced IL-6 levels. Importantly, none of the tested compounds, including **4** and lindolins (except **3k**), showed cytotoxicity after 24 h of treatment under the testing conditions (Figure 4B). Hence, lindolins neither induce cytotoxicity nor modulate pro-inflammatory cytokine production in M1 macrophages.

**Figure 4:**
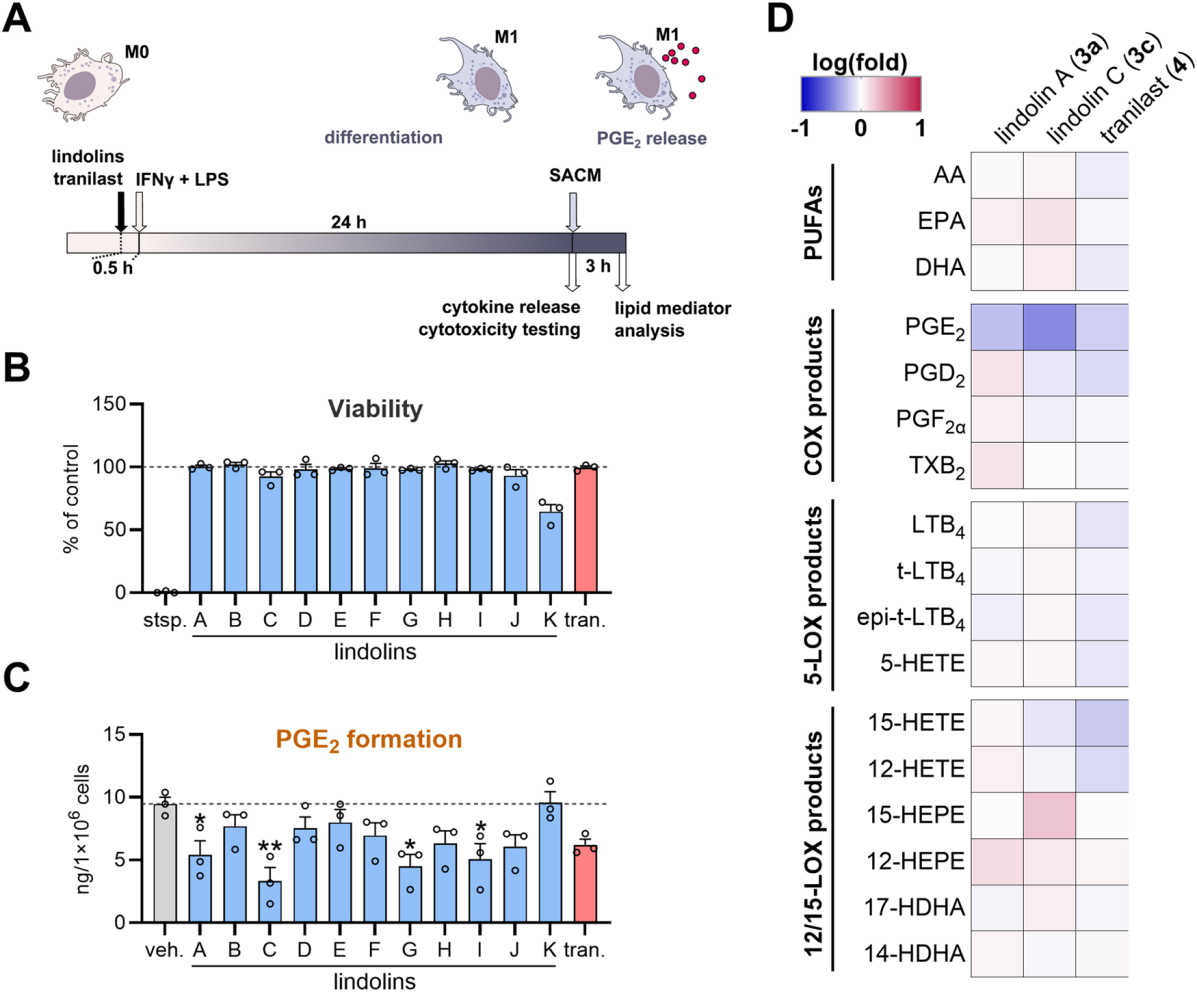
Prostaglandin E_2_ formation is efficiently suppressed by lindolins in M1 macrophages. (A) Experimental scheme for treatment of M1 macrophages. Human M0 macrophages (1 × 10^6^ cells) were pretreated with 50 μM lindolins, 50 μM tranilast or 0.1% DMSO (as vehicle control) for 30 min prior to polarization with LPS/IFNγ for additional 24 h at 37 °C. Cells were then used for cytokine detection and cytotoxicity assays or stimulated with 1% *Staphylococcus aureus* conditioned medium (SACM) for 180 min, after which lipid mediators released into the medium were analyzed by UHPLC-MS/MS. (B) Cytotoxicity is shown as metabolic viability (MTT assay) of M1 macrophages after incubation for 24 h with 50 μM tranilast (tran.) or lindolins; staurosporine (stsp., 1 μM) was used as positive control. Data are presented as mean ± SEM in bar charts with individual data points (circles) (*n* = 3). (C) PGE_2_ amounts formed by M1 macrophages are shown in ng per 10^6^ cells. Bars represent means + SEM with single values (circles) (*n* = 3). Statistical analysis was performed using one-way ANOVA with Dunnett‘s multiple comparisons test. *p < 0.05, **p < 0.01. (D) Release of lipid mediators after long-term treatment with lindolin A (**3a**), lindolin C (**3c**), or tranilast (**4**). Data are presented as a heatmap. Lipid mediator production is given as (log)­fold change relative to the vehicle-treated controls.

We next evaluated the impact of lindolin derivatives (**3a**–**3k**) or tranilast (**4**) on PG production (Figure 4A). PG production in the M1 macrophages was induced by the addition of *Staphylococcus aureus* conditioned medium (SACM) as previously described.^52^

Lindolins A, C, G, and I (**3a, 3c, 3g, 3i**) significantly decreased PGE_2_ formation with effects comparable to those of **4** (Figure 4C). Among these, compounds **3c** and **3a** exerted the strongest activity, reducing PGE_2_ to 35% and 50% relative to vehicle control, compared to only 57% inhibition by **4**. The other lindolin congeners showed minor, nonsignificant effects.

To assess the selectivity of PGE_2_ suppression, additional LMs were quantified following treatment with **3a, 3c** and **4** (Figure 4D). Consistent with earlier studies,^21,22^ **4** also decreased PGD_2_ levels, which was not evident for **3a** or **3c**. The formation of additional COX products such as PGF_2α_ and TXB_2_ remained unaffected by either compound, suggesting that lindolins are selective inhibitors of the PGE_**2**_ biosynthetic pathway. Moreover, formation of leukotrienes derived from 5-lipoxygenase (5-LOX), and also 12-LOX and 15-LOX activity were not altered by lindolin treatment. In contrast, **4** strongly repressed also the production of 12- and 15-hydroxyeicosatetraenoic acid (12-HETE, 15-HETE), indicating a broader effect, *i.e.*, inhibition of 12/15-LOX. The release of polyunsaturated fatty acids (PUFAs) such as arachidonic acid (AA), eicosapentaenoic acid (EPA), or docosahexaenoic acid (DHA) was slightly—but not significantly—increased under long-term treatment with **3a** or **3c**, whereas **4** led to a modest reduction in PUFA release. Taken together, these data suggest that lindolins exert PGE_2_-suppressive activity comparable to **4**, yet appear to act with higher selectivity.

### Bioactivity of Lindolins Depends on *ortho*-Substituted Hydrogen Bond Donors

The screening of the library provided preliminary insights into the structure–activity relationships of lindolins. Several congeners bearing either a hydroxy group (**3c**), a carboxylic acid (**3a**) or its respective esters (**3g, 3j**) exhibited the most pronounced activity in reducing PGE_2_ formation in M1 macrophages (Figure 4C). Notably, these moieties are positioned *ortho* to the amide group of the anthranilate.

In contrast, lindolin K (**3k**), which features a *meta*-positioned carboxylic acid, and lindolin D (**3d**), lacking any hydrogen-bond-donating substituent, have no measurable effect on PGE_2_ formation. These observations suggest that *ortho* substitution with polar functionalities may favor bioactivity, although this feature alone is not sufficient to confer activity across the entire series, as exemplified by lindolin B (**3b**) and lindolin E (**3e**).

Across the set of active compounds, the *ortho*-positioned substituents exert a shared electron-withdrawing inductive (−I) effect. Since the clinically used drug tranilast and avenanthramides also feature a carboxylate in *ortho*-position to the amide, we propose that the *o*-anthranilate moiety constitutes the PGE_2_-suppressing pharmacophore common to all three compound classes. However, given the size of the lindolin compound set, this interpretation should be regarded as preliminary.

The presence of a hydrogen bond donor correlates with high activity in selected analogs such as **3c** (hydroxy group) and **3a** (carboxylic acid). Surprisingly, **3g** and **3i**—containing carboxylate esters—also significantly reduce PGE_2_ levels. We hypothesize that these esters may be intracellularly hydrolyzed to yield the active acid form (**3a**), consistent with a pro-drug mechanism similar to that of cephalosporin pro-drug esters.^53^ Ester cleavages of **3g**-like compounds in mammalian cells have been reported, *e.g.*, the hydrolysis of *O*-methyl anthranilate in hepatocytes of guinea pigs and rats, and the degradation of *O*-propyl anthranilate in intestinal cells of rats.^54–56^ Moreover, ester hydrolysis in macrophages is a widely exploited pharmacological strategy in the targeted delivery of active drugs^57^ and represents a plausible explanation for the observed activity; however, the intracellular metabolic turnover of the lindolin esters was not directly investigated in the present study.

### Lindolins Neither Affect COX-2 Nor mPGES-1 Activity

To further explore the mechanism underlying the selective inhibition of PGE_2_ production, we investigated whether lindolins act as direct inhibitors of COX-2 or mPGES-1. In a short-term experiment, M1 macrophages were treated with lindolin A (**3a**). As positive controls, the selective COX-2 inhibitor, celecoxib,^58^ and the mPGES-1 inhibitor vipoglanstat (BI 1029539; GS-248),^12^ that has recently entered phase II clinical trials, were used.

The polarized M1 macrophages were pretreated with either **3a**, celecoxib or vipoglanstat for 30 min, followed by stimulation with SACM for 90 min to release PGs (Figure S96). In this short-term setting, **3a** had no detectable effect on prostanoid levels (PGE_2_, PGD_2_, PGF_2α_, TXB_2_), indicating that neither COX-2 nor mPGES-1 activity was affected (Figure 5A,5B). However, direct interaction with these enzymes remains to be assessed using purified proteins in cell-free systems.

**Figure 5:**
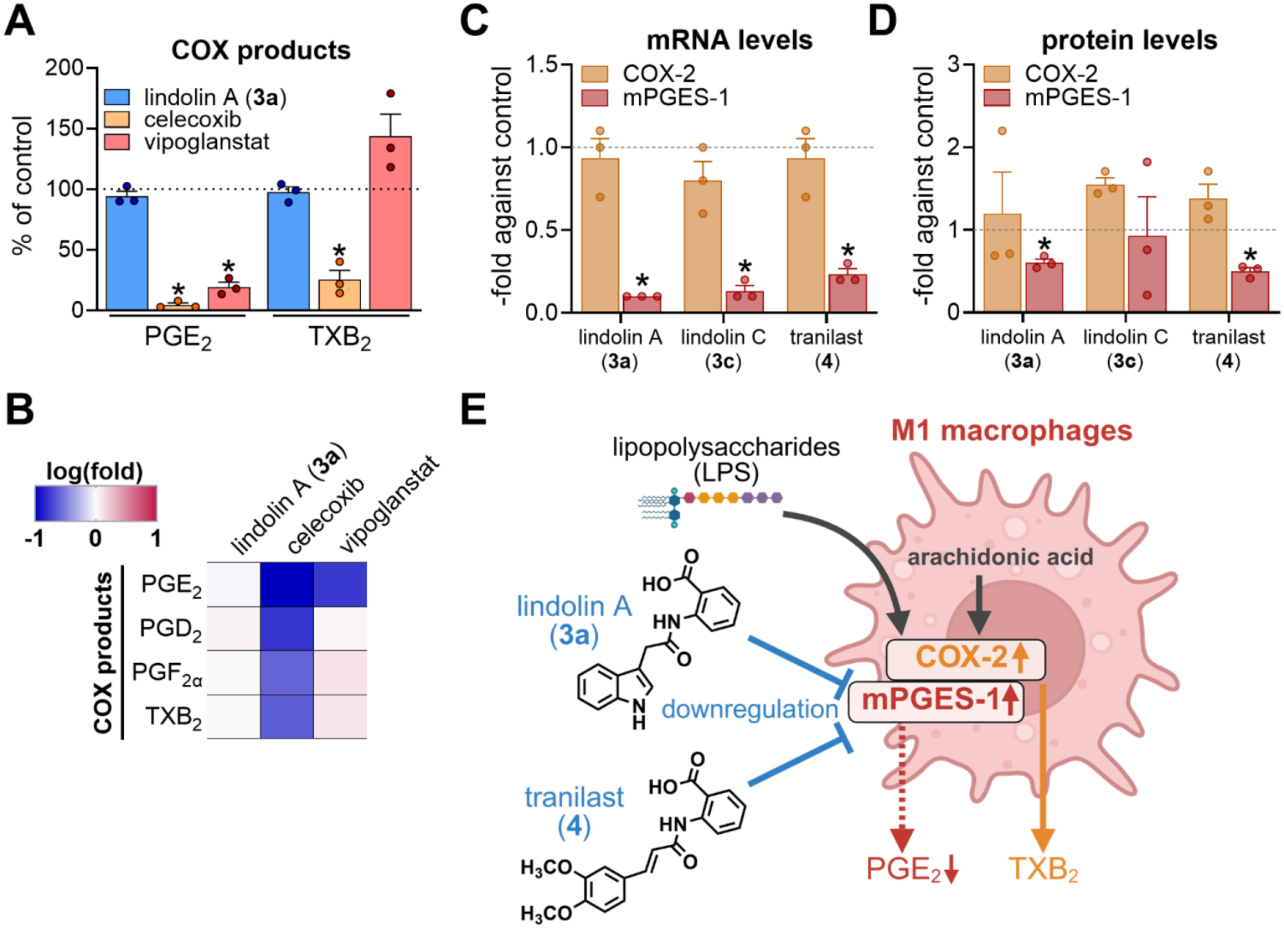
Lindolins downregulate *mPGES-1* but not *COX-2* expression. (A, B) Polarized M1 macrophages (1 × 10^6^ cells) were pretreated with 30 μM lindolin A (**3a**), 3 μM celecoxib, 30 nM vipoglanstat or 0.1% DMSO (vehicle control) for 30 min prior to stimulation with 1% SACM for another 90 min. Released LMs in cell supernatants were analyzed by UHPLC-MS/MS. (A) Quantification of COX-derived PGE_2_ and TXB_2_ levels after short-term treatment with lindolin A (**3a**), celecoxib and vipoglanstat. Data are presented relative to the vehicle control and are given as means ± SEM (*n* = 3). * *p* < 0.05: one-way ANOVA for multiple comparisons with Dunnett‘s multiple comparison. (B) COX product formation. Data is shown as a heatmap with log­(fold) of vehicle control. (C, D) M0 macrophages (1 × 10^6^ cells) were pretreated with 50 μM lindolin A (**3a**), lindolin C (**3c**), or tranilast (**4**), or 0.1% DMSO (vehicle control) for 30 min prior to polarization with LPS/IFNγ. mRNA levels and protein levels were determined after 6 and 24 h after polarization, respectively. *COX-2* and *mPGES-1* mRNA expression (C) was determined by qRT-PCR and protein levels (D) were quantified by Western Blot analysis (Figure S97). Data are presented as fold change against vehicle control and are given as means ± SEM * *p* < 0.05: one-way ANOVA with Dunnett‘s multiple comparison. (E) Scheme of the mechanistic regulation in pro-inflammatory M1 macrophages by lindolins and tranilast. Lipopolysaccharides (LPS) induce the expression of the *mPGES-1* and *COX-2* genes resulting in the production of PGE_2_ and other prostanoids such as TXB_2_. Lindolin A and tranilast downregulate the LPS-mediated induction of *mPGES-1*, but not *COX-2*, resulting in a selective suppression of PGE_2_.

In contrast and as expected, celecoxib significantly reduced the levels of all COX-derived products (Figure 5B) and redirected arachidonic acid metabolism toward the 5-LOX pathway, resulting in increased production of leukotrienes (data not shown). These results are consistent with previous findings on COX-2 inhibition in macrophages.^59^ Moreover, the specific mPGES-1 inhibitor vipoglanstat, selectively suppressed PGE_2_ formation, while other LMs (including PGs) where not or hardly affected. This finding is in line with current *in vivo* studies.^60,61^

Together, these results suggest that suppression of PGE_2_ formation by lindolins (and tranilast) during long-term treatment (24 h) involves another mechanism.

### Lindolins Downregulate *mPGES-1* Expression

Since lindolin A (**3a**) did not affect PGE_2_ production under short-term treatment (90 min) (Figure 5A), but exerted a selective and potent reduction after long-term treatment (24 h) (Figure 4D), we hypothesized a slower mechanism of interference—likely involving transcriptional regulation—rather than direct enzyme inhibition.

We therefore quantified the mRNA levels of the *COX-2* and *mPGES-1* genes after 6 h of macrophage polarization with and without compound treatment (Figure 5C). qRT-PCR analysis revealed that **3a**, **3c** and **4** had no effect on the *COX-2* transcription, but showed a pronounced repression of the *mPGES-1* gene. While **4** reduced *mPGES-1* expression by approximately 5-fold, **3a** and **3c** led to even 10-fold and 8-fold downregulation, respectively.

These results suggested that both lindolins and tranilast act through selective downregulation of *mPGES-1* transcription (Figure 5C). We also assessed the protein levels of COX-2 and mPGES-1 in M1 macrophages after 24 h of treatment with **3a**, **3c** and **4** using Western Blot analysis (Figures 5D, and S95). Consistent with our expression analysis, protein levels for COX-2 remained unchanged regardless of the treatment. In contrast, mPGES-1 levels decreased to 50% in cells treated with **3a** or **4**. However, a modest, yet not significant, reduction of mPGES-1 protein levels was also observed for **3c**.

Taking together, the suppression of PGE_2_ by lindolins (and tranilast) is mediated by the downregulation of *mPGES-1* expression and consequently impaired enzyme levels (Figure 5E). In contrast to known mPGES-1 inhibitors that directly block activity of the enzyme, our results unveiled the interference with the *mPGES-1* transcription as an alternative mode of action for suppressing PGE_2_ formation. Future research is warranted to reveal, how the downregulation is mediated and whether potential transcription factors are involved.

## Conclusions

Lindolins and the reference drug tranilast were identified as transcriptional repressors of the *mPGES-1* gene in human polarized M1 macrophages. The substrate flexibility of the lindolin biosynthetic *N*-acyltransferase LinB enables the biocatalytic synthesis of lindolin congeners *in vitro* through supplementation with various anthranilic acid derivatives, facilitating substrate-activity relationship studies. An *ortho*-substituted carboxyl group was identified as critical for bioactivity.

Lindolins mediate anti-inflammatory properties through the reduced production of PGE_2_, whereas other LMs remain unaffected. This is an unprecedented mechanism to selectively inhibit PGE_2_ production, namely *via* the transcriptional repression of the *mPGES-1* gene, and opens new avenues for anti-inflammatory drug discovery. Future studies will focus on identifying the molecular target responsible for the transcriptional downregulation of *mPGES-1* and will evaluate the applicability of this mode of action in additional human immune cell types.

Moreover, this study highlights EDF as a valuable source of pharmaceutically relevant natural products. Although EDF have only recently been recognized for their biosynthetic potential, their metabolic pathways remained largely unexplored, as they often rely on unique enzymes and lack conventional biosynthetic gene clusters that typically facilitate genome-based compound discovery in higher fungi.^33^ However, EDF achieve remarkable chemical diversity through the broad substrate tolerance of their enzymes,^35,44^ enabling biocatalytic access to structurally diverse metabolites, as demonstrated for LinB in this work. Hence, EDF not only represent a promising resource for novel bioactive natural products, but also serve as a reservoir of unique biocatalysts with potential applications in synthetic biology and drug discovery.

## Experimental Section

### General Experimental Procedures

Chemicals were obtained from Carl Roth, VWR or Merck (Germany), unless stated otherwise. Flash chromatographic fractionation was performed on a Büchi Pure C-810 system. UV spectra and routine LC-MS experiments of lindolins were recorded on an Agilent 1290 Infinity II UHPLC coupled to a 6130 single quadrupole mass spectrometer. NMR spectra were acquired on Bruker Avance III 600 and 500 MHz spectrometers at 300 K. MS/MS measurements of lindolins were carried out on a Thermo Scientific Q Exactive Plus mass spectrometer. LM profiles were analyzed on a Waters Acquity UHPLC system coupled to a QTRAP 5500 mass spectrometer (ABSciex, Darmstadt, Germany) and equipped with a Turbo V Source and electrospray ionization.

### Protein Production

Heterologous protein production was performed in *E. coli* SoluBL21 × pNH07 and BL21 × pNH08 carrying pET28a-derived expression vectors for *linA* and *linB*, respectively (Table S1).^33^ Batch fermentations were performed in Riesenberg’s modified minimal medium^62^ (6.64 g L^–1^ KH_2_PO_4_, 1.6 g L^–1^ (NH_4_)_2_HPO_4_, 0.85 g L^–1^ citric acid, 1 g L^–1^ NH_4_Cl, 60 mg L^–1^ Fe-citrate, 3 mg L^–1^ H_3_BO_3_, 15 mg L^–1^ MnCl_2_ · 4 H_2_O_,_ 8.4 mg L^–1^ EDTA·2H_2_O, 1.5 mg L^–1^ CuCl_2_·2H_2_O, 2.5 mg L^–1^ Na_2_MoO_4_·2H_2_O, 2.5 mg L^–1^ CoCl_2_·6H_2_O, 8 mg L^–1^ Zn­(CH_3_COO)_2_·2H_2_O, 1.5 g L^–1^ MgSO_4_, 35 g L^–1^ glucose, 50 μg mL^–1^ kanamycin, 1% [v/v] SAG antifoam, pH 6.8) in 20 L (30 L-bioreactor Sartorius Biostat D-DCU-D20–3, for LinA) or in 5 L (7 L-bioreactor Sartorius Biostat B-DCU-5L DW, for LinB) at 30 °C at 200–900 rpm. After 20 h, cultures were induced with 0.5 mM isopropyl β-d-1-thiogalactopyranoside (IPTG) and the temperature was shifted to 18 °C for additional 7 h. Induction of *linB* expression in BL21xpNH08 was additionally monitored by determination of LinB activity of OD 2.0 cell equivalents (Figure S1). Cell biomass of each cultivation was resuspended (100 g L^–1^) in suspension buffer (50 mM sodium phosphate, 300 mM NaCl, pH 8) and lysed twice with a homogenizer (Gaulin) at 1500–2000 bar. The pH was adjusted to 8.0 and 10 mM imidazole was added before centrifugation at 15,970*g* for 30 min. The solid phase was separated and the supernatant was centrifuged again at 38,400*g* for 30 min. The liquid phase was finally filtered (pore size 1.2 μm). Purification was implemented by fast protein liquid chromatography (Äkta pure, GE Healthcare) using a HisTrap FF 5 mL column (GE Healthcare) with a flow rate of 5 mL min^–1^. The attached proteins were eluted using suspension buffer with 250 mM imidazole. Subsequently, the obtained material was purified on a HiPrep 26/10 Desalting column (GE Healthcare) in 200 mM Tris/HCl (pH 7.8) with a flow rate of 5 mL min^–1^. The protein concentration was determined by the Pierce BCA protein Assay (Thermo Fisher) using bovine albumin as reference standard. Purification was verified by SDS polyacrylamide gel electrophoresis (Figures S2–S3).

### Combined LinA/LinB Assay

In order to test the substrate flexibility of LinA and LinB, a combined *in vitro* assay using the detection of the respective lindolins by UHPLC-MS was implemented.

In a 100 μL scale the following components were used: 200 mM Tris (pH 7.8), 20 mM MgCl_2_, 250 μM CoA, 2.5 mM ATP, 0.5 μM LinA, 0.5 μM LinB, 1 mM of the respective carboxylic acids (**1a**–**1k**) and 1 mM of anthranilic acid to analyze LinA substrate flexibility. The corresponding assay for LinB contained 1 mM indole-3-acetic acid (IAA) and the respective derivatives of anthranilic acid (**2a**–**2r**). The reactions were initiated by addition of anthranilic acid or IAA, respectively. After 3 h at 30 °C, reactions were stopped by shock-freezing in liquid nitrogen. Subsequently, the samples were lyophilized, resuspended in 100 μL methanol and centrifuged at 21,130*g*. Five μL of the supernatants were subjected to an UHPLC-MS analysis using method 1 (Table S2). Product quantification (Figure 2) was based on the area under the curve (AUC) of the extracted ion chromatograms (EICs) corresponding to the [M + H]^+^ ions of the expected products. Turnover with the native substrates (**1a** and **2a**), resulting in native lindolin A (**3a**), was defined as 100%. Each experiment was performed at least in triplicate.

To isolate lindolin congeners **3c**–**3k** the combined LinA/LinB assay was scaled up to 50–200 mL as follows: 200 mM Tris (pH 7.8), 20 mM MgCl_2_, 10 μM CoA, 10 mM ATP, 2.5 μM LinA, 2.5 μM LinB, 5 mM IAA and 5 mM of a respective derivative of anthranilic acid (**2c**–**2k**). The reaction was initiated by addition of IAA and was subsequently incubated at 30 °C and 50 rpm for 24 h. The resulting lindolins were extracted five times with 60 mL ethyl acetate. The organic layers were pooled, dried with Na_2_SO_4_, evaporated *in vacuo* and the residue was resuspended in 2–8 mL methanol for further purification.

### Purification and Structure Elucidation of Compounds **3a−k**

The known lindolins **3a** and **3b** were purified as previously described and mass spectra were identical to the published data.^33^ Lindolin derivatives **3c**–**3k** were purified with a semipreparative Agilent 1260 Infinity HPLC system equipped with a DAD. HPLC methods are listed in Table S2. Purification of **3c**, **3d** and **3e** were conducted using HPLC methods 1, 2 and 3, respectively. **3f, 3g, 3h** and **3j** were purified using HPLC method 4. An additional purification step was implemented for **3g** (method 5). **3i** and **3k** were isolated by HPLC methods 6 and 7, respectively. Analytical runs of all fractions were conducted on an UHPLC-MS system using method 1 (Table S2).

^1^H NMR experiments were carried out at 600 or 500 MHz and ^13^C experiments at 150 or 125 MHz using residual nondeuterated DMSO as internal standards (2.50 and 39.52 ppm respectively). The ^19^F NMR spectrum was recorded at 470 MHz using trifluoroacetic acid (TFA) as an internal standard (77.55 ppm). Data are summarized in Table S4.

#### Lindolin C (**3c**)

yellowish solid; yield 2.9 mg; UV/vis λ_max_ 226, 244, 282 nm; ^1^H NMR (500 MHz, DMSO-*d*_6_) δ 11.01 (s, 1H), 9.13 (s, 1H), 7.86 (m, 1H), 7.60 (d, 1H, *J* = 7.9 Hz), 7.36 (d, 1H, *J* = 8.1 Hz), 7.32 (s, 1H), 6.98 (t, 1H, *J* = 7.4 Hz), 6.81 (dd, 1H, *J* = 8.0 Hz, 1.0 Hz), 6.71 (td, 1H, *J* = 7.6 Hz, 1.0 Hz), 3.82 (s, 2H). ^13^C NMR (125 MHz, DMSO-*d*_6_) δ 169.7, 139.4, 136.1, 128.7, 128.7, 127.2, 123.9, 123.0, 120.9, 119.0, 119.0, 118.6, 118.3, 111.3, 108.5, 33.8. For detailed information see Table S5. HRESI-MS (*m*/*z*): [M + H]^+^ calcd. for C_16_H_15_N_2_O_2_ 267.1128, found 267.1123.

#### Lindolin D (**3d**)

white-yellowish solid; yield 2.9 mg; UV/vis λ_max_ 242, 280 nm; ^1^H NMR (600 MHz, DMSO-*d*_6_) δ 10.93 (s, 1H), 10.13 (s, 1H), 7.60 (d, 3H, *J* = 8.1 Hz), 7.35 (d, 1H, *J* = 8.1 Hz), 7.28 (m, 1H), 7.26 (m, 1H), 7.06 (t, 1H, *J* = 7.4 Hz), 7.01 (t, 1H, *J* = 7.3), 6.98 (t, 1H, *J* = 7.5 Hz), 3.72 (s, 2H). ^13^C NMR (150 MHz, DMSO-*d*_6_) δ 169.7, 139.4, 136.4, 136.1, 128.7, 128.7, 127.2, 123.9, 123.0, 121.0, 119.0, 119.0, 118.7, 118.4, 111.4, 108.6, 33.8. For detailed information see Table S6. HRESI-MS (*m*/*z*): [M + H]^+^ calcd. for C_16_H_15_N_2_O 251.1179, found 251.1176.

#### Lindolin E (**3e**)

white-yellowish solid; yield 6.8 mg; UV/vis λ_max_ 256, 280 nm; ^1^H NMR (500 MHz, DMSO-*d*_6_) δ 10.99 (s, 1H), 9.42 (s, 1H), 7.60 (d, 1H, *J* = 7.9 Hz), 7.59 (d, 1H, *J* = 7.9 Hz), 7.36 (d, 1H, *J* = 8.1 Hz), 7.33 (d, 1H, *J* = 7.8 Hz), 7.31 (s, 1H), 7.20 (t, 1H, *J* = 7.5), 7.09 (t, 1H, *J* = 7.3 Hz), 7.08 (t, 1H, *J* = 7.5 Hz), 6.99 (t, 1H, *J* = 7.4 Hz), 4.37 (s, 2H), 3.78 (s, 2H). ^13^C NMR (125 MHz, DMSO-*d*_6_) δ 169.8, 136.2, 135.9, 134.1, 127.4, 127.2, 127.0, 124.3, 124.2, 123.3, 121.1, 118.5, 118.5, 111.5, 108.3, 33.7. For detailed information see Table S7. HRESI-MS (*m*/*z*): [M + H]^+^ calcd. for C_17_H_17_N_2_O_2_ 281.1284, found 281.1285.

#### Lindolin F (**3f**)

white-yellowish solid; yield 3.3 mg; UV/vis λ_max_ 235, 262, 288 nm; ^1^H NMR (600 MHz, DMSO-*d*_6_) δ 11.40 (s, 1H), 11.11 (s, 1H), 8.50 (m, 1H), 7.95 (dt, 1H, *J* = 8.0 Hz, 1.4 Hz), 7.57 (td, 1H, *J* = 7.8 Hz, 1.4 Hz), 7.50 (d, 1H, *J* = 7.9 Hz), 7.40 (m, 1H), 7.38 (d, 1H, *J* = 8.1 Hz), 7.15 (td, 1H, *J* = 7.9 Hz, 1.1 Hz), 7.08 (td, 1H, *J* = 7.5 Hz, 1.0 Hz), 6.97 (td, 1H, *J* = 7.4 Hz, 1.0 Hz), 3.82 (s, 2H), 2.50 (s, 3H). ^13^C NMR (150 MHz, DMSO-*d*_6_) δ 202.2, 170.8, 139.5, 136.3, 134.3, 131.9, 127.1, 124.9, 123.1, 122.6, 121.1, 119.9, 118.6, 118.2, 111.5, 107.1, 35.0, 28.6. For detailed information see Table S8. HRESI-MS (*m*/*z*): [M + H]^+^ calcd. for C_18_H_17_N_2_O_2_ 293.1285, found 293.1281.

#### Lindolin G (**3g**)

white-yellowish solid; yield 0.9 mg; UV/vis λ_max_ 224, 253, 282, 290 nm; ^1^H NMR (600 MHz, DMSO-*d*_6_) δ 11.09 (s, 1H), 10.59 (s, 1H), 8.42 (ddd, 1H, *J* = 8.3 Hz, 5.6 Hz, 0.9 Hz), 7.85 (td, 1H, J = 7.9 Hz, 1.4 Hz), 7.58 (td, 1H, *J* = 7.9 Hz, 1.3 Hz), 7.52 (d, 1H, *J* = 7.9 Hz), 7.39 (s, 1H), 7.38 (d, 1H, *J* = 8.0 Hz), 7.14 (td, 1H, *J* = 7.6 Hz, 1.2 Hz), 7.09 (t, 1H, *J* = 7.6 Hz), 6.98 (t, 1H, *J* = 7.6 Hz), 3.83 (s, 2H), 3.65 (s, 3H). ^13^C NMR (150 MHz, DMSO-*d*_6_) δ 170.3, 167.2, 139.8, 136.4, 134.0, 130.6, 127.1, 124.8, 122.9, 121.2, 120.5, 118.7, 118.3, 116.8, 111.5, 107.2, 52.2, 34.8. For detailed information see Table S9. HRESI-MS (*m*/*z*): [M + H]^+^ calcd. for C_18_H_17_N_2_O_3_ 309.1234, found 309.1232.

#### Lindolin H (**3h**)

yellowish solid; yield 1.3 mg; UV/vis λ_max_ 230, 265, 290 nm; ^1^H NMR (600 MHz, DMSO-*d*_6_) δ 11.12 (s, 1H), 10.95 (s, 1H), 9.80 (s, 1H), 8.37 (dd, 1H, *J* = 8.0 Hz, 2.0 Hz), 7.80 (dt, 1H, J = 7.8 Hz, 1.3 Hz), 7.65 (td, 1H, *J* = 7.8 Hz, 1.3 Hz), 7.52 (d, 1H, 7.9 Hz), 7.40 (s, 1H), 7.38 (d, 1H, *J* = 8.1 Hz), 7.27 (t, 1H, *J* = 7.6 Hz), 7.08 (t, 1H, *J* = 7.4 Hz), 6.97 (t, 1H, *J* = 7.5 Hz), 3.86 (s, 2H) ^13^C NMR (150 MHz, DMSO-*d*_6_) δ 194.7, 171.2, 139.9, 136.4, 135.5, 134.5, 127.1, 124.9, 123.4, 123.2, 121.2, 120.0, 118.7, 118.2, 111.5, 107.0, 34.6. For detailed information see Table S10. HRESI-MS (*m*/*z*): [M + H]^+^ calcd. for C_17_H_15_N_2_O_2_ 279.1128, found 279.1125.

#### Lindolin I (**3i**)

white-yellowish solid; yield 2.7 mg; UV/vis λ_max_ 228, 254, 281, 288 nm; ^1^H NMR (500 MHz, DMSO-*d*_6_) δ 11.05 (s, 1H), 10.66 (s, 1H), 8.44 (m, 1H), 7.86 (dd, 1H, *J* = 7.9 Hz, 1.3 Hz), 7.58 (td, 1H, *J* = 7.9 Hz, 1.3 Hz), 7.51 (d, 1H, *J* = 7.9 Hz), 7.39 (m, 1H), 7.38 (m, 1H), 7.13 (td, 1H, *J* = 7.7 Hz, 1.1 Hz), 7.08 (td, 1H, *J* = 7.5 Hz, 1.0 Hz), 6.97 (td, 1H, J = 7.5 Hz, 0.9 Hz), 4.14 (q, 2H, *J* = 7.1 Hz), 3.83 (s, 2H), 1.20 (t, 3H, *J* = 7.1 Hz). ^13^C NMR (125 MHz, DMSO-*d*_6_) δ 170.4, 166.8, 140.0, 136.4, 134.0, 130.6, 127.1, 124.8, 122.9, 121.2, 120.4, 118.7, 118.3, 116.8, 111.5, 107.2, 61.1, 34.9, 13.9. For detailed information see Table S11. HRESI-MS (*m*/*z*): [M + H]^+^ calcd. for C_19_H_19_N_2_O_3_ 323.1390, found 323.1389.

#### Lindolin J (**3j**)

white-yellowish solid; yield 8.2 mg; UV/vis λ_max_ 228, 272 nm; ^1^H NMR (500 MHz, DMSO-*d*_6_) δ 10.99 (s, 1H), 9.86 (s, 1H), 7.86 (m, 1H), 7.61 (d, 1H, *J* = 7.8 Hz), 7.35 (d, 1H, *J* = 8.1 Hz), 7.27 (s, 1H), 7.23 (m, 1H), 7.13 (m, 2H), 7.07 (t, 1H, *J* = 7.5 Hz), 6.98 (t, 1H, *J* = 7.4 Hz), 3.81 (s, 2H). ^13^C NMR (125 MHz, DMSO-*d*_6_) δ 170.1, 153.6 (d, *J* = 245 Hz), 136.4, 127.2, 126.4 (d, *J* = 11 Hz), 125.1 (d, *J* = 7 Hz), 124.3 (d, *J* = 3 Hz), 124.1 (br), 124.0, 121.0, 118.7, 118.4, 115.4 (d, *J* = 19 Hz), 111.4, 108.4, 33.1. ^19^F NMR (470 MHz, DMSO-*d*_6_) δ −128.0. For detailed information see Table S12. HRESI-MS (*m*/*z*): [M + H]^+^ calcd. for C_16_H_14_FN_2_O 269.1085, found 269.1082.

#### Lindolin K (**3k**)

white-yellowish solid; yield 2.1 mg; UV/vis λ_max_ 234, 280 nm; ^1^H NMR (500 MHz, DMSO-*d*_6_) δ 10.94 (s, 1H), 10.21 (s, 1H), 8.09 (s, 1H), 7.80 (d, 1H, *J* = 7.9 Hz), 7.61 (d, 1H, *J* = 7.9 Hz), 7.56 (d, 1H, *J* = 7.6 Hz), 7.34 (d, 1H, *J* = 7.9 Hz), 7.28 (t, 1H, *J* = 7.7 Hz), 7.27 (s, 1H), 7.06 (t, 1H, *J* = 7.4 Hz), 6.98 (t, 1H, *J* = 7.4 Hz), 3.72 (s, 2H). ^13^C NMR (125 MHz, DMSO-*d*_6_) δ 169.8, 139.1, 128.1, 128.1, 127.2, 123.9, 123.9, 121.3, 121.0, 120.1, 118.7, 118.4, 111.4, 108.6, 33.9. For detailed information see Table S13. HRESI-MS (*m*/*z*): [M + H]^+^ calcd. for C_17_H_15_N_2_O_3_ 295.1077, found 295.1073.

### Determination of Antioomycete Activity

Compounds **3a**–**3k** were dissolved in methanol at 400 μM and 40 μM. Antioomycete activity against *P. megasperma* CBS 687.79 was tested in triplicates (for **3f, 3g** and **3h** duplicates). The oomycete was cultivated on PDB agar plates (26.5 g L^–1^ potato dextrose broth, 20 g L^–1^ agar) in the presence of **3a**–**3k** or methanol as vehicle control for 8 days and colony diameter was determined as described.^33^ Activity of **3a** at 400 μM was defined as 100%.

### Generation of Monocyte-Derived M1 Macrophages

Monocytes were isolated from leukocyte concentrates obtained from freshly withdrawn peripheral blood of healthy adult male and female donors, provided by the Institute of Transfusion Medicine, University Hospital Jena, Germany. The experimental protocol was approved by the ethical committee of the University Hospital Jena (approval no. 5050–01/17) and was performed in accordance with the relevant guidelines and regulations. This protocol has been previously described.^63^

In brief, leukocyte concentrates were mixed with dextran (derived from *Leuconostoc* spp. MW ∼ 40,000, Sigma-Aldrich) for sedimentation of erythrocytes. The supernatant was centrifuged on lymphocyte separation medium (Histopaque-1077, Sigma-Aldrich). The fraction with peripheral blood mononuclear cells was seeded in Roswell Park Memorial Institute (RPMI) 1640 medium (Sigma-Aldrich) containing 10% (v/v) heat-inactivated fetal calf serum (FCS), 100 U mL^–1^ penicillin, and 100 μg mL^–1^ streptomycin in cell culture flasks (Greiner Bio-one, Frickenhausen, Germany) and incubated for 1.5 h at 37 °C and 5% CO_2_ for adherence of monocytes. For differentiation of monocytes to macrophages and subsequent polarization toward the M1 phenotype, we used published procedures.^63,64^ Thus, M0-macrophages were generated by incubating monocytes with 20 ng mL^–1^ granulocyte-macrophage colony-stimulating factor (GM-CSF; Peprotech, Hamburg, Germany) for 6 days in RPMI 1640 medium supplemented with 10% [v/v] FCS, 2 mM l-glutamine (Biochrom/Merck, Berlin, Germany), and penicillin-streptomycin (Biochrom/Merck). Polarization was achieved by addition of 100 ng mL^–1^ lipopolysaccharides and 20 ng mL^–1^ interferon-γ (Peprotech) for another 24 h to obtain the M1 phenotype.

### Determination of Cytotoxicity on Human M1 Macrophages

M0 monocyte-derived macrophages (MDM) (10^6^ cells mL^–1^) were pretreated in a 96-well plate in RPMI 1640 medium containing 10% [v/v] heat-inactivated FCS, 100 U mL^–1^ penicillin, and 100 μg mL^–1^ streptomycin with the 50 μM of lindolins (**3a**–**3k**), tranilast (**4**) or 1 μM staurosporine (positive control), or 0.1% [v/v] DMSO (vehicle control) for 30 min at 37 °C and 5% CO_2._ Cells were then polarized by 100 ng mL^–1^ lipopolysaccharides and 20 ng mL^–1^ interferon-γ (Peprotech) for another 24 h. Finally, cells were incubated with 3-(4,5-dimethylthiazol-2-yl)-2,5-diphenyltetrazolium bromide (MTT, 5 mg mL^–1^, 20 μL; Sigma-Aldrich, Munich, Germany) in the darkness for 2 – 3 h at 37 °C and 5% CO_2._ The formazan product was solubilized with sodium dodecyl sulfate (10% in 20 mM HCl). The absorbance at λ = 570 nm was measured using a Multiskan Spectrum microplate reader (Thermo Fisher Scientific, Schwerte, Germany).

### Treatment of Human M1 Macrophages with Compounds

M0-MDM (10^6^ cells mL^–1^) were pretreated in a 12-well plate with 50 μM of lindolins (**3a–3k**), tranilast (**4**) or vehicle (0.1% DMSO) for 30 min in RPMI 1640 medium supplemented with 10% [v/v] heat-inactivated FCS, 100 U mL^–1^ penicillin, and 100 μg mL^–1^ streptomycin. Cells were then polarized with 100 ng mL^–1^ lipopolysaccharides and 20 ng mL^–1^ interferon-γ. Samples were collected after 6 h for mRNA expression and after 24 h to determine cytokine formation, COX-2 and mPGES-1 protein levels and to proceed with LM profiling (long-term treatment).

To induce LM formation, cells were finally stimulated in phosphate buffered saline (PBS; Sigma-Aldrich) buffer (supplemented with 1 mM CaCl_2_) with 1% *S. aureus* conditioned medium (SACM) for 180 min and LMs released into the supernatant were analyzed by UHPLC-MS/MS. SACM was prepared as previously published.^52^

For short-term treatment, polarized M1 macrophages were suspended in PBS buffer containing 1 mM CaCl_2,_ and were then pretreated with 30 μM lindolin A (**3a**), 3 μM celecoxib (Cayman Chemical/Biomol GmbH, Hamburg, Germany) or 30 nM vipoglanstat (Gesynta Pharma AB, Stockholm, Sweden) for 30 min. Cell were stimulated with 1% SACM for 90 min. After the indicated incubation periods, 1 mL of the supernatants was mixed with 2 mL of ice-cold methanol containing deuterium-labeled internal standards (200 nM *d*_8_-5*S*-HETE, *d*_4_-LTB_4_, *d*_5_-LXA_4_, *d*_5_-RvD2, *d*_4_-PGE_2_ and 10 μM *d*_8_-AA; Cayman Chemical/Biomol GmbH) to facilitate quantification and ensure sample recovery.

### Analysis of Cytokine Release

For measurement of the cytokine levels, cell supernatants of M1 macrophages were collected and centrifuged (21,130*g*, 4 °C, 5 min). The levels of released IL-6, TNFα and IL-1β were analyzed by ELISA kits (R&D Systems, Bio-Techne) using the manufacturer’s protocols. The amounts of cytokines were calculated using cytokine reference standard curves.

### Lipid Mediator Analysis

Solid phase extraction (SPE) and sample preparation for UHPLC-MS/MS analysis was conducted according to published procedures with minor modifications.^65^ Briefly, methanolic samples from above were stored at −20 °C for 60 min to allow protein precipitation. After centrifugation (1200*g*, 4 °C, 10 min), 9 mL acidified H_2_O was added (final pH 3.5) and samples were subjected to SPE. Solid phase cartridges (Sep-Pak Vac 6 cm^3^ 500 mg per 6 mL C18; Waters, Milford, MA) were conditioned with 6 mL methanol and 2 mL water. Samples were loaded, washed with 6 mL water and 6 mL *n*-hexane, and eluted with 6 mL methyl formate. Eluates were evaporated to dryness (TurboVap LV, Biotage, Uppsala, Sweden), and reconstituted in 100 μL methanol/water (50:50, v/v) for UHPLC-MS/MS analysis.

LMs were analyzed on an ACQUITY UHPLC system (Waters, Milford, MA, USA) coupled to a QTRAP 5500 mass spectrometer (AB Sciex, Darmstadt, Germany) equipped with a Turbo V source and electrospray ionization. Separation was achieved on an ACQUITY UPLC BEH C18 column (1.7 μm, 2.1 mm × 100 mm; Waters, Eschborn, Germany) at 50 °C with a flow rate of 0.3 mL min^–1^. The mobile phase (methanol/water/acetic acid, 42:58:0.01, v/v/v) was linearly ramped to 86:14:0.01 over 12.5 min and then to 98:2:0.01 for 3 min.^59^ The QTrap 5500 was operated in negative ionization mode using scheduled multiple reaction monitoring (MRM) with information-dependent acquisition. The scheduled MRM window was 60 s and optimized parameters for each LM were applied.^59^ The retention time and at least six diagnostic ions for each LM were verified against authentic standard (Cayman Chemical/Biomol GmbH, Hamburg, Germany). Quantification was based on linear calibration curves (*r* ^2^ ≥ 0.998; for PUFAs ≥ 0.95). Low-abundance analytes were confirmed by fragmentation pattern matching using a QTRAP 7500 mass spectrometer (Sciex, Framingham, MA, USA) and controlled by SCIEX-OS.

### RNA Isolation, cDNA Synthesis and Quantitative PCR

Total cellular RNA was extracted using the E.Z.N.A Total RNA Kit 1 (Omega Biotek, Norcross, GA, USA), and the isolated RNA was reverse transcribed into cDNA with the High-Capacity cDNA Reverse Transcription Kit with RNase Inhibitor (Thermo Fisher Scientific, Waltham, MA, USA) according to the manufacturer’s instructions. The cDNA was mixed with PerfeCTaTM SYBR Green SuperMix, ROXTM kit (Quantabio, Beverly, MA, USA), and the real-time PCR was performed on a qTOWER3G touch instrument (Analytic Jena, Jena, Germany). Real-time PCR was carried out using primers for *mPGES-1*, (5′-GGAACGACATGGAGACCATC-3′ and 5′-GATGCACTTCCTGGTCTTCC-3′), *COX2* (5′-TGCCTGATGATTGCCCGACT-3′ and 3′-TAAGCGAGGGCCAGCTTTCA-3′), and *β-actin* (5′-ACAGAGCCTCGCCTTTGCC-3′ and 5′-CCGTGGTCCCGCACTACC-3′).

### Quantification of COX-2 and mPGES-1 Protein Levels by SDS-PAGE and Western Blot

Cell lysates of M1 macrophages (10^6^ cells) were separated on polyacrylamide gels (16%). Gels were blotted onto nitrocellulose membranes (Amersham Protran Supported 0.45 μm nitrocellulose, GE Healthcare, Freiburg, Germany). The membranes were incubated with the following primary antibodies: rabbit monoclonal anti-COX-2, 1:1,000 (D5H5, #12282, Cell Signaling); rabbit polyclonal anti-mPGES-1, 1:1,000 (STJ95054, St John’s Laboratory Ltd., London, UK) and mouse monoclonal anti-β-actin, 1:1,000 (8H10D10, #3700, Cell Signaling). Immunoreactive bands were stained with following secondary antibodies: IRDye 800CW Goat anti-Rabbit IgG (H + L), 1:15,000 (926–32211, LI-COR Biosciences, Lincoln, NE); and IRDye 680LT Goat anti-Mouse IgG (H + L), 1:40,000 (926–68020, LI-COR Biosciences), and visualized by an Odyssey infrared imager (LI-COR Biosciences). Data from densitometric analysis were background corrected.

## Supplementary Material



## Data Availability

The data underlying this study are available in the Supporting Information. The original NMR data for the novel compounds (**3c**–**3k**) in this study has been deposited in the Natural Products Magnetic Resonance Database (NP-MRD; www.np-mrd.org) and can be found with the following IDs: NP0351806 (**3c**), NP0351815 (**3d**), NP0351816 (**3e**), NP0351817 (**3f**), NP0351818 (**3g**), NP0351819 (**3h**), NP0351820 (**3i**), NP0351821 (**3j**), and NP0351822 (**3k**).

## Notes

Tables of plasmids and expression strains, HPLC methods, ^13^C NMR chemical shifts of compounds **3a**–**3k**, yields of compounds **3c**–**3k**, NMR spectroscopic data of **3c**–**3k**, and Figures of LinB activity, SDS-PAGES of purified LinA and LinB, structures and exact masses of **3a**–**3k**, atom numbering and key correlations of **3c**–**3k**, extensive 1D- and 2D-NMR spectra of compounds **3c**–**3k**, yields of compounds **3c**–**3k**, antioomycete bioassays of **3a**–**3k**, interleukin and cytokine production in the presence of **3a**–**3k**, visualization of the short-term experiment, and Western Blot analysis (PDF)
