# Supplementary material for: Anti-Inflammatory Lindolin Alkaloids Repress the Transcription of the Microsomal Prostaglandin E2 Synthase‑1 Gene in Macrophages
Source: J Nat Prod. 2026 Feb 11;89(2):670–81. doi: 10.1021/acs.jnatprod.5c01488 (PMC12954838; doi:10.1021/acs.jnatprod.5c01488)
Supplement: Supplementary file 1 [file np5c01488_si_001.pdf]

Supporting Information

Anti-inflammatory lindolin alkaloids repress the transcription of the microsomal prostaglandin E2 synthase-1 gene in macrophages

Paul M. Jordan[†,‡,*,+], Johannes Rassbach[§,‖,+], Melina Gräfe[§], Lukas K. Peltner[†], Karsten Willing[††], Lukas Zenkel[†], Kerstin Günther[†], Robin Sonnabend[§,‖], Lars Regestein[††], Oliver Werz[†,‡], and Markus Gressler[§,‖,*]

[†]Pharmaceutical/Medicinal Chemistry, Institute of Pharmacy, Friedrich Schiller University Jena, Philosophenweg 14, 07743 Jena (Germany).

[‡]Jena Center for Soft Matter (JCSM), Friedrich Schiller University Jena, Philosophenweg 7, 07743 Jena (Germany).

[§]Pharmaceutical Microbiology, Institute of Pharmacy, Friedrich Schiller University Jena, Winzerlaer Strasse 2, 07745 Jena (Germany).

[‖]Pharmaceutical Microbiology, Leibniz Institute for Natural Product Research and Infection Biology, Hans Knöll Institute, Beutenbergstrasse 11a, 07745 Jena (Germany).

[††]Bio Pilot Plant, Leibniz Institute for Natural Product Research and Infection, Biology, Hans Knöll Institute, Beutenbergstrasse 11a, 07745 Jena (Germany).

Corresponding authors

* Paul M. Jordan: email: paul.jordan@uni-jena.de

* Markus Gressler: email: markus.gressler@uni-jena.de

Table of Contents

Table S1. Plasmids and expression strains used in this study....5

Table S2. HPLC methods. 6

Table S3. ^{13}C-NMR chemical shifts of compounds **3a-3k**.. 7

Table S4. Yields of compounds **3c-3k** from combined LinA/LinB assays. 8

Table S5. NMR spectroscopic data of lindolin C (**3c**) in DMSO-*d*$_6$ recorded at 500 and 125 MHz. 8

Table S6. NMR spectroscopic data of lindolin D (**3d**) in DMSO-*d*$_6$ recorded at 600 and 150 MHz. 8

Table S7. NMR spectroscopic data of lindolin E (**3e**) in DMSO-*d*$_6$ recorded at 500 and 125 MHz. 9

Table S8. NMR spectroscopic data of lindolin F (**3f**) in DMSO-*d*$_6$ recorded at 600 and 150 MHz. 9

Table S9. NMR spectroscopic data of lindolin G (**3g**) in DMSO-*d*$_6$ recorded at 600 and 150 MHz. 10

Table S10. NMR spectroscopic data of lindolin H (**3h**) in DMSO-*d*$_6$ recorded at 600 and 150 MHz. 10

Table S11. NMR spectroscopic data of lindolin I (**3i**) in DMSO-*d*$_6$ recorded at 500 and 125 MHz. 11

Table S12. NMR spectroscopic data of lindolin J (**3j**) in DMSO-*d*$_6$ recorded at 500 and 125 MHz. 11

Table S13. NMR spectroscopic data of lindolin K (**3k**) in DMSO-*d*$_6$ recorded at 500 and 125 MHz. 12

Figure S1. LinB activity after induction in *Escherichia coli* BL21×pNH08. 13

Figure S2. SDS polyacrylamide gel electrophoresis (SDS-PAGE) of purified His$_6$-tagged LinA. 14

Figure S3. SDS polyacrylamide gel electrophoresis (SDS-PAGE) of purified His$_6$-tagged LinB. 14

Figure S4. Structures and exact masses of lindolins **3a-3k**. 15

Figure S5. Atom numbering of lindolin C (**3c**). 16

Figure S6. ^{1}H-^{1}H COSY (bold bonds) and ^{1}H-^{13}C HMBC (arrows) key correlations of lindolin C (**3c**). 16
Figure S7. ^{1}H NMR spectrum of lindolin C (**3c**) in DMSO-d_6 recorded at 500 MHz. 17
Figure S8. ^{13}C NMR spectrum of lindolin C (**3c**) in DMSO-d_6 recorded at 125 MHz. 17
Figure S9. DEPT-135 NMR spectrum of lindolin C (**3c**) in DMSO-d_6. 18
Figure S10. ^{1}H-^{1}H COSY NMR spectrum of lindolin C (**3c**) in DMSO-d_6. 18
Figure S11. ^{1}H-^{13}C HSQC NMR spectrum of lindolin C (**3c**) in DMSO-d_6. 19
Figure S12. ^{1}H-^{13}C HMBC NMR spectrum of lindolin C (**3c**) in DMSO-d_6. 19
Figure S13. ^{1}H-^{1}H TOCSY NMR spectrum of lindolin C (**3c**) in DMSO-d_6. 20
Figure S14. ^{1}H-^{1}H ROESY NMR spectrum of lindolin C (**3c**) in DMSO-d_6. 20
Figure S15. Atom numbering of lindolin D (**3d**). 21
Figure S16. ^{1}H-^{1}H COSY (bold bonds) and ^{1}H-^{13}C HMBC (arrows) key correlations of lindolin D (**3d**). 21
Figure S17. ^{1}H NMR spectrum of lindolin D (**3d**) in DMSO-d_6 recorded at 600 MHz. 22
Figure S18. ^{13}C NMR spectrum of lindolin D (**3d**) in DMSO-d_6 recorded at 125 MHz. 22
Figure S19. DEPT-135 NMR spectrum of lindolin D (**3d**) in DMSO-d_6. 23
Figure S20. ^{1}H-^{1}H COSY NMR spectrum of lindolin D (**3d**) in DMSO-d_6. 23
Figure S21. ^{1}H-^{13}C HSQC NMR spectrum of lindolin D (**3d**) in DMSO-d_6. 24
Figure S22. ^{1}H-^{13}C HMBC NMR spectrum of lindolin D (**3d**) in DMSO-d_6. 24
Figure S23. ^{1}H-^{1}H TOCSY NMR spectrum of lindolin D (**3d**) in DMSO-d_6. 25
Figure S24. ^{1}H-^{1}H ROESY NMR spectrum of lindolin D (**3d**) in DMSO-d_6. 25
Figure S25. Atom numbering of lindolin E (**3e**). 26
Figure S26. ^{1}H-^{1}H COSY (bold bonds) and ^{1}H-^{13}C HMBC (arrows) key correlations of lindolin E (**3e**). 26
Figure S27. ^{1}H NMR spectrum of lindolin E (**3e**) in DMSO-d_6 recorded at 500 MHz. 27
Figure S28. ^{13}C NMR spectrum of lindolin E (**3e**) in DMSO-d_6 recorded at 125 MHz. 27
Figure S29. DEPT-135 NMR spectrum of lindolin E (**3e**) in DMSO-d_6. 28
Figure S30. ^{1}H-^{1}H COSY NMR spectrum of lindolin E (**3e**) in DMSO-d_6. 28
Figure S31. ^{1}H-^{13}C HSQC NMR spectrum of lindolin E (**3e**) in DMSO-d_6. 29
Figure S32. ^{1}H-^{13}C HMBC NMR spectrum of lindolin E (**3e**) in DMSO-d_6. 29
Figure S33. ^{1}H-^{1}H TOCSY NMR spectrum of lindolin E (**3e**) in DMSO-d_6. 30
Figure S34. Atom numbering of lindolin F (**3f**). 31
Figure S35. ^{1}H-^{1}H COSY (bold bonds) and ^{1}H-^{13}C HMBC (arrows) key correlations of lindolin F (**3f**). 31
Figure S36. ^{1}H NMR spectrum of lindolin F (**3f**) in DMSO-d_6 recorded at 600 MHz. 32
Figure S37. ^{13}C NMR spectrum of lindolin F (**3f**) in DMSO-d_6 recorded at 125 MHz. 32
Figure S38. DEPT-135 NMR spectrum of lindolin F (**3f**) in DMSO-d_6. 33
Figure S39. ^{1}H-^{1}H COSY NMR spectrum of lindolin F (**3f**) in DMSO-d_6. 33
Figure S40. ^{1}H-^{13}C HSQC NMR spectrum of lindolin F (**3f**) in DMSO-d_6. 34
Figure S41. ^{1}H-^{13}C HMBC NMR spectrum of lindolin F (**3f**) in DMSO-d_6. 34
Figure S42. ^{1}H-^{1}H TOCSY NMR spectrum of lindolin F (**3f**) in DMSO-d_6. 35
Figure S43. ^{1}H-^{1}H ROESY NMR spectrum of lindolin F (**3f**) in DMSO-d_6. 35
Figure S44. Atom numbering of lindolin G (**3g**). 36
Figure S45. ^{1}H-^{1}H COSY (bold bonds) and ^{1}H-^{13}C HMBC (arrows) key correlations of lindolin G (**3g**). 36
Figure S46. ^{1}H NMR spectrum of lindolin G (**3g**) in DMSO-d_6 recorded at 600 MHz. 37
Figure S47. ^{13}C NMR spectrum of lindolin G (**3g**) in DMSO-d_6 recorded at 125 MHz. 37
Figure S48. DEPT-135 NMR spectrum of lindolin G (**3g**) in DMSO-d_6. 38

Figure S49. ^{1}H-^{1}H COSY NMR spectrum of lindolin G (**3g**) in DMSO-d_6. 38
Figure S50. ^{1}H-^{13}C HSQC NMR spectrum of lindolin G (**3g**) in DMSO-d_6. 39
Figure S51. ^{1}H-^{13}C HMBC NMR spectrum of lindolin G (**3g**) in DMSO-d_6. 39
Figure S52. ^{1}H-^{1}H TOCSY NMR spectrum of lindolin G (**3g**) in DMSO-d_6. 40
Figure S53. ^{1}H-^{1}H ROESY NMR spectrum of lindolin G (**3g**) in DMSO-d_6. 40
Figure S54. Atom numbering of lindolin H (**3h**). 41
Figure S55. ^{1}H-^{1}H COSY (bold bonds) and ^{1}H-^{13}C HMBC (arrows) key correlations of lindolin H (**3h**). 41
Figure S56. ^{1}H NMR spectrum of lindolin H (**3h**) in DMSO-d_6 recorded at 600 MHz. 42
Figure S57. ^{13}C NMR spectrum of lindolin H (**3h**) in DMSO-d_6 recorded at 125 MHz. 42
Figure S58. DEPT-135 NMR spectrum of lindolin H (**3h**) in DMSO-d_6. 43
Figure S59. ^{1}H-^{1}H COSY NMR spectrum of lindolin H (**3h**) in DMSO-d_6. 43
Figure S60. ^{1}H-^{13}C HSQC NMR spectrum of lindolin H (**3h**) in DMSO-d_6. 44
Figure S61. ^{1}H-^{13}C HMBC NMR spectrum of lindolin H (**3h**) in DMSO-d_6. 44
Figure S62. ^{1}H-^{1}H TOCSY NMR spectrum of lindolin H (**3h**) in DMSO-d_6. 45
Figure S63. ^{1}H-^{1}H ROESY NMR spectrum of lindolin H (**3h**) in DMSO-d_6. 45
Figure S64. Atom numbering of lindolin I (**3i**). 46
Figure S65. ^{1}H-^{1}H COSY (bold bonds) and ^{1}H-^{13}C HMBC (arrows) key correlations of lindolin I (**3i**). 46
Figure S66. ^{1}H NMR spectrum of lindolin I (**3i**) in DMSO-d_6 recorded at 500 MHz. 47
Figure S67. ^{13}C NMR spectrum of lindolin I (**3i**) in DMSO-d_6 recorded at 125 MHz. 47
Figure S68. DEPT-135 NMR spectrum of lindolin I (**3i**) in DMSO-d_6. 48
Figure S69. ^{1}H-^{1}H COSY NMR spectrum of lindolin I (**3i**) in DMSO-d_6. 48
Figure S70. ^{1}H-^{13}C HSQC NMR spectrum of lindolin I (**3i**) in DMSO-d_6. 49
Figure S71. ^{1}H-^{13}C HMBC NMR spectrum of lindolin I (**3i**) in DMSO-d_6. 49
Figure S72. ^{1}H-^{1}H TOCSY NMR spectrum of lindolin I (**3i**) in DMSO-d_6. 50
Figure S73. ^{1}H-^{1}H ROESY NMR spectrum of lindolin I (**3i**) in DMSO-d_6. 50
Figure S74. Atom numbering of lindolin J (**3j**). 51
Figure S75. ^{1}H-^{1}H COSY (bold bonds) and ^{1}H-^{13}C HMBC (arrows) key correlations of lindolin J (**3j**). 51
Figure S76. ^{1}H NMR spectrum of lindolin J (**3j**) in DMSO-d_6 recorded at 500 MHz. 52
Figure S77. ^{19}F NMR spectrum of lindolin J (**3j**) in DMSO-d_6 recorded at 470 MHz. 52
Figure S78. ^{13}C NMR spectrum of lindolin J (**3j**) in DMSO-d_6 recorded at 125 MHz. 53
Figure S79. DEPT-135 NMR spectrum of lindolin J (**3j**) in DMSO-d_6. 53
Figure S80. ^{1}H-^{1}H COSY NMR spectrum of lindolin J (**3j**) in DMSO-d_6. 54
Figure S81. ^{1}H-^{13}C HSQC NMR spectrum of lindolin J (**3j**) in DMSO-d_6. 54
Figure S82. ^{1}H-^{13}C HMBC NMR spectrum of lindolin J (**3j**) in DMSO-d_6. 55
Figure S83. ^{1}H-^{1}H TOCSY NMR spectrum of lindolin J (**3j**) in DMSO-d_6. 55
Figure S84. Atom numbering of lindolin K (**3k**). 56
Figure S85. ^{1}H-^{1}H COSY (bold bonds) and ^{1}H-^{13}C HMBC (arrows) key correlations of lindolin K (**3k**). 56
Figure S86. ^{1}H NMR spectrum of lindolin K (**3k**) in DMSO-d_6 recorded at 500 MHz. 57
Figure S87. ^{13}C NMR spectrum of lindolin K (**3k**) in DMSO-d_6 recorded at 125 MHz. 57
Figure S88. DEPT-135 NMR spectrum of lindolin K (**3k**) in DMSO-d_6. 58
Figure S89. ^{1}H-^{1}H COSY NMR spectrum of lindolin K (**3k**) in DMSO-d_6. 58
Figure S90. ^{1}H-^{13}C HSQC NMR spectrum of lindolin K (**3k**) in DMSO-d_6. 59
Figure S91. ^{1}H-^{13}C HMBC NMR spectrum of lindolin K (**3k**) in DMSO-d_6. 59

Figure S92. ^{1}H-^{1}H TOCSY NMR spectrum of lindolin K (**3k**) in DMSO-d_6. 60
Figure S93. ^{1}H-^{1}H ROESY NMR spectrum of lindolin K (**3k**) in DMSO-d_6. 60
Figure S94. Anti-oomycete activity against *Phytophthora megasperma*. 61
Figure S95. Impact of lindolins and tranilast on cytokine production in M1 macrophages. 62
Figure S96. Visualization of short-term experiment with M1 macrophages to determine COX-2 and mPGES-1 enzyme inhibition. 63
Figure S97. Western Blot to detect COX-2, β-actin and mPGES-1 in human primary M1 macrophages. 63
References 64

Table S1. Plasmids and expression strains used in this study

expression plasmid	gene	vector backbone	host	induction	ref.
pNH07	*Linderina pennispora linA* (cDNA)	pET28a (+)	*E. coli* SoluBL21	0.5 mM IPTG, 18 °C, 7 h	[1]
pNH08	*Linderina pennispora linB* (cDNA)	pET28a (+)	*E. coli* BL21	0.5 mM IPTG, 18 °C, 7 h	[1]

Table S2. HPLC methods.

purpose	method 1	method 2	method 3	method 4
instrument	Agilent Infinity II 1290	Agilent 1260	Agilent 1260	Agilent 1260
solvent A	water + 0.1% FA	water + FA 0.1%	water + 0.1% FA	water + 0.1% FA
solvent B	acetonitrile	acetonitrile	acetonitrile	acetonitrile
gradient	0 – 4 min: 5 – 72% B<br>4 – 4.5 min: 72 – 95% B<br>4.5 – 5 min: 95 – 5% B<br>5 – 5.5 min: 95 – 5% B<br>5.5 – 6 min: 5% B	0 – 20 min: 55% B,<br>20 – 21 min: 55 – 100% B,<br>21 – 24 min: 100% B,<br>24 – 25 min: 100 – 55% B,<br>25 – 33 min: 55% B	0 – 0.5 min: 45% B<br>0.5 – 10 min: 45 – 100% B,<br>10 – 11 min: 100% B,<br>11 – 11.5 min: 100 – 45% B,<br>11.5 – 19.0 min: 45% B	0 – 9 min: 50% B<br>9 – 10 min: 50 – 100% B,<br>10 – 12 min: 100% B,<br>12 – 13 min: 100 – 50% B,<br>13 – 20 min: 50% B
temperature	30 °C	12 °C	12 °C	12 °C
flow	1 mL min^{-1}	2 mL min^{-1}	2 mL min^{-1}	2 mL min^{-1}
column	Macherey-Nagel, EC50/2 Nucleodur C18 Gravity	Phenomenex Synergi Polar-RP	Phenomenex Synergi Polar-RP	Agilent Zorbax Eclipse XDB-C18
column dimension	50 × 2.0mm, 1.8 µm	250 × 10 mm, 4 µm	250 × 10 mm, 4 µm	250 × 9.4 mm, 5 µm
detection	DAD (λ = 280 nm)	DAD (λ = 280 nm)	DAD (λ = 280 nm)	DAD (λ = 280 nm)

purpose	method 5	method 6	method 7	method 8
instrument	Agilent 1260	Agilent 1260	Agilent 1260	Agilent 1200
solvent A	water + 0.1% FA	water + 0.1% FA	water + 0.1% FA	water + 0.1% FA
solvent B	acetonitrile	acetonitrile	acetonitrile	acetonitrile
gradient	0 – 12 min: 60% B<br>14 – 15 min: 60 – 100% B,<br>15 – 17 min: 100% B,<br>17 – 18 min: 100 – 60% B,<br>18 – 24.5 min: 60% B	0 – 5 min: 50% B,<br>5 – 20 min: 50 – 100% B,<br>20 – 22 min: 100% B,<br>22 – 23 min: 100 – 50% B,<br>23 – 50 min: 55% B	0 – 12 min: 70% B<br>12 – 13 min: 70 – 100% B,<br>13 – 15 min: 100% B,<br>15 – 16 min: 100 – 70% B,<br>16 – 12 min: 70% B	0 – 11 min: 50% B<br>11 – 12 min: 50 – 100% B,<br>12 – 14 min: 100% B,<br>14 – 15 min: 100 – 50% B,<br>15 – 22 min: 50% B
temperature	12 °C	12 °C	12 °C	12 °C
flow	2 mL min^{-1}	2 mL min^{-1}	2 mL min^{-1}	2 mL min^{-1}
column	Agilent Zorbax Eclipse XDB-C18	Agilent Zorbax Eclipse XDB-C18	Agilent Zorbax Eclipse XDB-C18	Agilent Zorbax Eclipse XDB-C18
column dimension	250 × 9.4 mm, 5 µm	250 × 9.4 mm, 5 µm	250 × 9.4 mm, 5 µm	250 × 9.4 mm, 5 µm
detection	DAD (λ = 280 nm)	DAD (λ = 280 nm)	DAD (λ = 280 nm)	DAD (λ = 280 nm)

Table S3. ^{13}C-NMR chemical shifts of compounds 3a-3k..

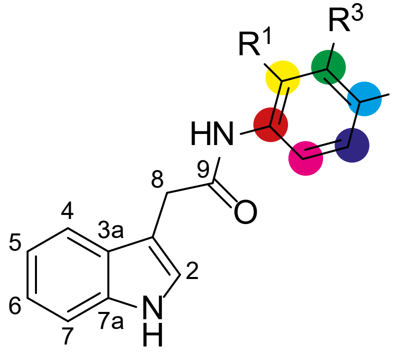

	R¹	R²	R³
lindolin A (**3a**)	COOH	H	H
lindolin B (**3b**)	COOH	OH	H
lindolin C (**3c**)	OH	H	H
lindolin D (**3d**)	H	H	H
lindolin E (**3e**)	CH_2OH	H	H
lindolin F (**3f**)	C(=O)-CH_3	H	H
lindolin G (**3g**)	$COOCH_3$	H	H
lindolin H (**3h**)	C(=O)-H	H	H
lindolin I (**3i**)	C(=O)-OCH_2CH_3	H	H
lindolin J (**3j**)	F	H	H
lindolin K (**3k**)	H	H	COOH

compound	lindolin A* **3a**	lindolin B* **3b**	lindolin C **3c**	lindolin D **3d**	lindolin E **3e**	lindolin F **3f**	lindolin G **3g**	lindolin H **3h**	lindolin I **3i**	lindolin J **3j**	lindolin K **3k**
R= 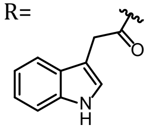	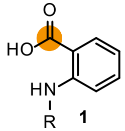 **1**	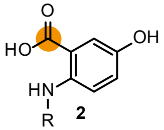 **2**	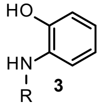 **3**	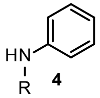 **4**	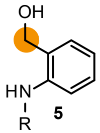 **5**	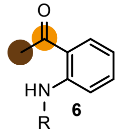 **6**	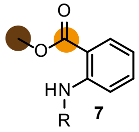 **7**	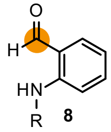 **8**	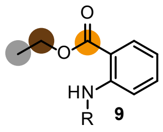 **9**	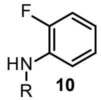 **10**	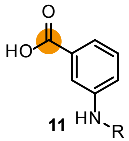 **11**
2	124.89	124.72	124.31	123.89	124.32	124.86	124.84	124.92	124.84	124.03	123.94
3	107.10	107.56	108.37	108.57	108.30	107.09	107.18	107.01	107.19	108.42	108.58
3a	127.16	127.20	127.17	127.22	127.19	127.09	127.11	127.07	127.12	127.23	127.24
4	118.18	118.30	118.68	118.69	118.48	118.20	118.30	118.21	118.30	118.72	118.73
5	118.64	118.58	118.53	118.37	118.51	118.61	118.67	118.65	118.66	118.40	118.41
6	121.13	121.09	121.12	120.97	121.08	121.14	121.21	121.19	121.21	121.01	120.99
7	111.51	111.48	111.45	111.37	111.46	111.51	111.52	111.54	111.53	111.41	111.38
7a	136.31	136.27	136.21	136.11	136.24	136.31	136.35	136.35	136.38	136.14	136.12
8	35.11	34.94	33.61	33.81	33.66	34.99	34.81	34.56	34.86	33.16	33.86
9	170.46	169.53	169.68	169.72	169.81	170.78	170.33	171.17	170.37	170.13	169.78
● (yellow)	116.19	118.58	147.29	119.05	134.10	123.09	116.80	123.22	116.81	153.64	120.08
● (red)	141.05	133.15	126.59	139.42	135.87	139.47	139.83	139.88	140.00	126.36	139.06
● (magenta)	119.38	121.01	120.97	119.05	123.33	119.94	120.46	119.97	120.39	124.12	121.78
● (dark blue)	133.85	120.27	118.78	128.67	127.03	134.29	134.03	135.47	134.04	124.31	128.13
● (light blue)	122.30	152.01	124.11	123.01	124.21	122.60	122.92	123.41	122.88	125.12	123.93
● (green)	131.01	116.53	115.38	128.67	127.38	131.85	130.59	134.52	130.56	115.46	128.13
● (orange)	169.29	169.13			60.31	202.27	167.21	194.74	166.82		168.83
● (brown)						28.55	52.20		61.07		
● (grey)									13.94		

* Data are identical to lindolin A and B reference standards as described.[1]

Table S4. **Yields of compounds 3c-3k from combined LinA/LinB assays.**

compound	3c	3d	3e	3f	3g	3h	3i	3j	3k
relative yield [mg per 100 mL assay]	5.8	3.87	13.6	6.6	0.6	1.3	1.8	5.47	2.1
total yield [mg]	2.9	2.9	6.8	3.3	0.9	1.3	2.7	8.2	2.1

Table S5. **NMR spectroscopic data of lindolin C (3c) in DMSO-d_6 recorded at 500 and 125 MHz.**

	δ ^{13}C [ppm]	δ ^{1}H [ppm], M (J [Hz])	COSY	HMBC
indole-NH		11.01, s	2	2, 3, 3a, 7a
2	124.31	7.32, s	indole-NH	3, 3a, 7a, 8
3	108.37			
3a	127.17			
4	118.68	7.60, d (7.9)	5	3a, 6, 7, 7a
5	118.53	6.98, t (7.4)	4	3a, 7
6	121.12	7.07, t (7.5)	7	4, 7a
7	111.45	7.36, d (8.1)	6	3a, 5
7a	136.21			
8	33.61	3.82, s		2, 3, 3a, 9
9	169.98			
amide-NH		9.13, s		2‘, 6‘, 9
1‘	126.59			
2‘	147.29			
3‘	115.38	6.81, dd (8.0, 1.0)	4‘	1‘, 2‘, 5‘
4‘	124.11	6.87, td (7.5, 1.2)	3‘, 5‘	2‘, 6‘
5‘	118.78	6.71, td (7.6, 1.0)	4‘, 6‘	1‘, 3‘
6‘	120.97	7.86, m	5‘	2‘, 4‘

Table S6. **NMR spectroscopic data of lindolin D (3d) in DMSO-d_6 recorded at 600 and 150 MHz.**

	δ ^{13}C [ppm]	δ ^{1}H [ppm], M (J [Hz])	COSY	HMBC
indole-NH		10.93, s	2	2, 3, 3a, 7a
2	123.89	7.26, m	indole-NH	3, 3a, 7a, 8
3	108.57			
3a	127.22			
4	118.69	*7.60, d (8.1)	5	3a, 6, 7, 7a
5	118.37	6.98, t (7.5)	4	3a, 7, 7a
6	120.97	7.06, t (7.4)	7	4, 7a
7	111.37	7.35, d (8.1)	6	3a, 5
7a	136.11			
8	33.81	3.72, s		2, 3, 3a, 9
9	169.72			
amide-NH		10.13, s		2‘, 6‘, 9
1‘	139.42			
2‘	119.05	*7.60, d (8.1)	3‘	1‘, 4‘, 6‘
3‘	128.67	7.28, m	2‘, 4‘	1‘, 5‘
4‘	123.01	7.01, t (7.3)	4‘, 6‘	2‘, 6‘
5‘	128.67	7.28, m	4‘, 6‘	1‘, 3‘
6‘	119.05	*7.60, d (8.1)	5‘	1‘, 2‘, 4‘

*signals overlapping

Table S7. **NMR spectroscopic data of lindolin E (3e) in DMSO-d_6 recorded at 500 and 125 MHz.**

	δ ^{13}C [ppm]	δ ^{1}H [ppm], M (J [Hz])	COSY	HMBC
indole-NH		10.99, s	2	2, 3, 3a, 7a
2	124.32	7.31, s	indole-NH	3, 3a, 7a, 8
3	108.30			
3a	127.19			
4	118.51	7.59, d (7.9)	5	3, 3a, 6, 7, 7a
5	118.48	6.99, t (7.4)	4	3a, 7
6	121.08	7.08, t (7.5)	7	4, 7a
7	111.46	7.36, d (8.1)	6	3a, 5
7a	136.24			
8	33.66	3.78, s		2, 3, 3a, 9
9	169.81			
amide-NH			9.42	2‘, 6‘, 9
1‘	135.87			
2‘	134.10			
3‘	127.38	7.33, d (7.8)	4‘	1‘, 5‘, 7‘
4‘	124.21	7.09, t (7.3)	3‘, 5‘	2‘, 6‘
5‘	127.03	7.20, t (7.5)	4‘, 6‘	1‘, 3‘
6‘	123.33	7.60, d (7.9)	5‘	2‘, 4‘
7‘	60.31	4.37, s		1‘, 2‘, 3‘

Table S8. **NMR spectroscopic data of lindolin F (3f) in DMSO-d_6 recorded at 600 and 150 MHz.**

	δ ^{13}C [ppm]	δ ^{1}H [ppm], M (J [Hz])	COSY	HMBC
indole-NH		11.11, s	2	2, 3, 3a, 7a
2	124.86	7.40, m	indole-NH	3, 3a, 7a, 8, 9
3	107.09			
3a	127.09			
4	118.20	7.50, d (7.9)	5	3, 3a, 6, 7, 7a
5	118.61	6.97, td (7.4, 1.0)	4	3a, 7, 7a
6	121.14	7.08, td (7.5, 1.0)	7	4, 7a
7	111.51	7.38, d (8.1)	6	3a, 5
7a	136.31			
8	34.99	3.82, s		2, 3, 3a, 9
9	170.78			
amide-NH		11.40, s		2‘, 6‘, 9
1‘	139.47			
2‘	123.09			
3‘	131.85	7.95, dt (8.0, 1.4)	4‘	1‘, 5‘, 7‘
4‘	122.60	7.15, td (7.9 1.1)	3‘, 5‘	2‘, 6‘
5‘	134.29	7.57, td (7.8, 1.4)	4‘, 6‘	1‘, 3‘
6‘	119.94	8.50, m	5‘	2‘, 4‘, 7‘
7‘	202.27			
8‘	28.55	2.50, s		2‘, 3‘, 7‘

Table S9. **NMR spectroscopic data of lindolin G (3g) in DMSO-d_6 recorded at 600 and 150 MHz.**

	δ ^{13}C [ppm]	δ ^{1}H [ppm], M (J [Hz])	COSY	HMBC
indole-NH		11.09, s	2	2, 3, 3a, 7a
2	124.84	7.39, s	indole-NH	3, 3a, 7a, 8
3	107.18			
3a	127.11			
4	118.30	7.52, d (7.9)	5	3, 3a, 6, 7, 7a
5	118.67	6.98, t (7.59)	4	3a, 7, 7a
6	121.21	7.09, t (7.6)	7	4, 7a
7	111.52	7.38, d (8.0)	6	3a, 5
7a	136.35			
8	34.81	3.83, s		2, 3, 3a, 9
9	170.33			
amide-NH		10.59, s		1‘, 3‘, 9
1‘	116.80			
2‘	139.83			
3‘	120.46	8.42, ddd (8.3, 5.6, 0.9)	4‘	1‘, 5‘, 7‘
4‘	134.03	7.58, td (7.9, 1.3)	3‘, 5‘	2‘, 6‘
5‘	122.92	7.14, td (7.6, 1.2)	4‘, 6‘	1‘, 3‘
6‘	130.59	7.85, dd (7.9, 1.4)	5‘	2‘, 4‘, 7‘
7‘	167.21			
8‘	52.20	3.65, d (1.0)		7‘

Table S10. **NMR spectroscopic data of lindolin H (3h) in DMSO-d_6 recorded at 600 and 150 MHz.**

	δ ^{13}C [ppm]	δ ^{1}H [ppm], M (J [Hz])	COSY	HMBC
indole-NH		11.12, s	2	2, 3, 3a, 7a
2	124.92	7.40, s	indole-NH	3, 3a, 7a, 8, 9
3	107.01			
3a	127.07			
4	118.21	7.52, d (7.9)	5	3, 3a, 6, 7, 7a
5	118.65	6.97, t (7.5)	4	3a, 7, 7a
6	121.19	7.08, t (7.4)	7	4, 7a
7	111.54	7.38, d (8.1)	6	3a, 5
7a	136.35			
8	34.56	3.86, s		2, 3, 3a, 9
9	171.17			
amide-NH		10.95, s		2‘, 6‘, 9
1‘	139.88			
2‘	123.22			
3‘	134.52	7.80, dt (7.8, 1.3)	4‘	1‘, 5‘, 7‘
4‘	123.41	7.27, t (7.6)	3‘, 5‘	2‘, 6‘
5‘	135.47	7.65, td (7.8, 1.3)	4‘, 6‘	1‘, 3‘
6‘	119.97	8.37, dd (8.0, 2.0)	5‘	2‘, 4‘
7‘	194.74	9.80, s		1‘, 2‘, 3‘

Table S11. NMR spectroscopic data of lindolin I (3i) in DMSO-d_6 recorded at 500 and 125 MHz.

	δ ^{13}C [ppm]	δ ^{1}H [ppm], M (J [Hz])	COSY	HMBC
indole-NH		11.05, s	2	2, 3, 3a, 7a
2	124.84	7.39, m	indole-NH	3, 3a, 7a, 8
3	107.19			
3a	127.12			
4	118.30	7.51, d (7.9)	5	3, 3a, 6, 7, 7a
5	118.66	6.97, td (7.5, 0.9)	4	3a, 7, 7a
6	121.21	7.08, td (7.5, 1.0)	7	4, 7a
7	111.53	7.38, m	6	3a, 5
7a	136.38			
8	34.86	3.83, s		2, 3, 3a, 9
9	170.37			
amide-NH		10.66, s		1‘, 3‘, 9
1‘	116.81			
2‘	140.00			
3‘	120.39	8.44, m	4‘	1‘, 5‘, 7‘
4‘	134.04	7.58, td (7.9, 1.3)	3‘, 5‘	2‘, 6‘
5‘	122.88	7.13, td (7.7, 1.1)	4‘, 6‘	1‘, 3‘
6‘	130.56	7.86, dd (7.9, 1.3)	5‘	2‘, 4‘, 7‘
7‘	166.82			
8‘	61.07	4.14, q (7.1)	9‘	7‘, 9‘
9‘	13.94	1.20, t (7.1)	8‘	8‘

Table S12. NMR spectroscopic data of lindolin J (3j) in DMSO-d_6 recorded at 500 and 125 MHz.

	δ ^{13}C [ppm], M (J [Hz])*	δ ^{1}H [ppm], M (J [Hz])	δ ^{19}F [ppm]**	COSY	HMBC
indole-NH		10.99, s		2	2, 3, 3a, 7a
2	124.03	7.27, s		indole-NH	3, 3a, 7a, 8
3	108.42				
3a	127.23				
4	118.72	7.61, d (7.8)		5	3a, 6, 7a
5	118.40	6.98, t (7.4)		4	3a, 7, 7a
6	121.01	7.07, t (7.5)		7	4, 7a
7	111.41	7.35, d (8.1)		6	3a, 5
7a	136.41				
8	33.16	3.81, s			2, 3, 3a, 9
9	170.13				
amide-NH		9.86, s			2‘, 6‘, 9
1‘	126.36, d (11)				
2‘	153.64, d (245)		-128.04		
3‘	115.46, d (19)	7.23, m		4‘	1‘, 2‘, 5‘
4‘	125.12, d (7)	7.13, m***		3‘, 5‘	2‘, 6‘
5‘	124.31, d (3)	7.13, m***		4‘, 6‘	3‘
6‘	124.12, br	7.86, m		5‘	2‘, 4‘

* ^{19}F-^{13}C-couplings in accordance to Weigert *et al.*[2]
**recorded at 470 MHz and TFA signal referenced to -77.55 ppm
*** signals overlapping

Table S13. NMR spectroscopic data of lindolin K (3k) in DMSO-d_6 recorded at 500 and 125 MHz.

	δ ^{13}C [ppm]	δ ^{1}H [ppm], M (J [Hz])	COSY	HMBC
indole-NH		10.94, s	2	2, 3, 3a, 7a
2	123.93*	7.27, s	indole-NH	3, 3a, 7a, 8
3	108.58			
3a	127.24			
4	118.73	7.61, d (7.9)	5	3, 3a, 6, 7a
5	118.41	6.98, t (7.4)	4	3a, 7
6	120.99	7.06, t (7.4)	7	4, 7a
7	111.38	7.34, d (7.9)	6	3a, 5
7a	136.12			
8	33.86	3.72, s		2, 3, 3a, 9
9	169.78			
amide-NH		10.21, s		2‘, 4‘, 9
1‘	128.13**			
2‘	120.08	8.09, s		6‘, 7‘
3‘	139.06			
4‘	121.28	7.80, d (7.9)	5‘	2‘, 6‘
5‘	128.13*	7.28, t (7.7)	4‘, 6‘	3‘
6‘	123.93*	7.56, d (7.6)	5‘	2‘, 4‘, 7‘
7‘	168.83			

*signals overlapping

** Weak signal. Assignment is tentative and based on comparison with data reported by Krauss et al.[3]

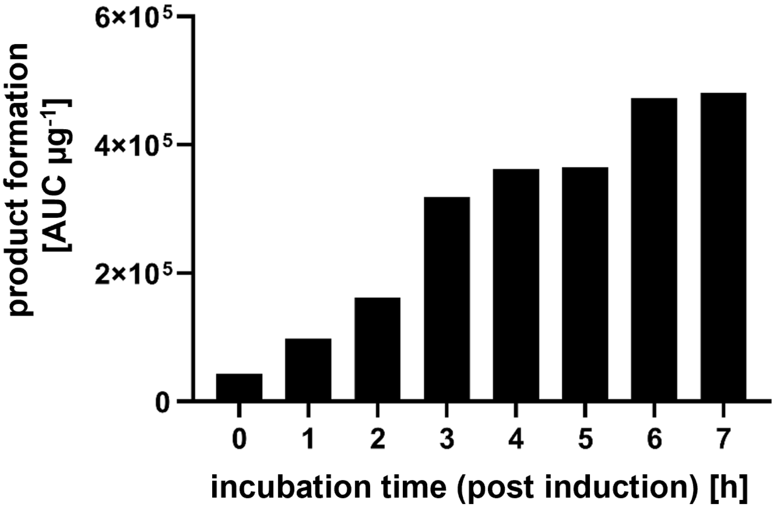

Figure S1. LinB activity after induction in *Escherichia coli* BL21×pNH08.

Cell samples corresponding to an optical density (OD_{600}) 2.0 were collected hourly after induction. Cells were lyzed and the resulting cell-free protein crude extracts containing LinB were tested regarding their ability to produce lindolin A (3a) using the LinB assay described.[1] The area under the curve for the product (**3a**) was calculated per µg protein in the crude extracts.

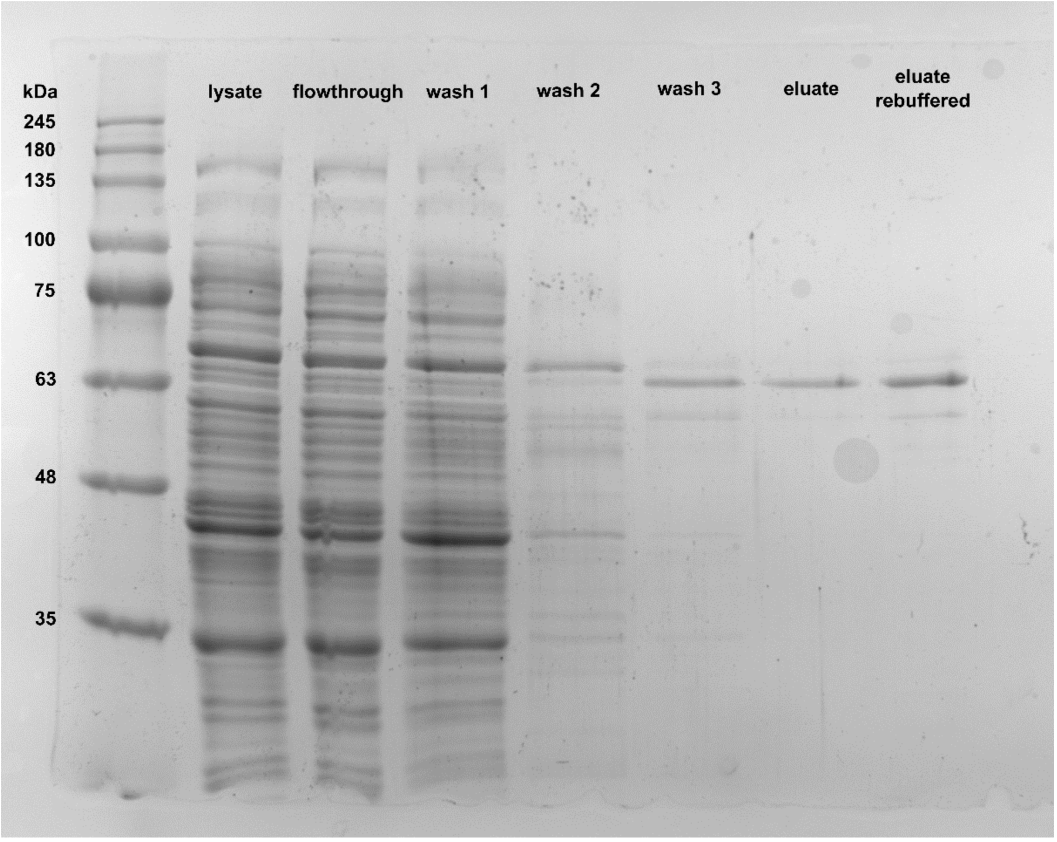

Figure S2. SDS polyacrylamide gel electrophoresis (SDS-PAGE) of purified His$_6$-tagged LinA.

The calculated protein mass for LinA is 60.3 kDa (*C*-His$_6$-tagged: 61.8 kDa). The lanes indicate: the cell-free cell lysate, column flow through, wash fractions 1- 3 (with 10, 20 and 40 mM imidazole), elution fraction (with 250 mM imidazole) and the rebuffered, purified enzyme (in Tris/HCl 100 mM, pH 7.4). Acrylamide: 12% [v/v].

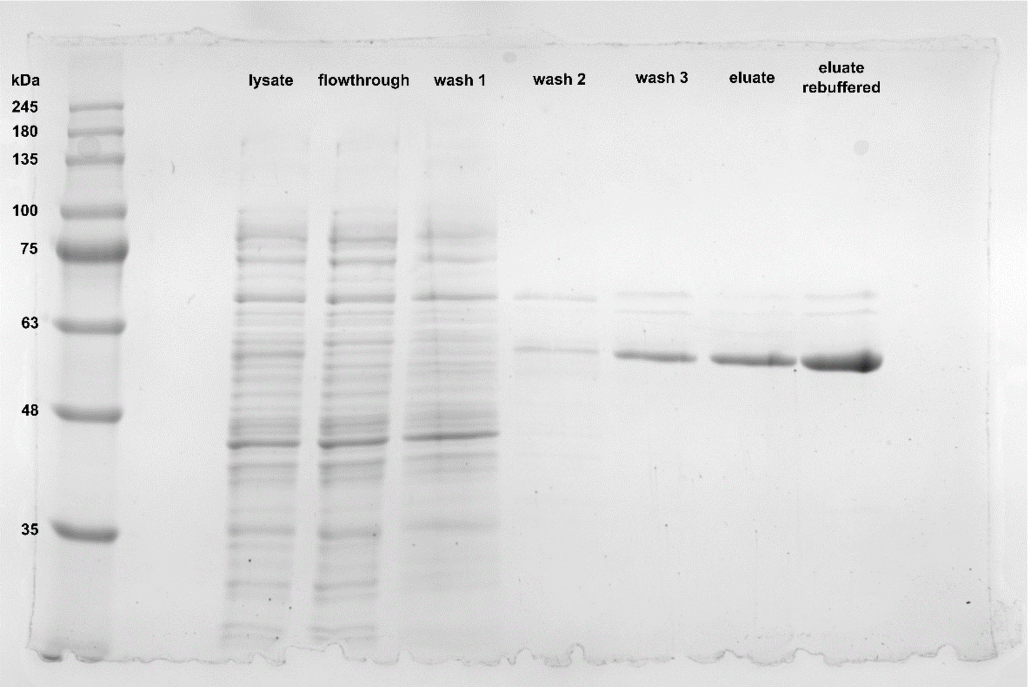

Figure S3. SDS polyacrylamide gel electrophoresis (SDS-PAGE) of purified His$_6$-tagged LinB.

The calculated protein mass for LinB is 53.5 kDa (*N*-His$_6$-tagged: 56.0 kDa). The lanes indicate: the cell-free cell lysate, column flow through, wash fractions 1- 3 (with 10, 20 and 40 mM imidazole), elution fraction (with 250 mM imidazole) and the rebuffered, purified enzyme (in Tris/HCl 100 mM, pH 7.4). Acrylamide: 12% [v/v].

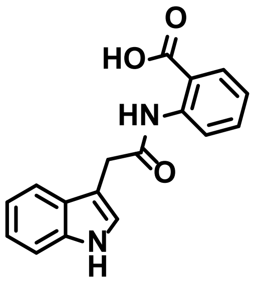

lindolin A (3a)
sum formula: $C_{17}H_{14}N_2O_3$
calc. *m/z* 295.1077 [M+H]$^+$
found *m/z* 295.1078 [M+H]$^+$

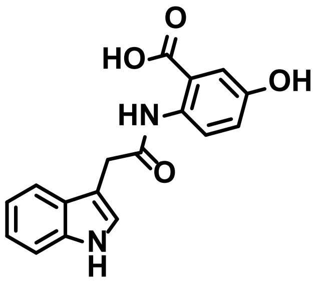

lindolin B (3b)
sum formula: $C_{17}H_{14}N_2O_4$
calc. *m/z* 311.1026 [M+H]$^+$
found *m/z* 311.1023 [M+H]$^+$

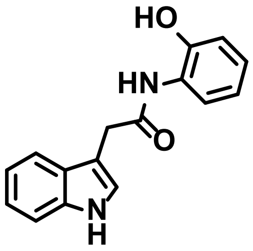

lindolin C (3c)
sum formula: $C_{16}H_{14}N_2O_2$
calc. *m/z* 267.1128 [M+H]$^+$
found *m/z* 267.1123 [M+H]$^+$

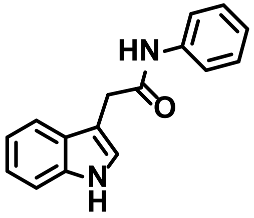

lindolin D (3d)
sum formula: $C_{16}H_{14}N_2O$
calc. *m/z* 251.1179 [M+H]$^+$
found *m/z* 251.1176 [M+H]$^+$

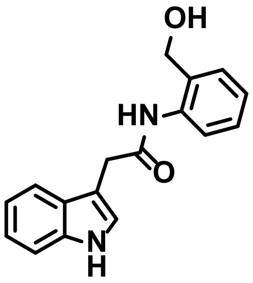

lindolin E (3e)
sum formula: $C_{17}H_{16}N_2O_2$
calc. *m/z* 281.1284 [M+H]$^+$
found *m/z* 281.1285 [M+H]$^+$

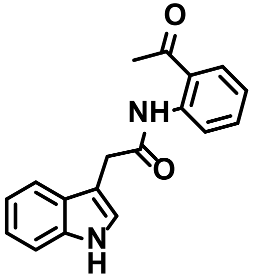

lindolin F (3f)
sum formula: $C_{18}H_{16}N_2O_2$
calc. *m/z* 293.1285 [M+H]$^+$
found *m/z* 293.1281 [M+H]$^+$

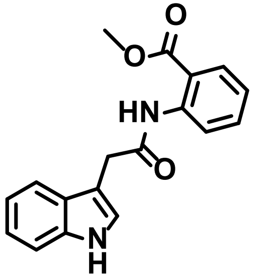

lindolin G (3g)
sum formula: $C_{18}H_{16}N_2O_3$
calc. *m/z* 309.1234 [M+H]$^+$
found *m/z* 309.1232 [M+H]$^+$

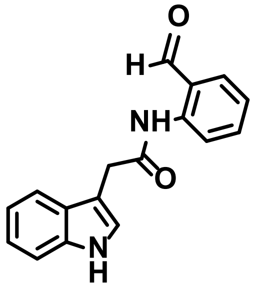

lindolin H (3h)
sum formula: $C_{17}H_{14}N_2O_2$
calc. *m/z* 279.1128 [M+H]$^+$
found *m/z* 279.1125 [M+H]$^+$

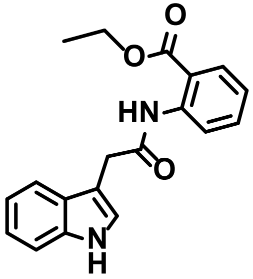

lindolin I (3i)
sum formula: $C_{19}H_{18}N_2O_3$
calc. *m/z* 323.1390 [M+H]$^+$
found *m/z* 323.1389 [M+H]$^+$

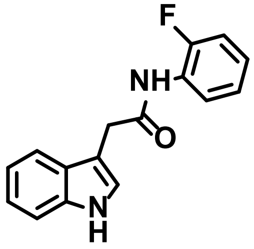

lindolin J (3j)
sum formula: $C_{16}H_{13}FN_2O$
calc. *m/z* 269.1085 [M+H]$^+$
found *m/z* 269.1082 [M+H]$^+$

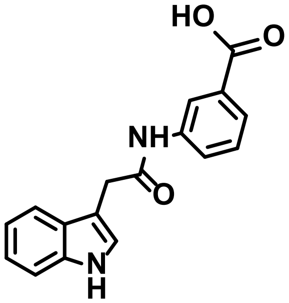

lindolin K (3k)
sum formula: $C_{17}H_{14}N_2O_3$
calc. *m/z* 295.1077 [M+H]$^+$
found *m/z* 295.1073 [M+H]$^+$

Figure S4. Structures and exact masses of lindolins 3a-3k.

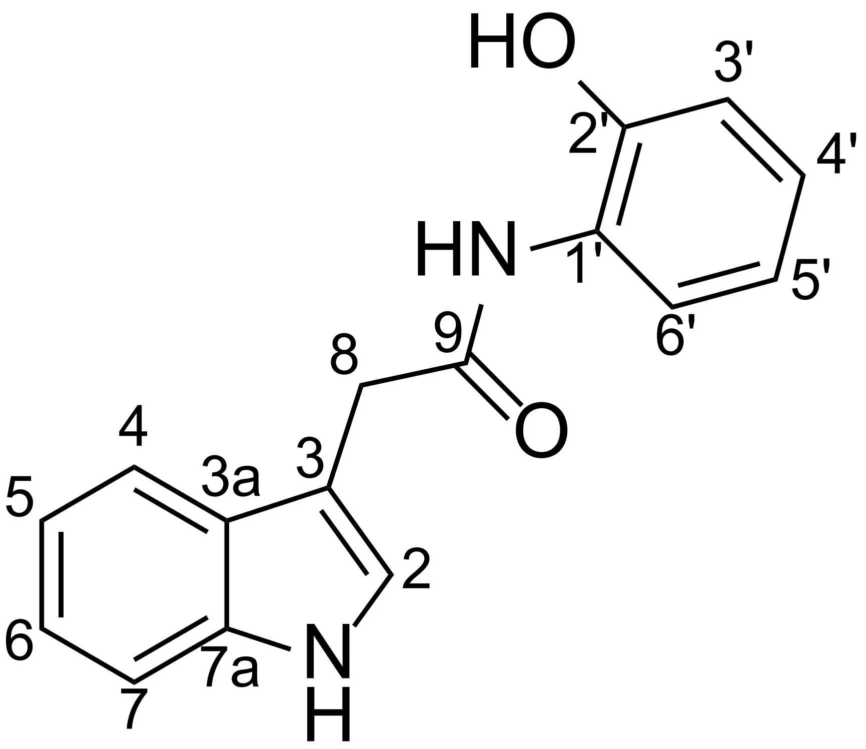

Figure S5. **Atom numbering of lindolin C (3c).**

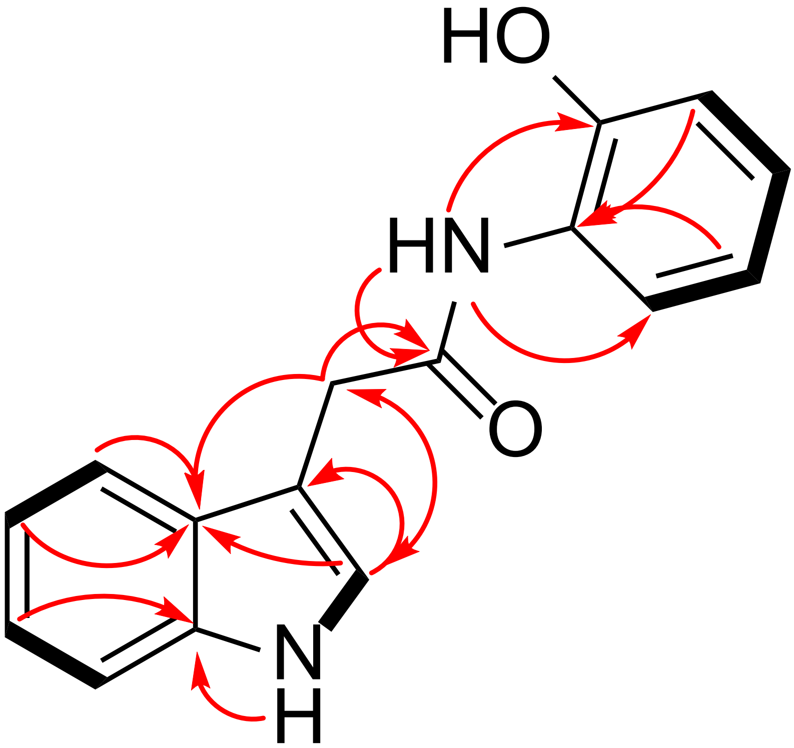

Figure S6. **^{1}H-^{1}H COSY (bold bonds) and ^{1}H-^{13}C HMBC (arrows) key correlations of lindolin C (3c).**

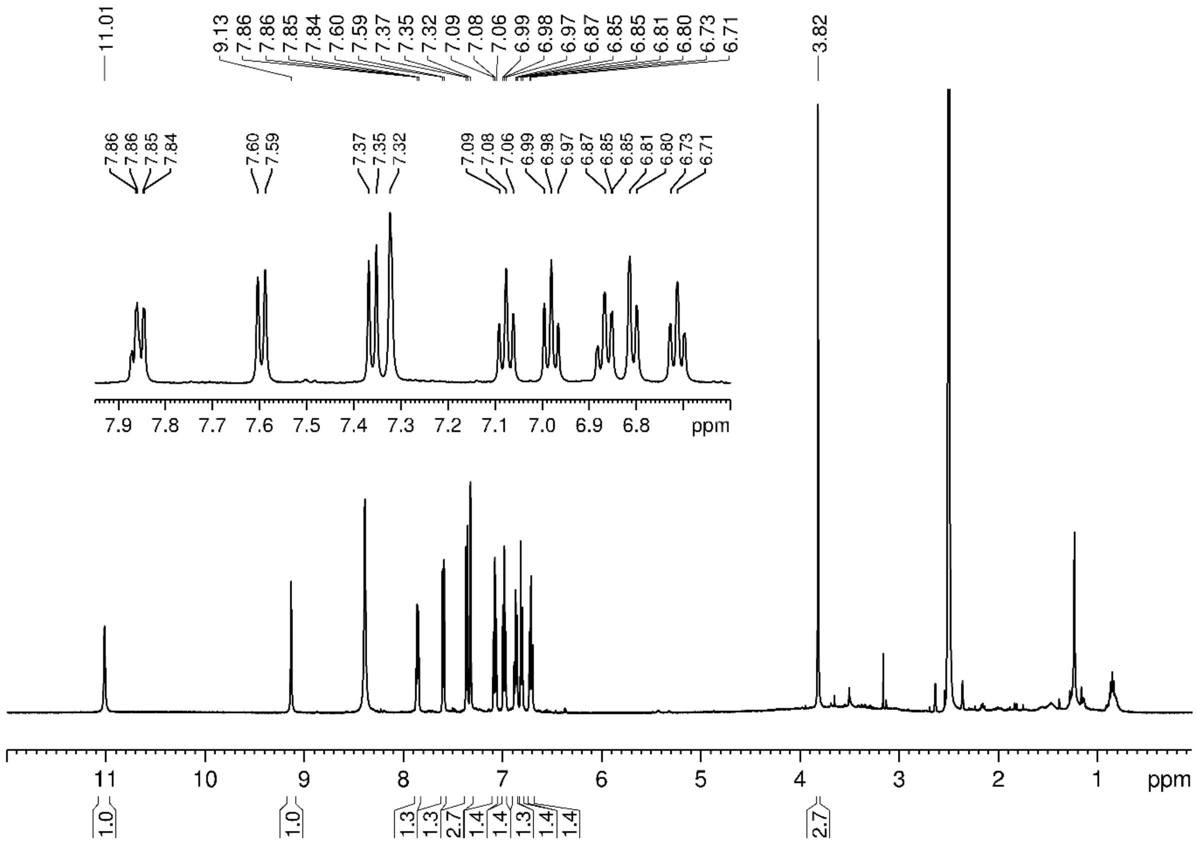

Figure S7. **^{1}H NMR spectrum of lindolin C (3c) in DMSO-d_6 recorded at 500 MHz.**

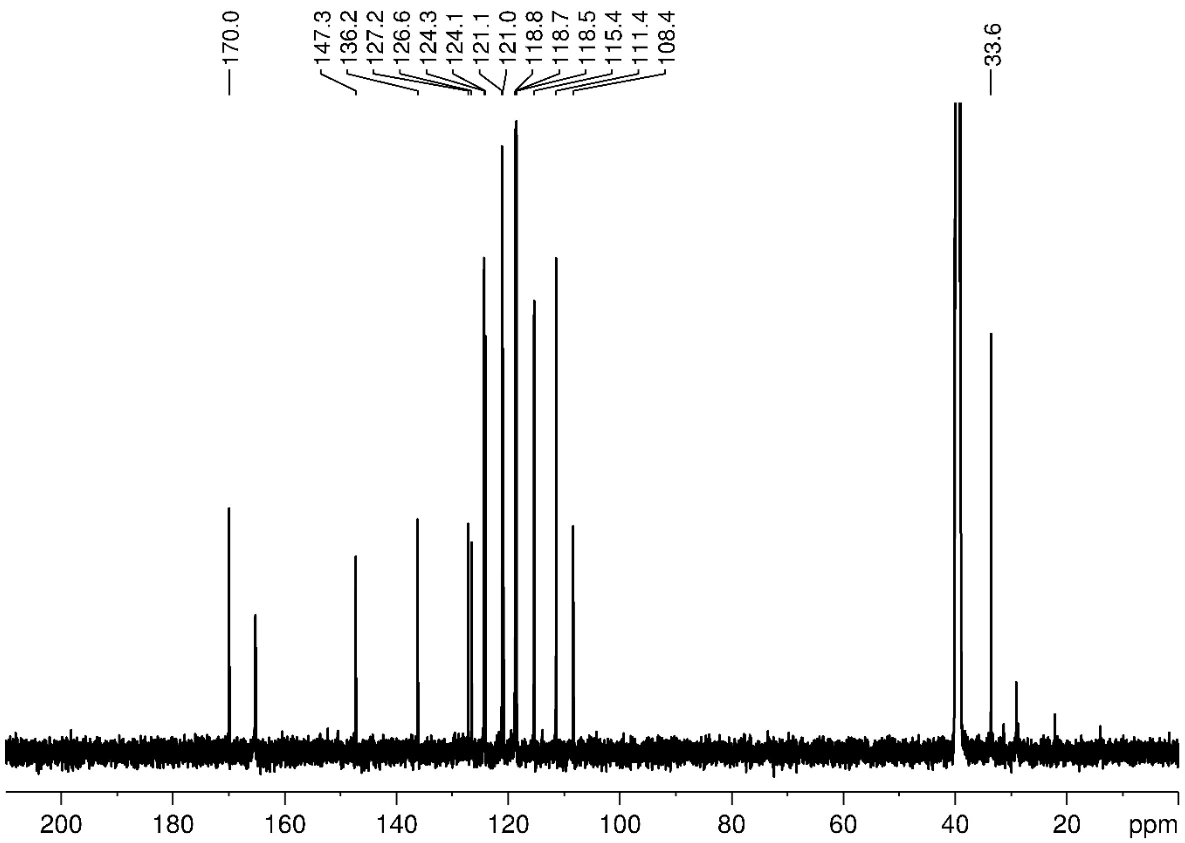

Figure S8. **^{13}C NMR spectrum of lindolin C (3c) in DMSO-d_6 recorded at 125 MHz.**

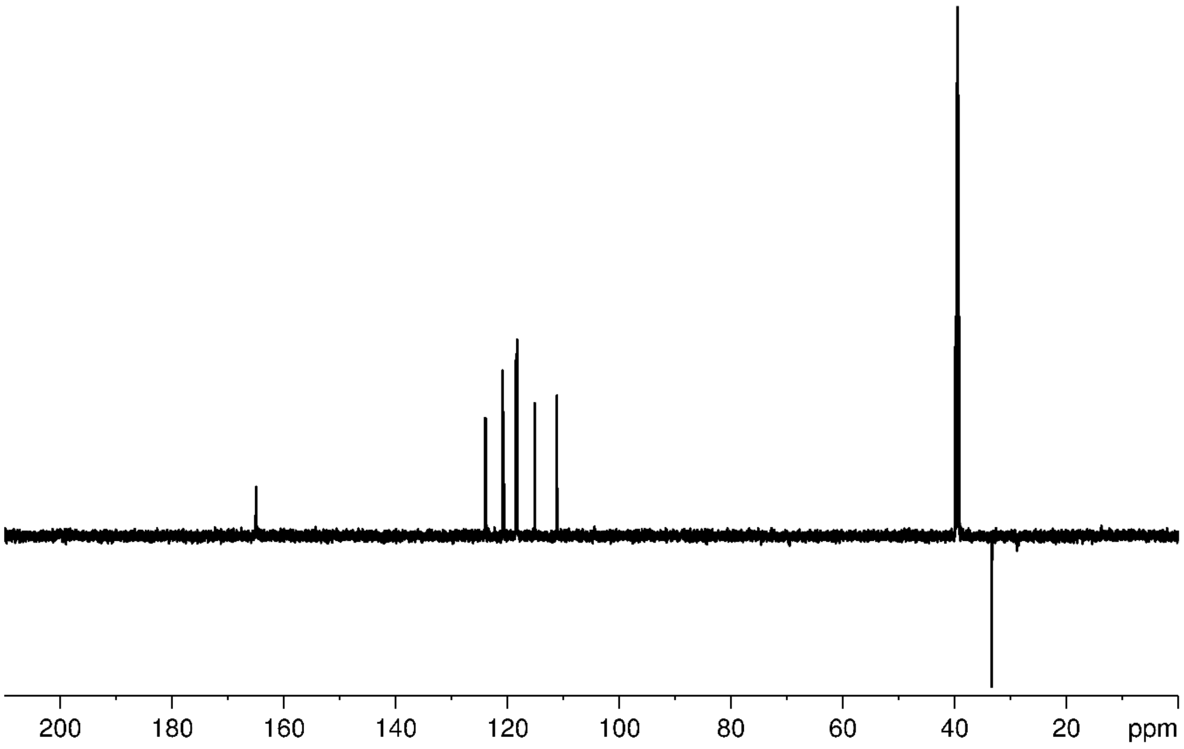

Figure S9. **DEPT-135 NMR spectrum of lindolin C (3c) in DMSO-*d*$_6$.**

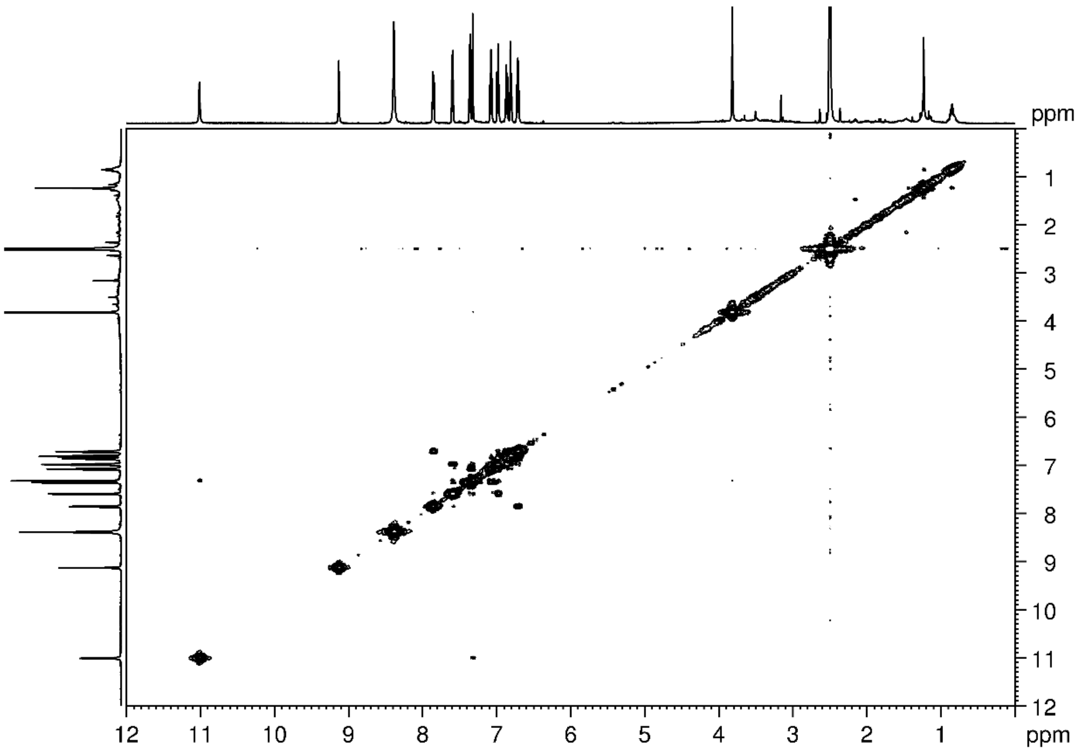

Figure S10. **^{1}H-^{1}H COSY NMR spectrum of lindolin C (3c) in DMSO-*d*$_6$.**

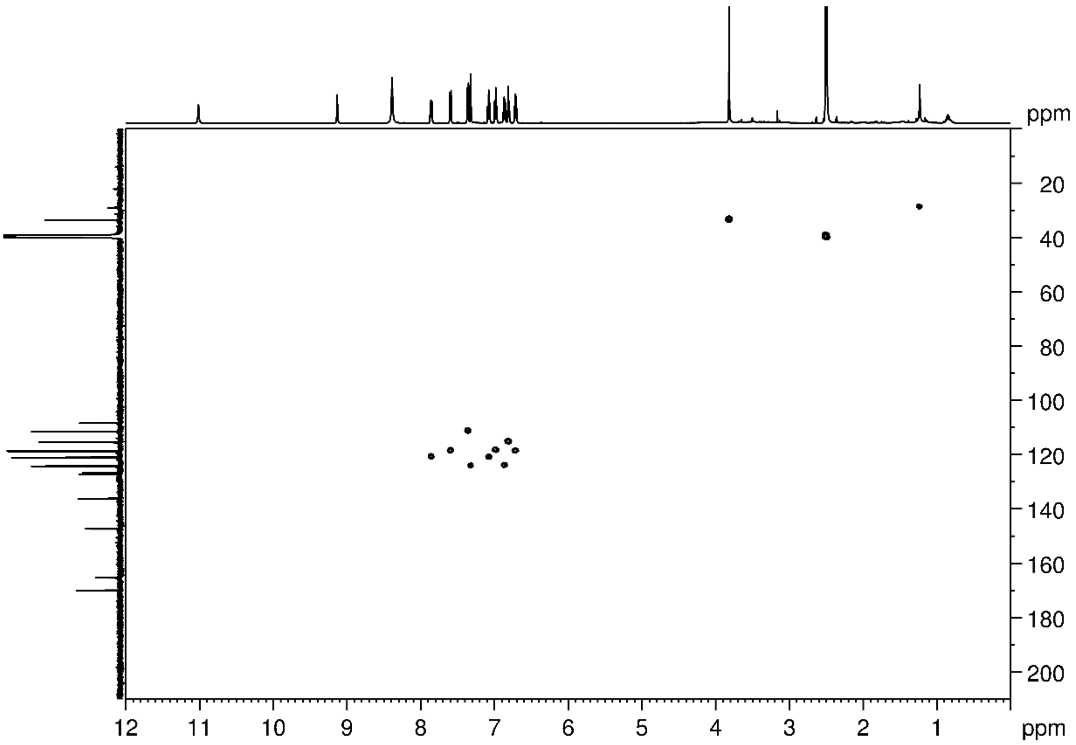

Figure S11. ^{1}H-^{13}C **HSQC NMR spectrum of lindolin C (3c) in DMSO-*d*$_6$.**

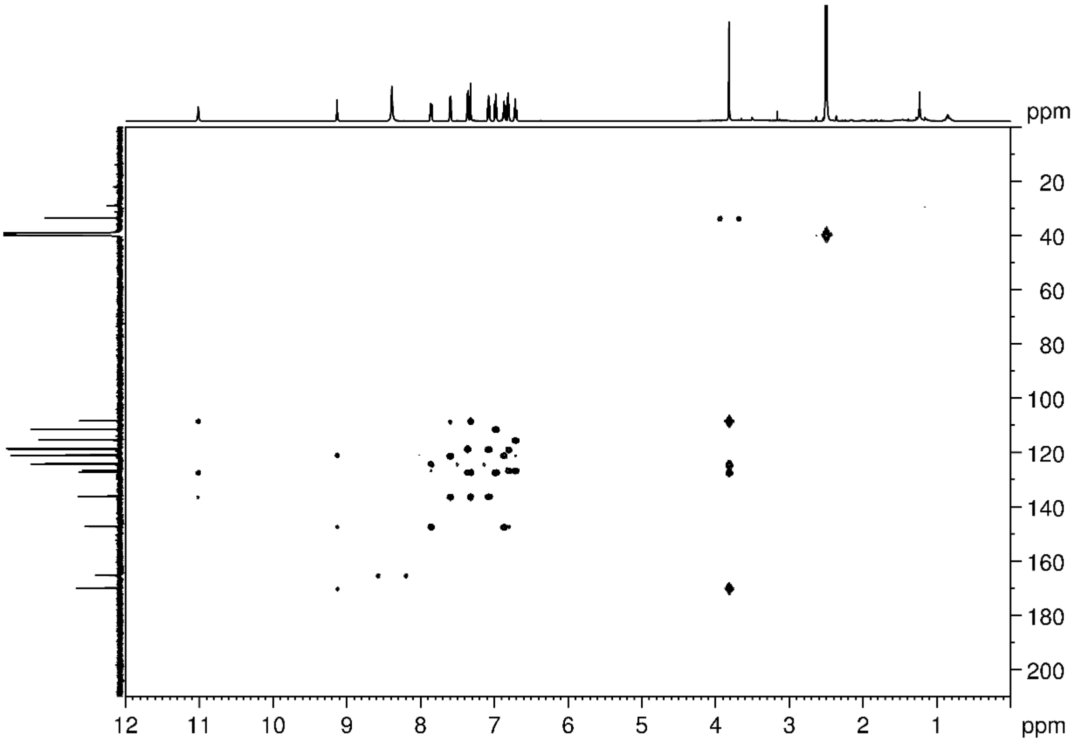

Figure S12. ^{1}H-^{13}C **HMBC NMR spectrum of lindolin C (3c) in DMSO-*d*$_6$.**

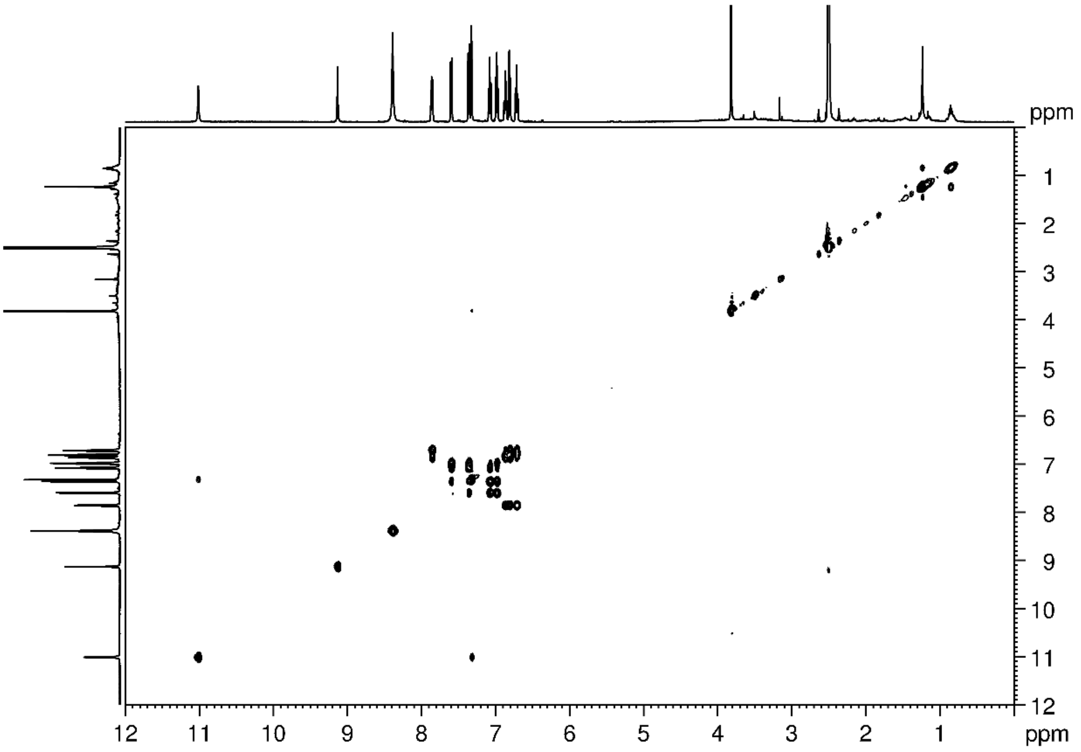

Figure S13. ^{1}H-^{1}H TOCSY NMR spectrum of lindolin C (3c) in DMSO-d_6.

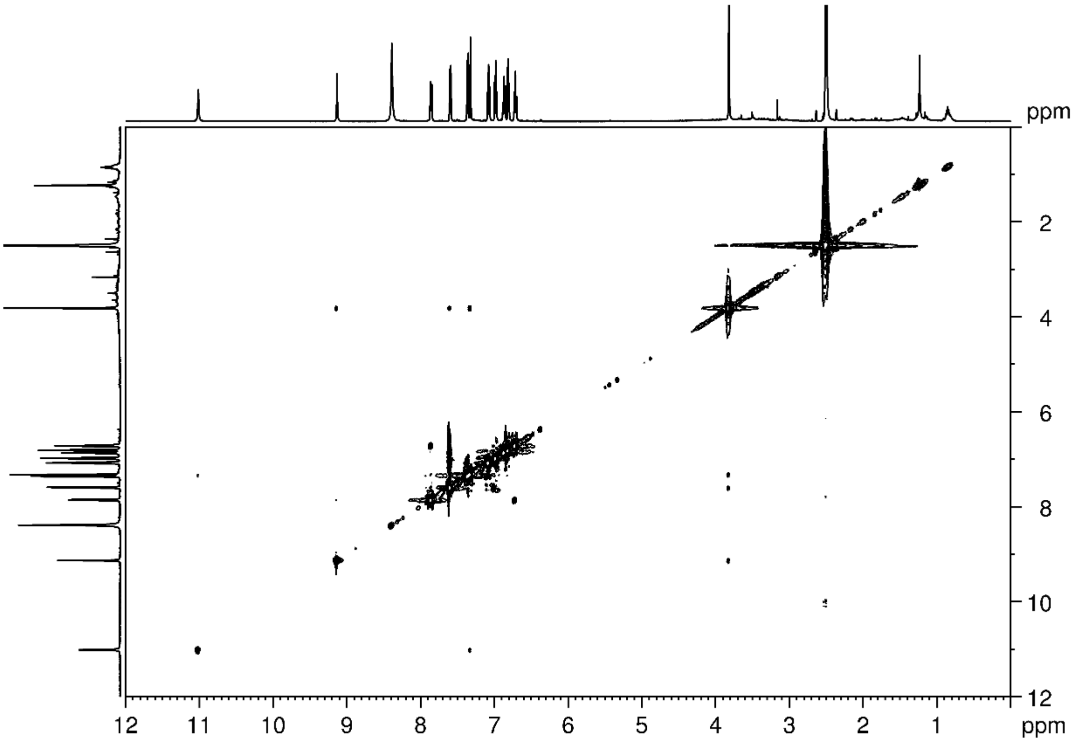

Figure S14. ^{1}H-^{1}H ROESY NMR spectrum of lindolin C (3c) in DMSO-d_6.

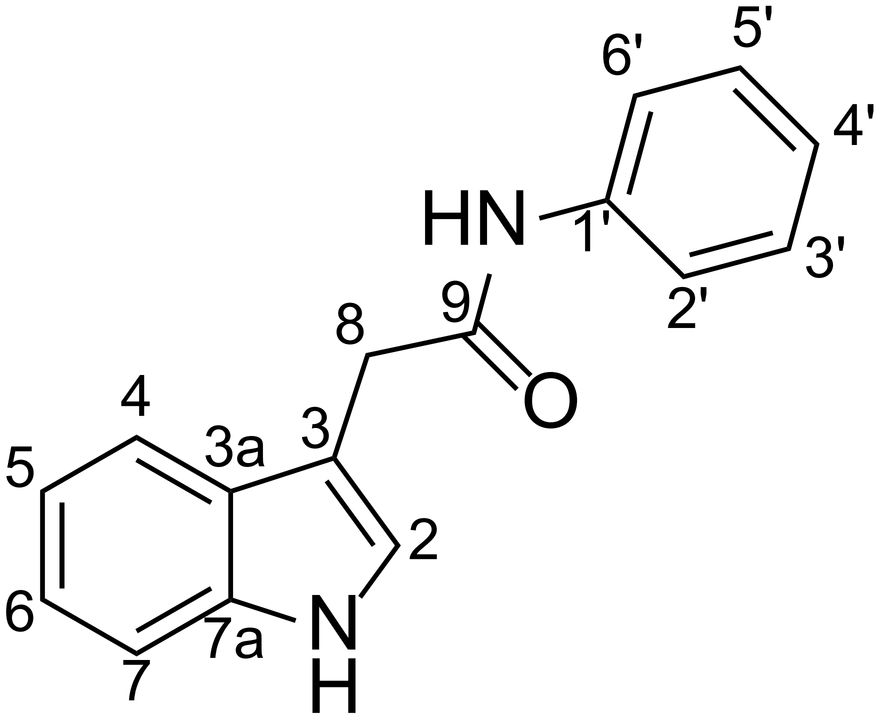

Figure S15. Atom numbering of lindolin D (3d).

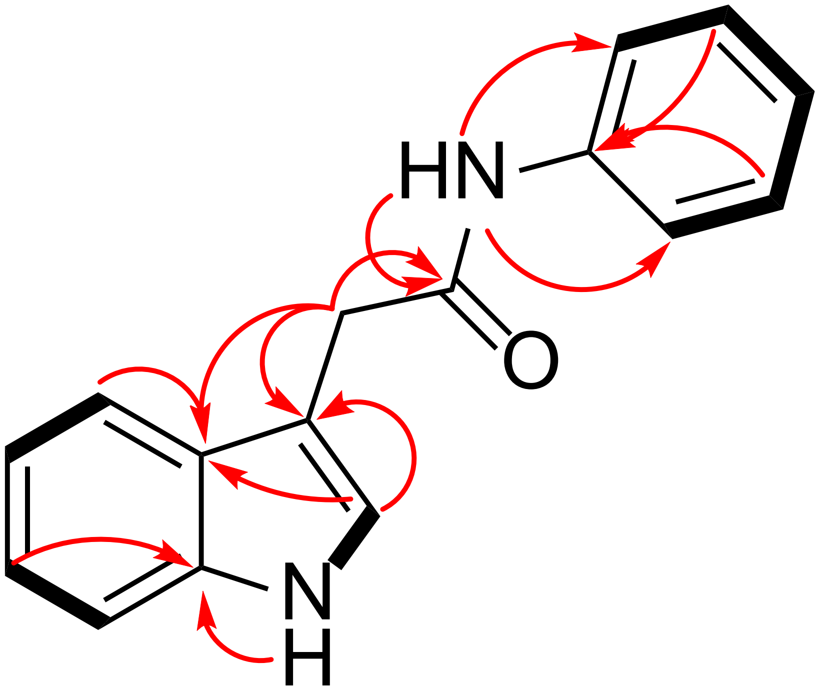

Figure S16. ^{1}H-^{1}H COSY (bold bonds) and ^{1}H-^{13}C HMBC (arrows) key correlations of lindolin D (3d).

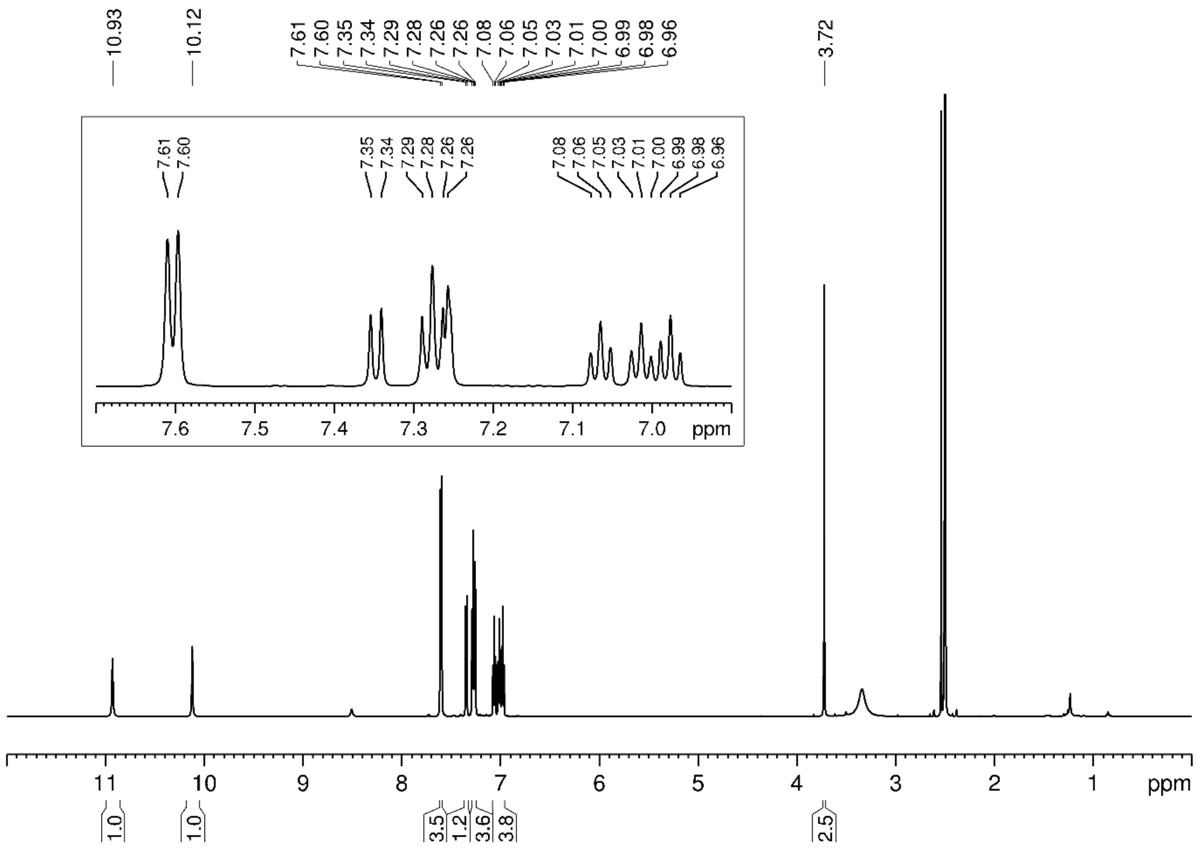

Figure S17. ^{1}H NMR spectrum of lindolin D (3d) in DMSO-d_6 recorded at 600 MHz.

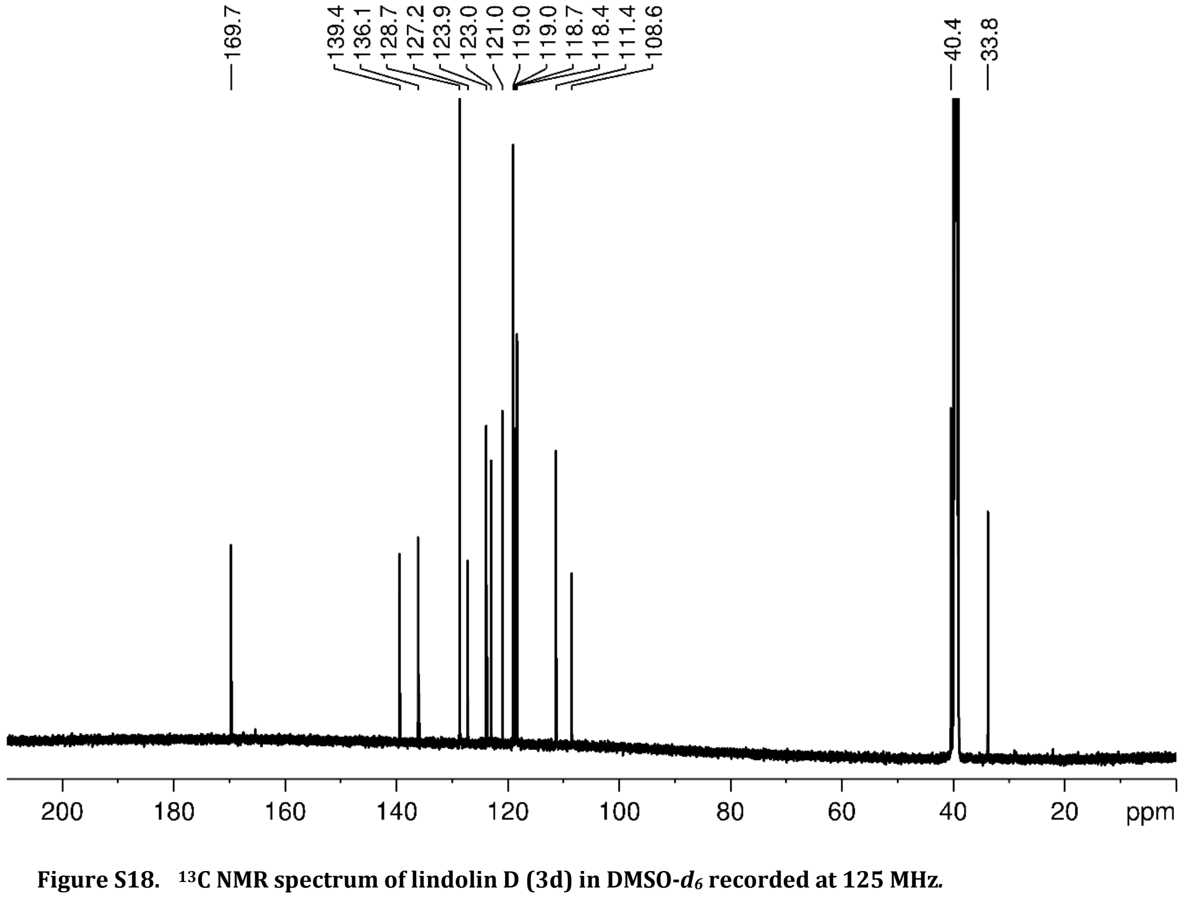

Figure S18. ^{13}C NMR spectrum of lindolin D (3d) in DMSO-d_6 recorded at 125 MHz.

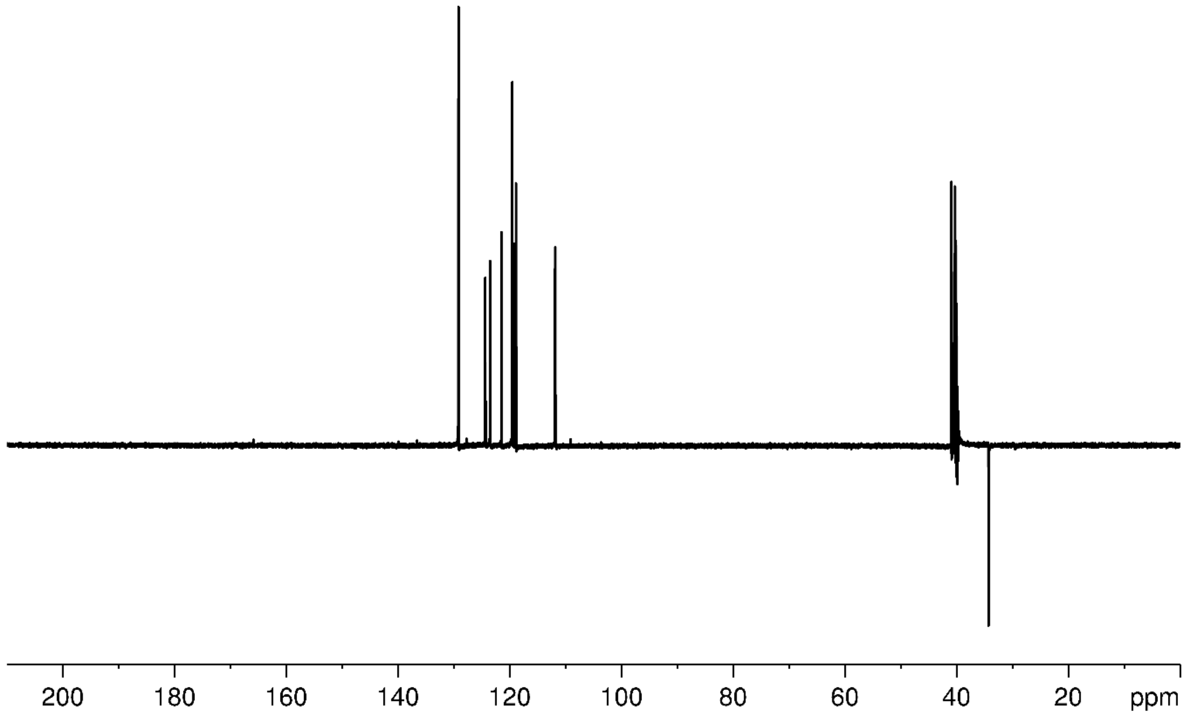

Figure S19. DEPT-135 NMR spectrum of lindolin D (3d) in DMSO-d_6.

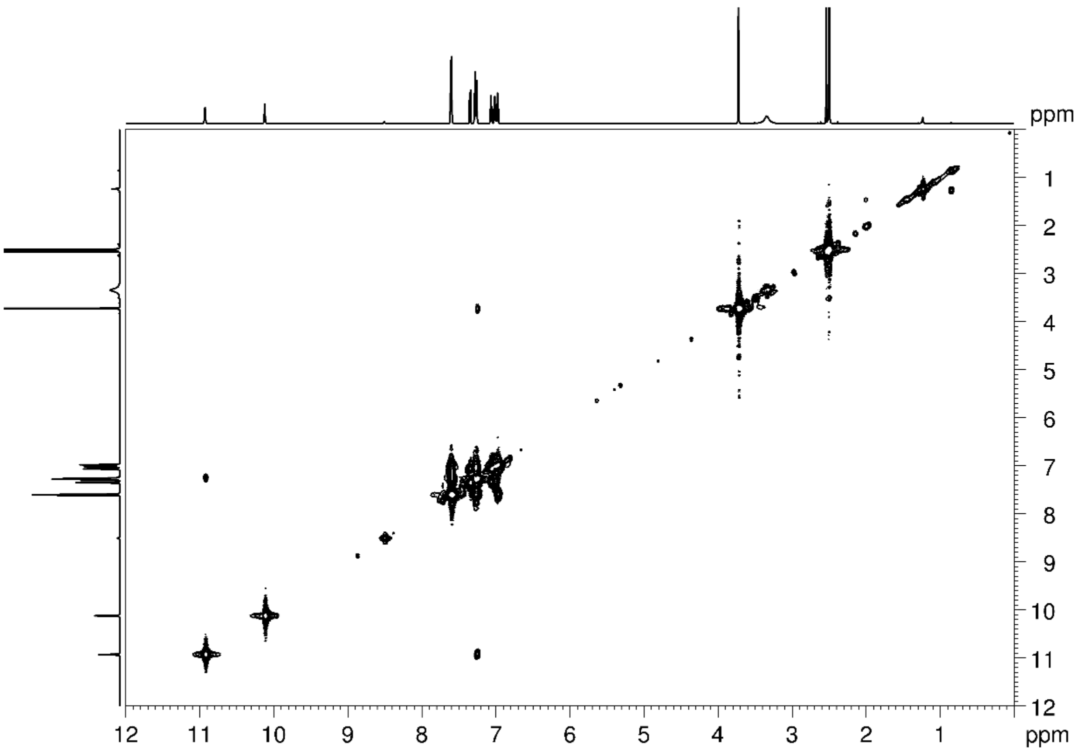

Figure S20. ^{1}H-^{1}H COSY NMR spectrum of lindolin D (3d) in DMSO-d_6.

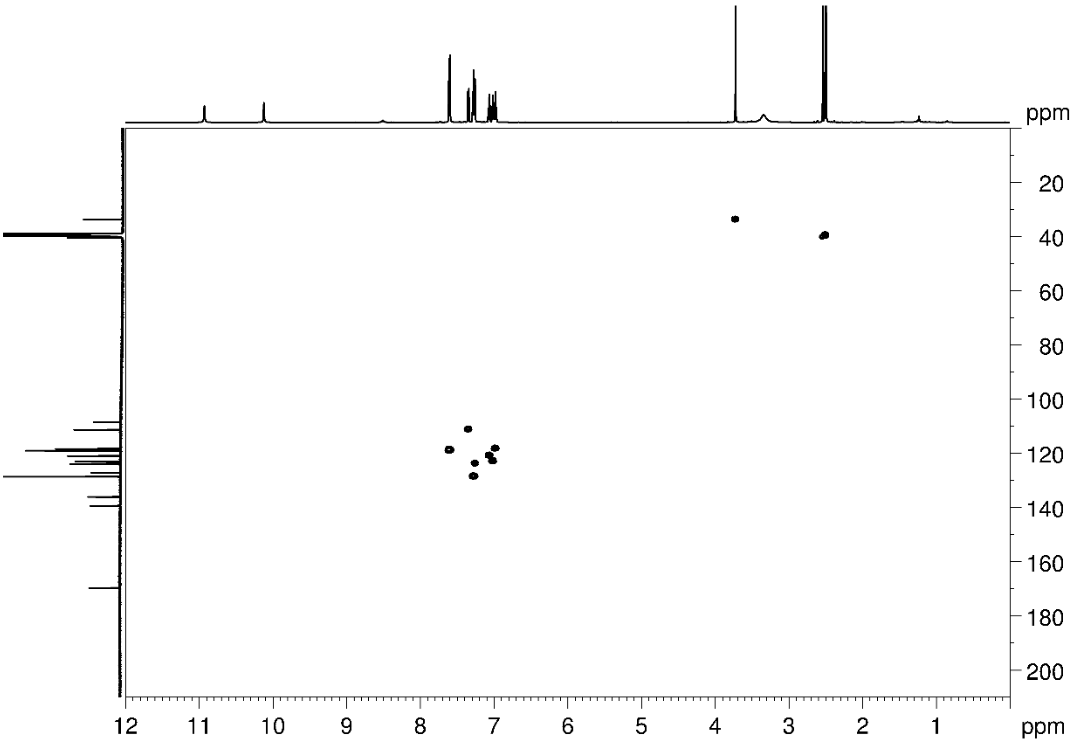

Figure S21. ¹H-¹³C HSQC NMR spectrum of lindolin D (3d) in DMSO-d_6.

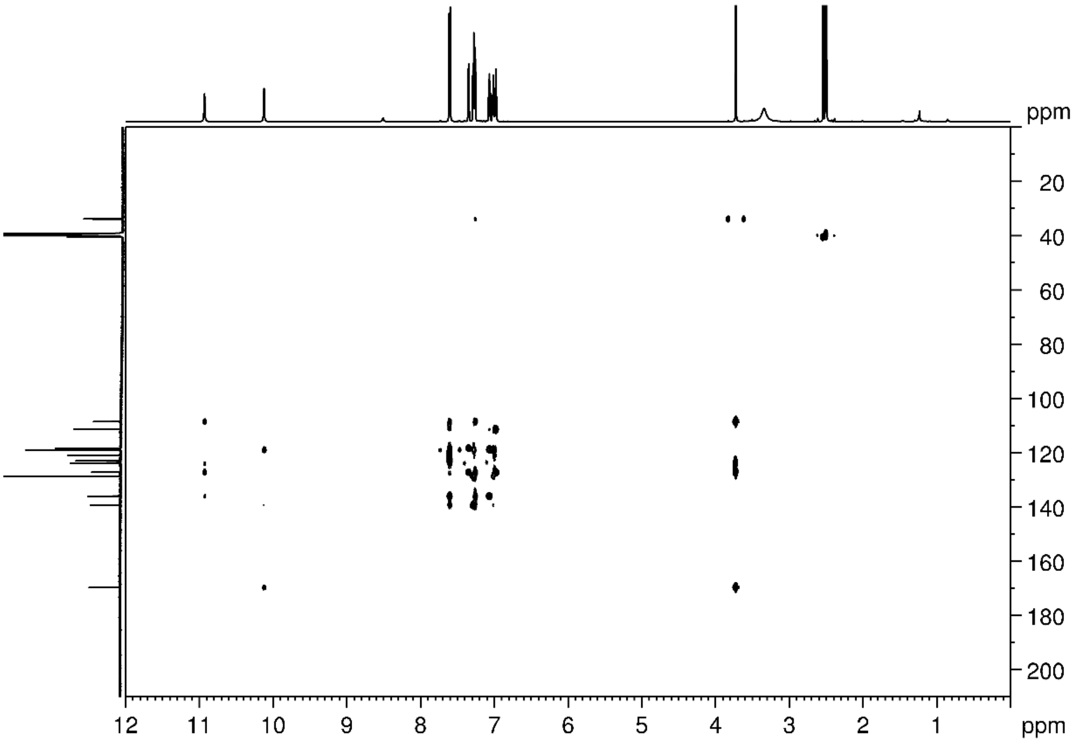

Figure S22. ¹H-¹³C HMBC NMR spectrum of lindolin D (3d) in DMSO-d_6.

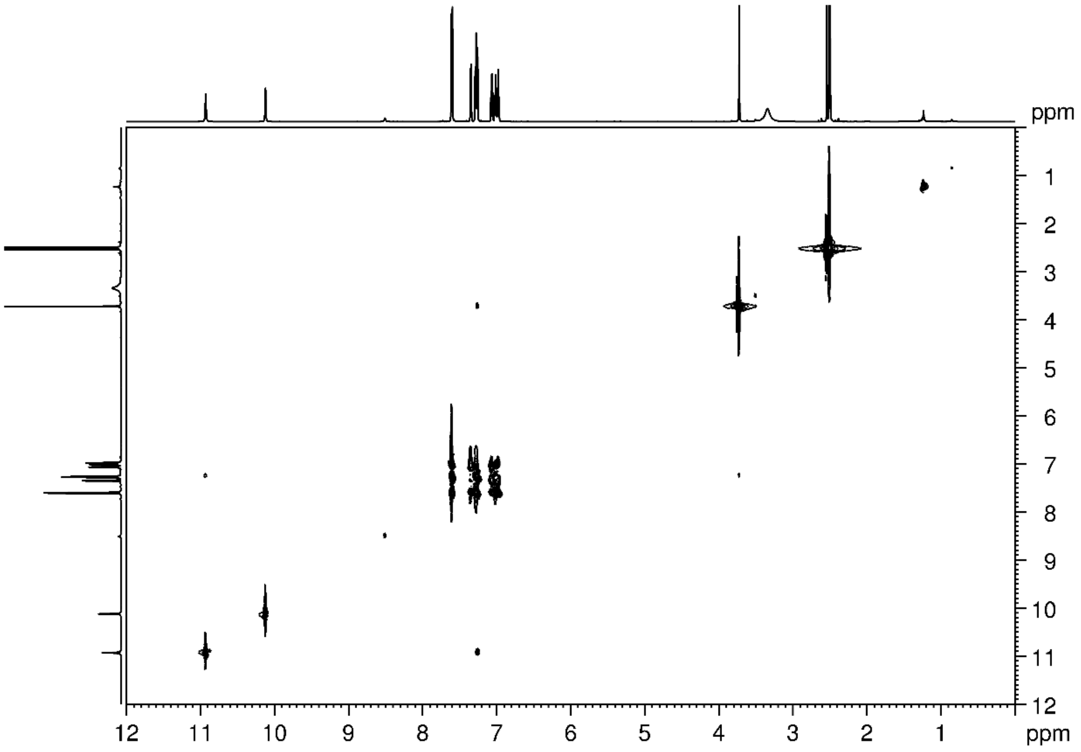

Figure S23. ^{1}H-^{1}H TOCSY NMR spectrum of lindolin D (3d) in DMSO-*d*$_{6}$.

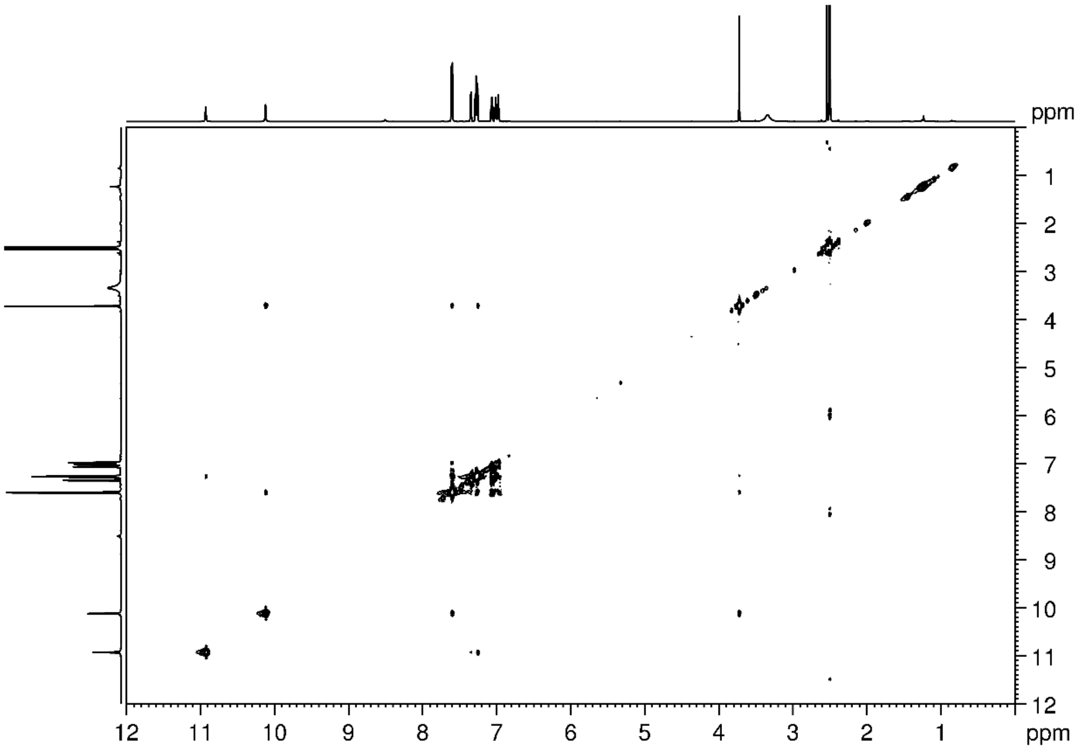

Figure S24. ^{1}H-^{1}H ROESY NMR spectrum of lindolin D (3d) in DMSO-*d*$_{6}$.

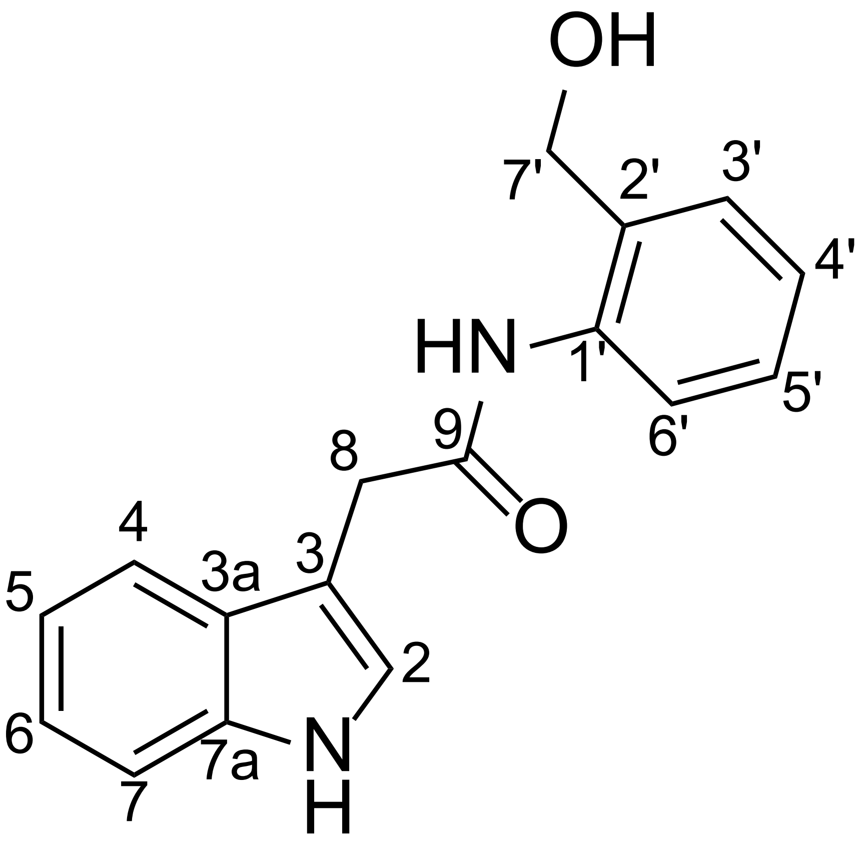

Figure S25. Atom numbering of lindolin E (3e).

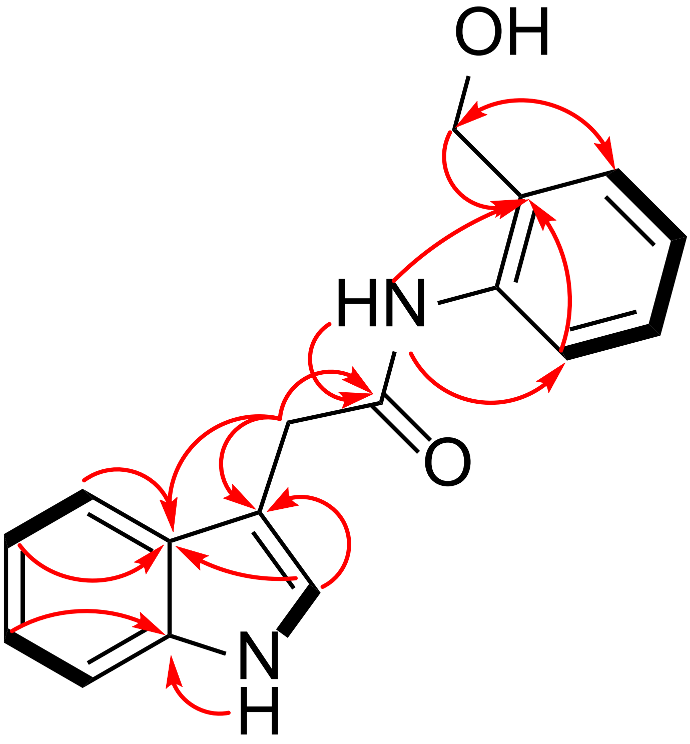

Figure S26. ^{1}H-^{1}H COSY (bold bonds) and ^{1}H-^{13}C HMBC (arrows) key correlations of lindolin E (3e).

.

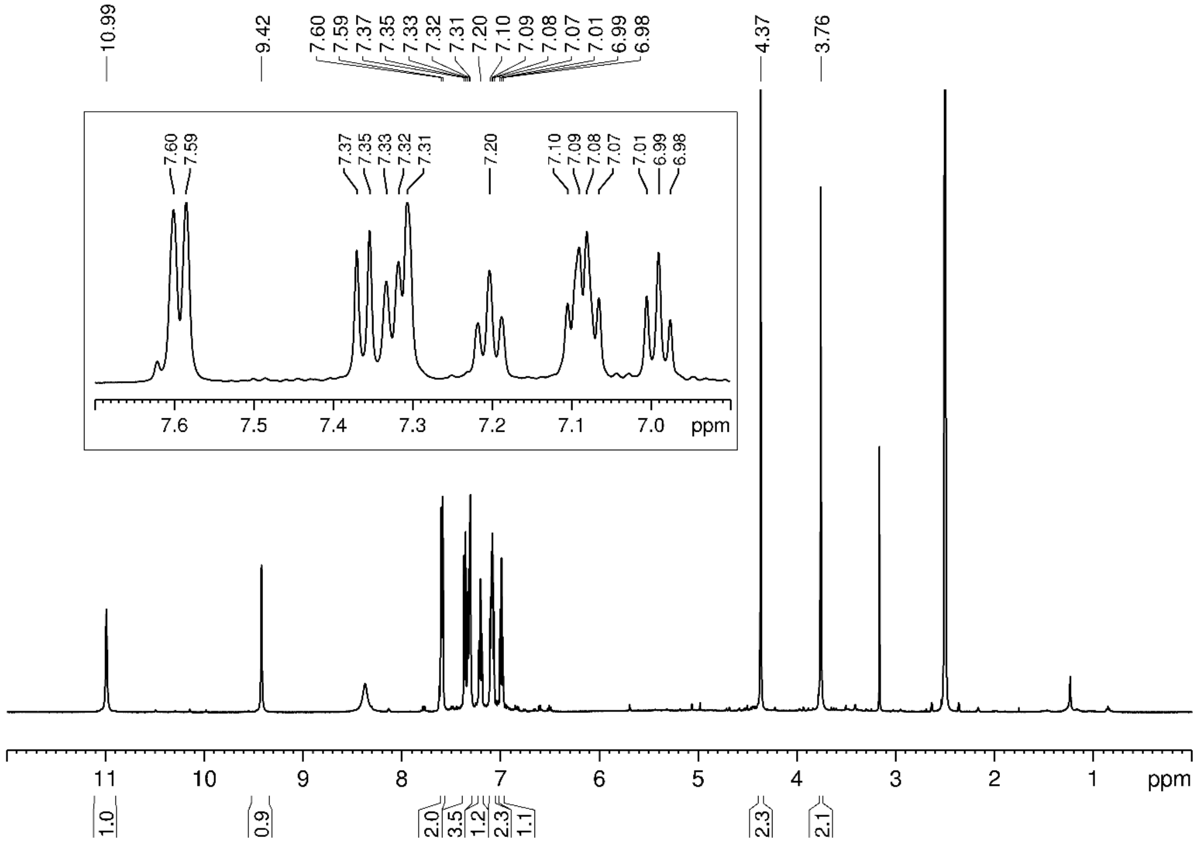

Figure S27. ^{1}H **NMR spectrum of lindolin E (3e) in DMSO-*d*$_6$ recorded at 500 MHz.**

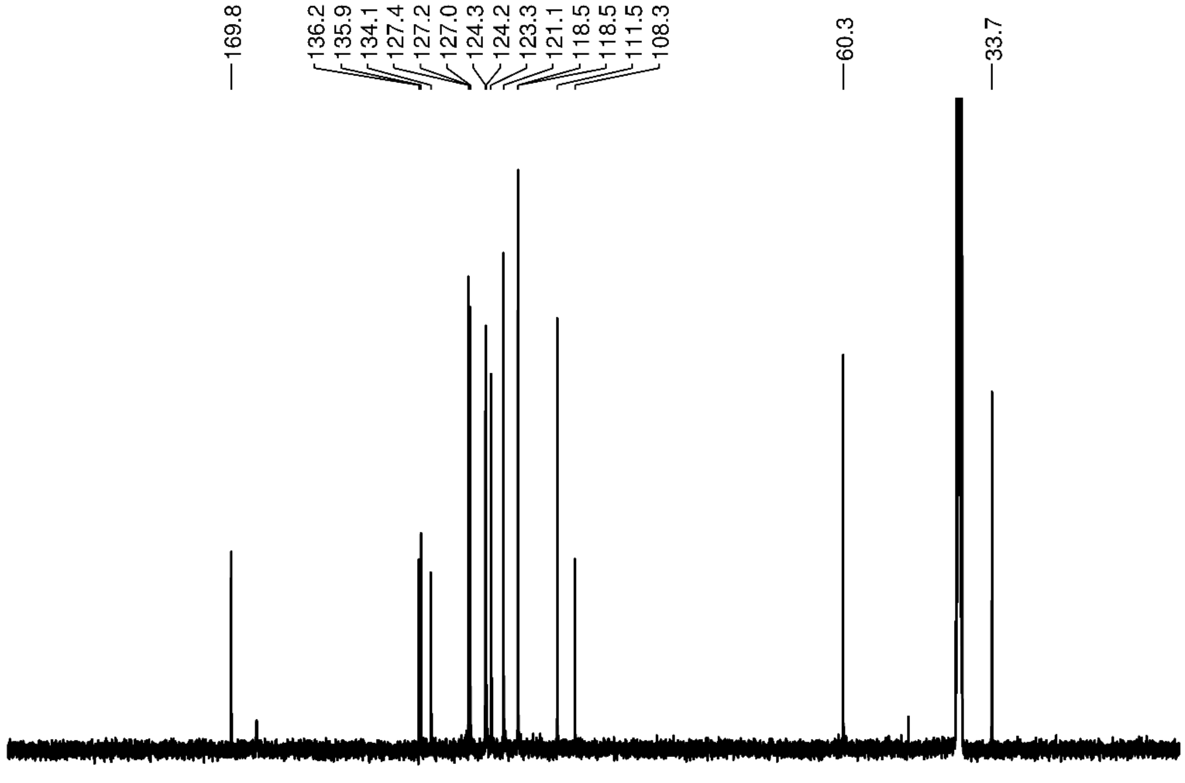

Figure S28. ^{13}C **NMR spectrum of lindolin E (3e) in DMSO-*d*$_6$ recorded at 125 MHz.**

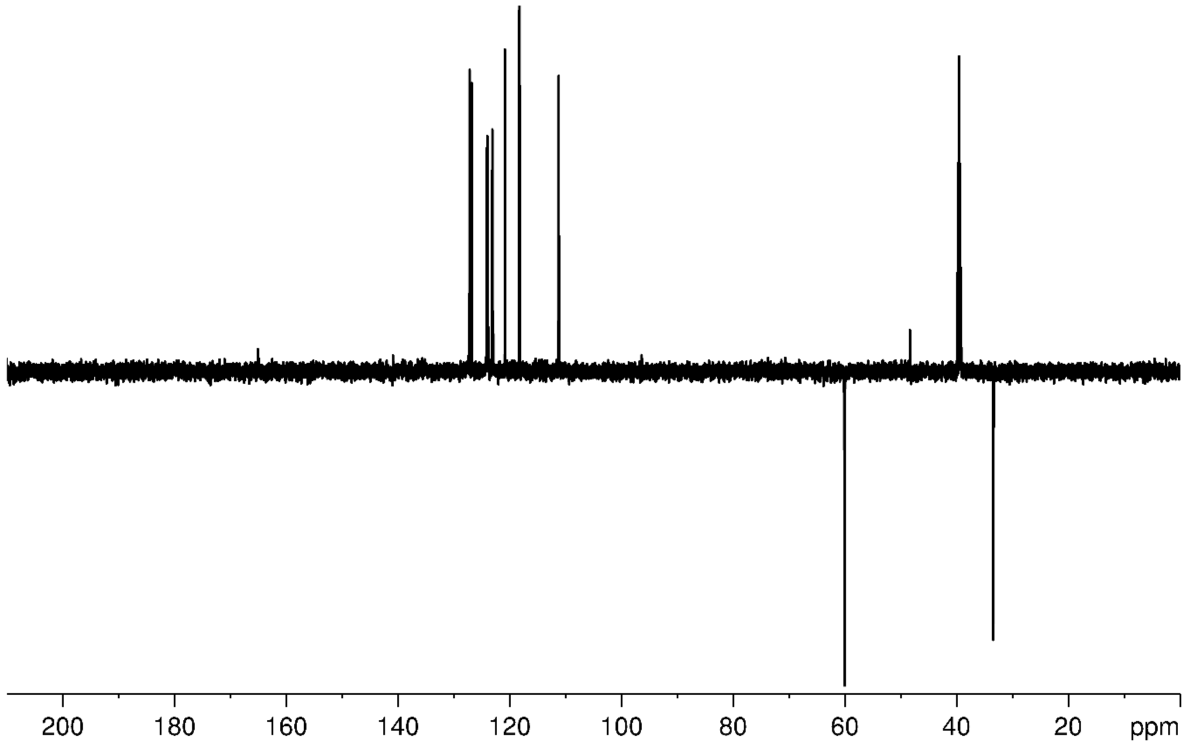

Figure S29. DEPT-135 NMR spectrum of lindolin E (3e) in DMSO-d_6.

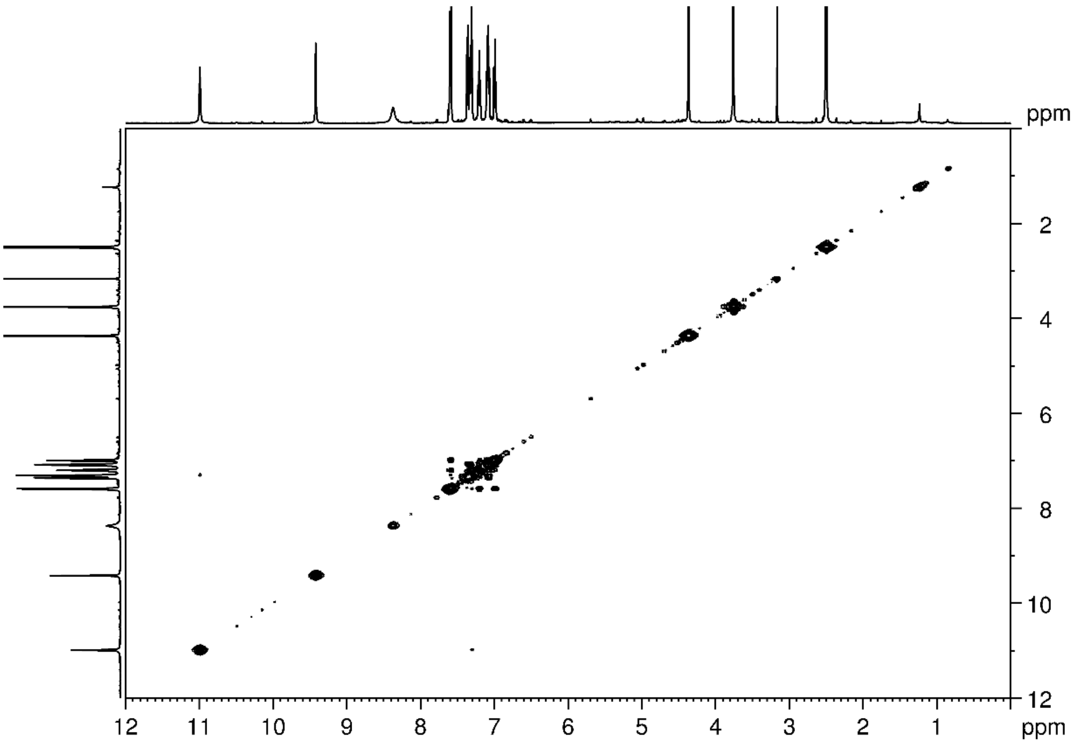

Figure S30. ^{1}H-^{1}H COSY NMR spectrum of lindolin E (3e) in DMSO-d_6.

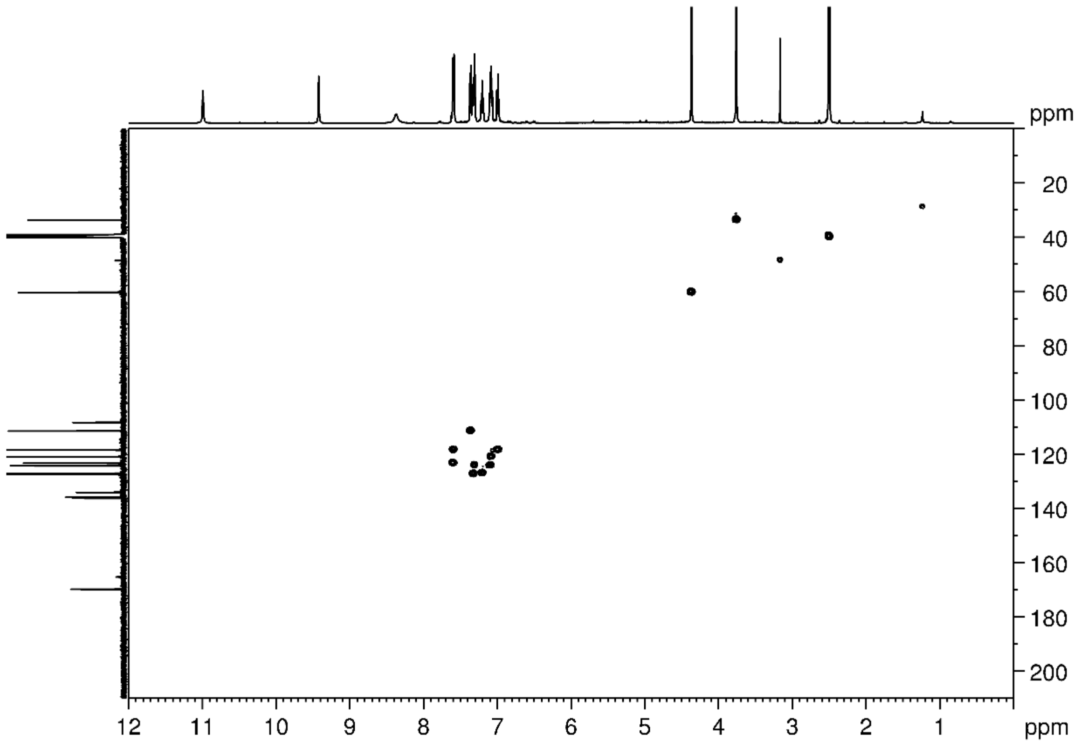

Figure S31. ¹H-¹³C HSQC NMR spectrum of lindolin E (3e) in DMSO-*d*₆.

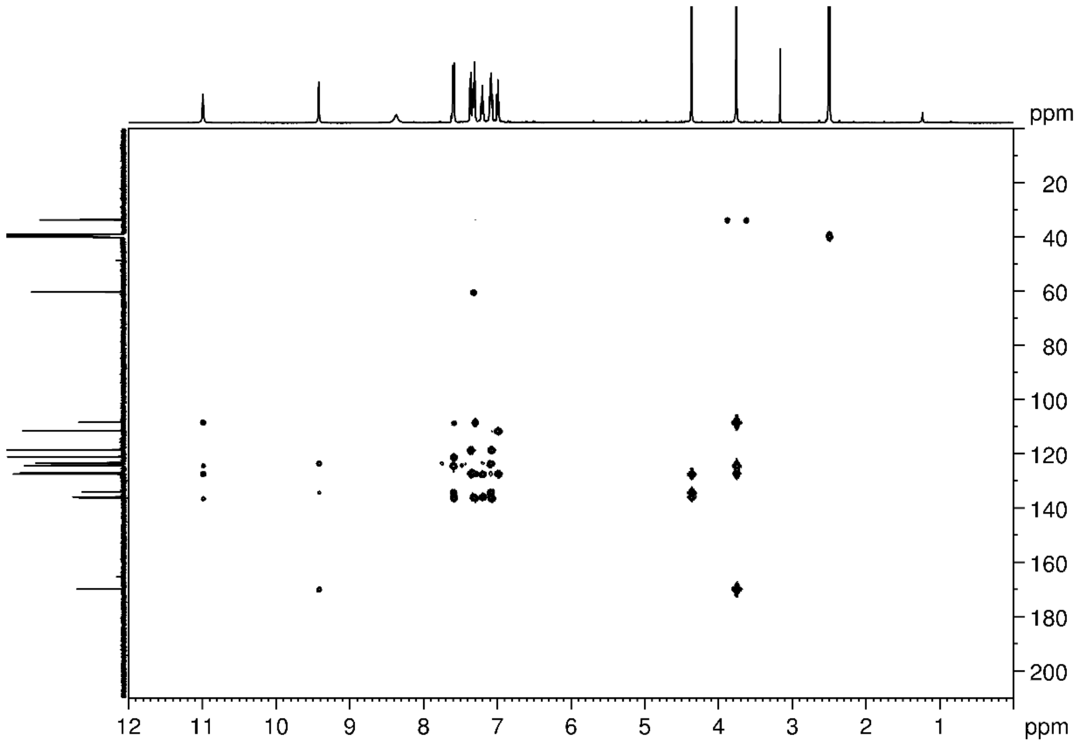

Figure S32. ¹H-¹³C HMBC NMR spectrum of lindolin E (3e) in DMSO-*d*₆.

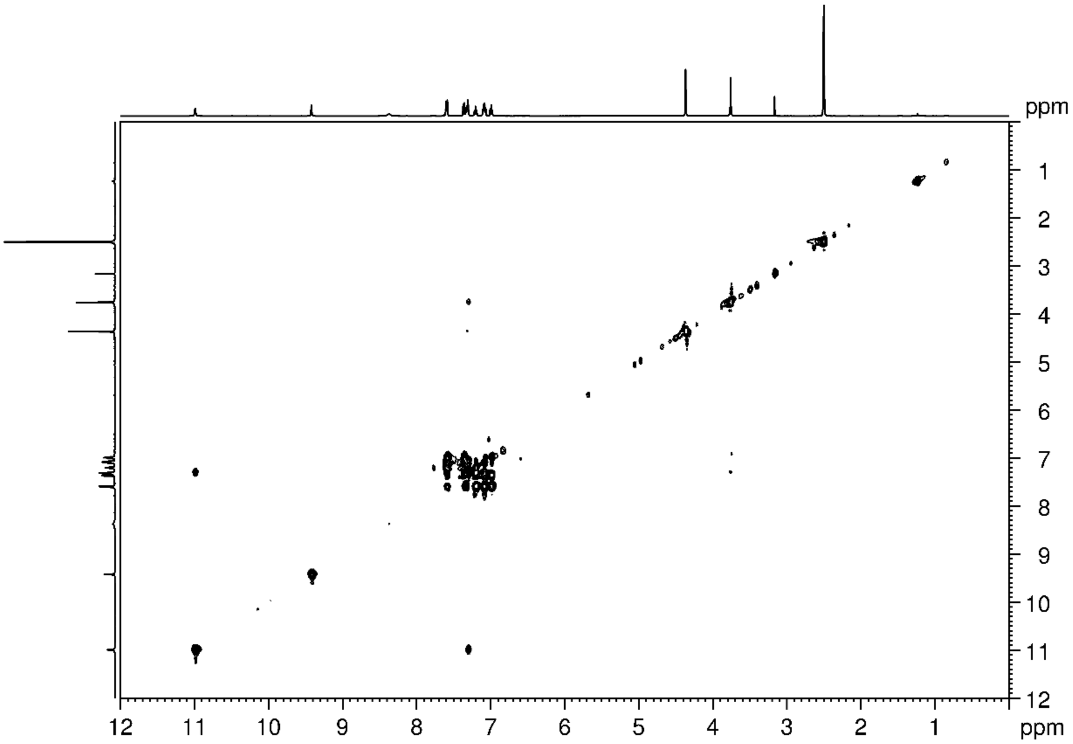

Figure S33. **^{1}H-^{1}H TOCSY NMR spectrum of lindolin E (3e) in DMSO-*d*$_6$.**

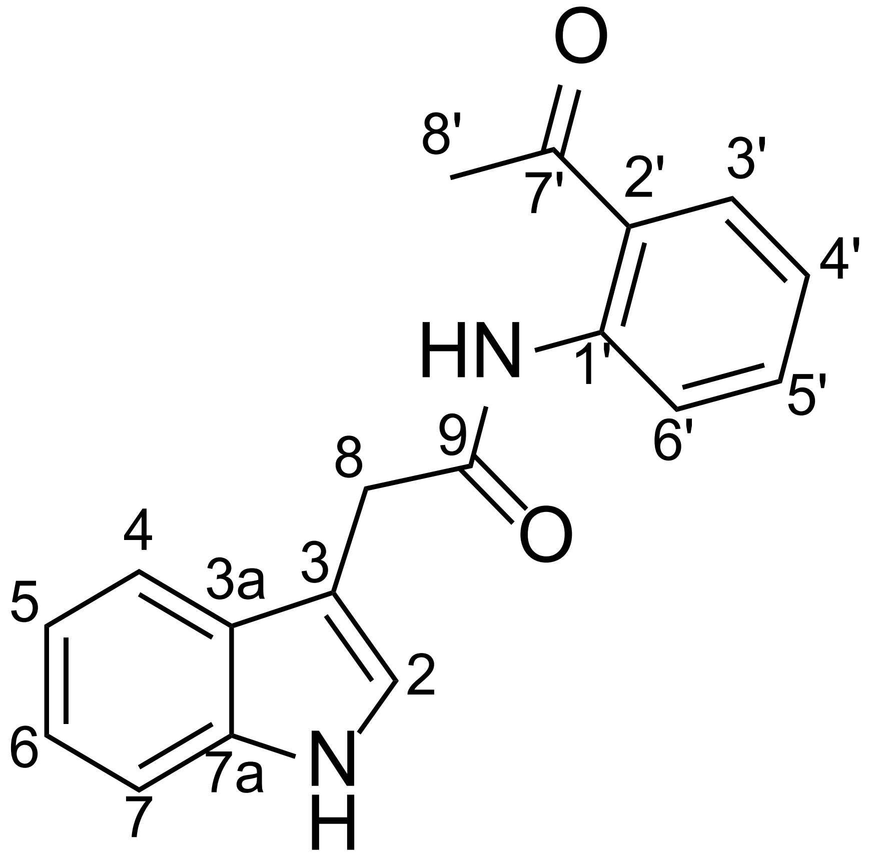

Figure S34. Atom numbering of lindolin F (3f).

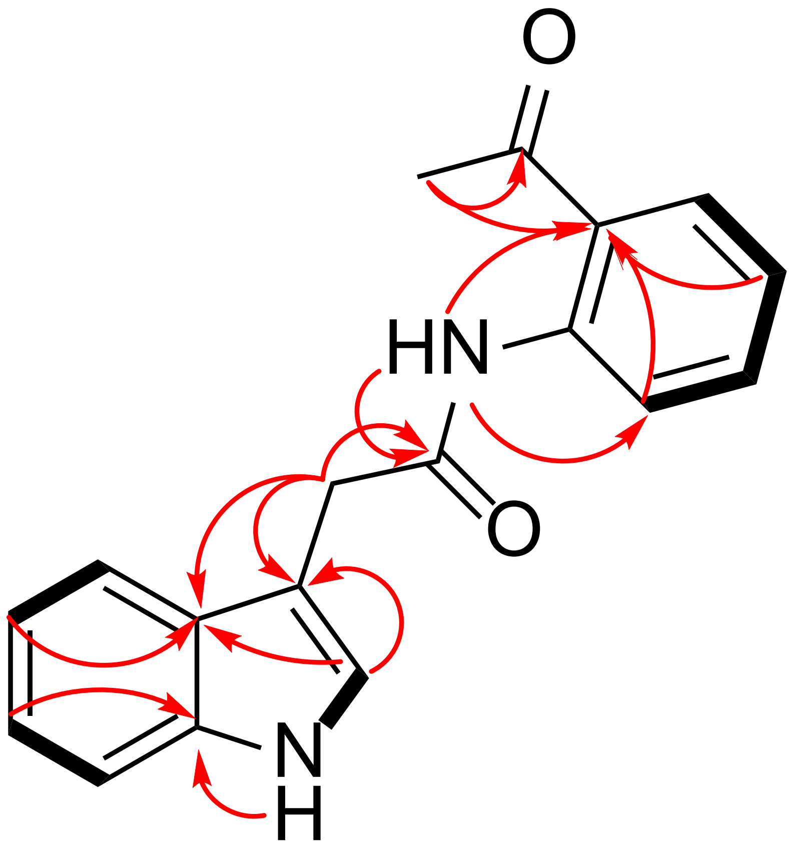

Figure S35. ^1H-^1H COSY (bold bonds) and ^1H-^{13}C HMBC (arrows) key correlations of lindolin F (3f).

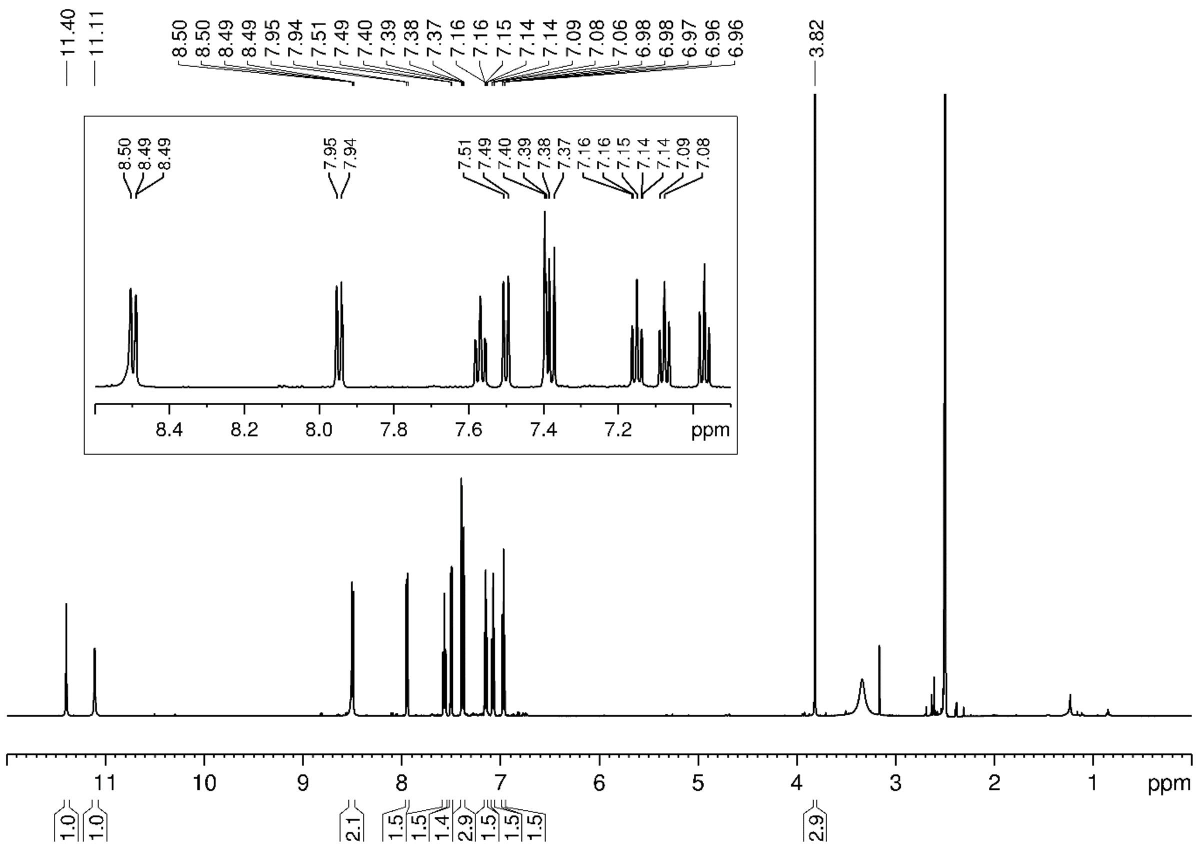

Figure S36. ^{1}H NMR spectrum of lindolin F (3f) in DMSO-d_6 recorded at 600 MHz.

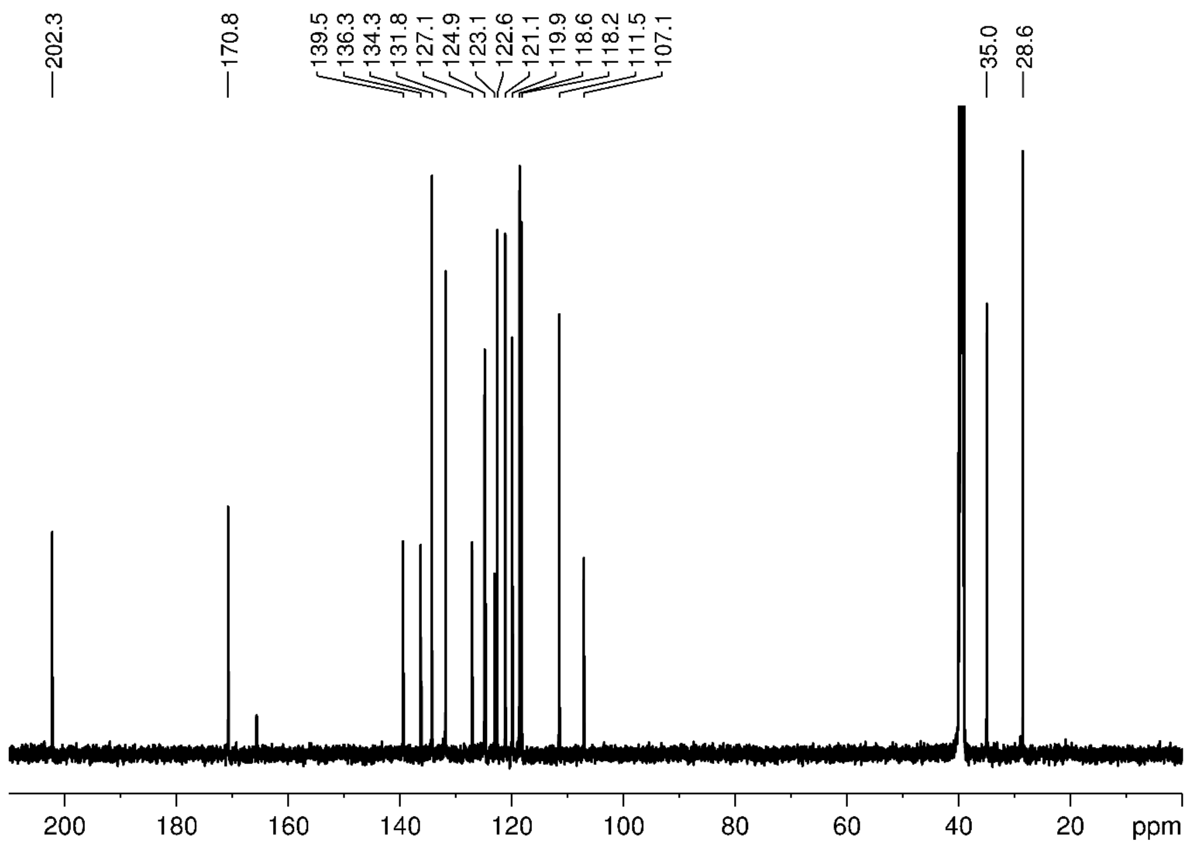

Figure S37. ^{13}C NMR spectrum of lindolin F (3f) in DMSO-d_6 recorded at 125 MHz.

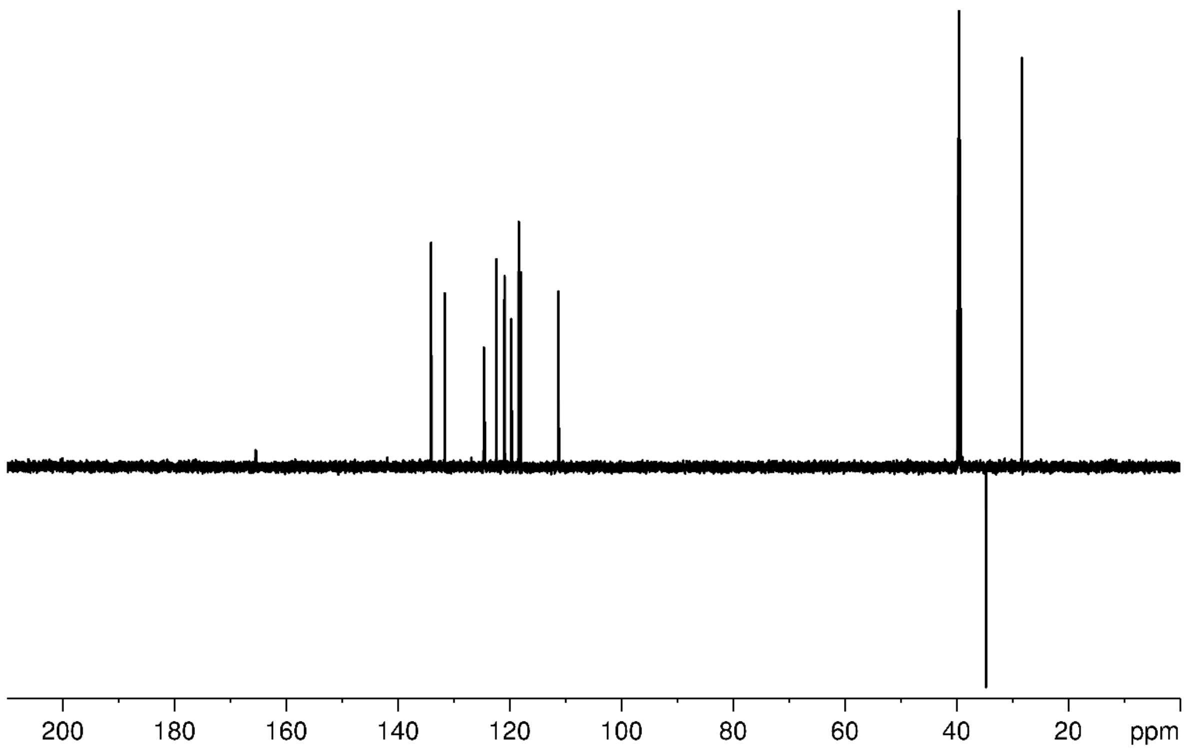

Figure S38. DEPT-135 NMR spectrum of lindolin F (3f) in DMSO-d_6.

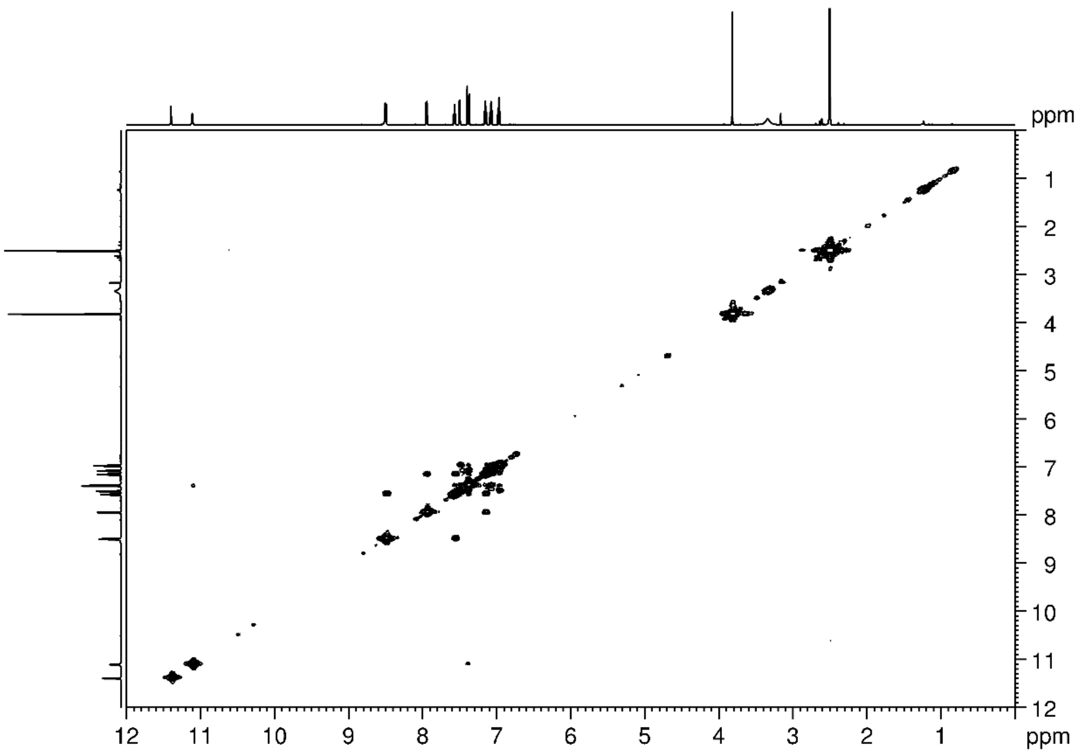

Figure S39. ^{1}H-^{1}H COSY NMR spectrum of lindolin F (3f) in DMSO-d_6.

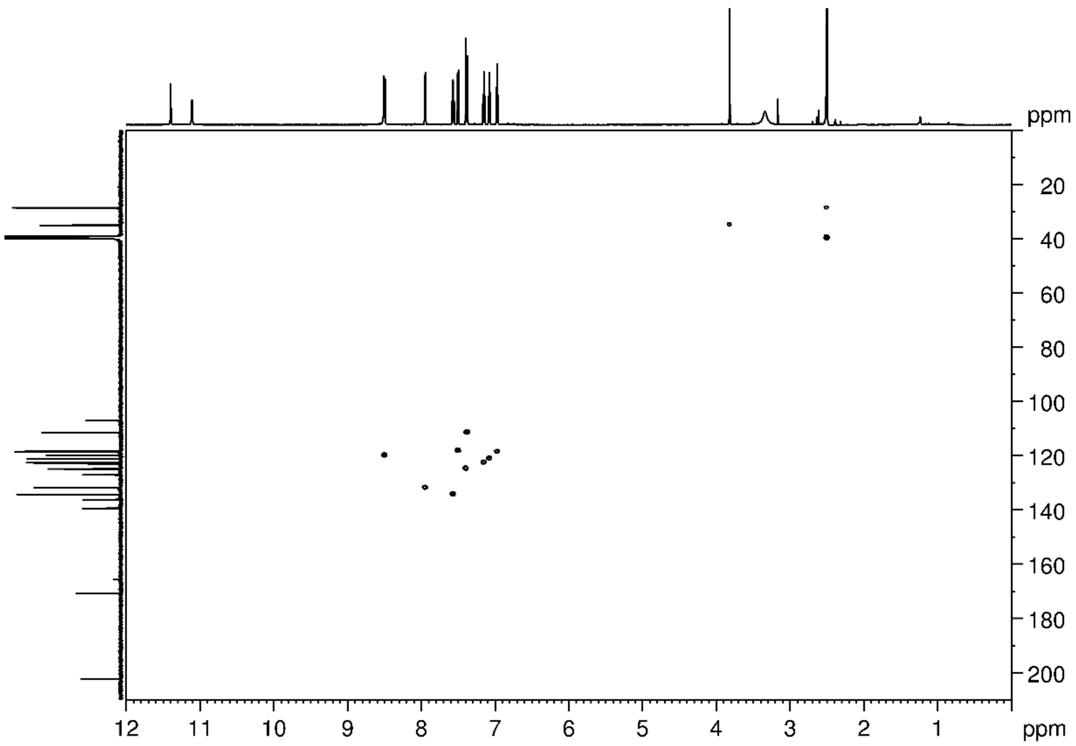

Figure S40. ^{1}H-^{13}C HSQC NMR spectrum of lindolin F (3f) in DMSO-d_6.

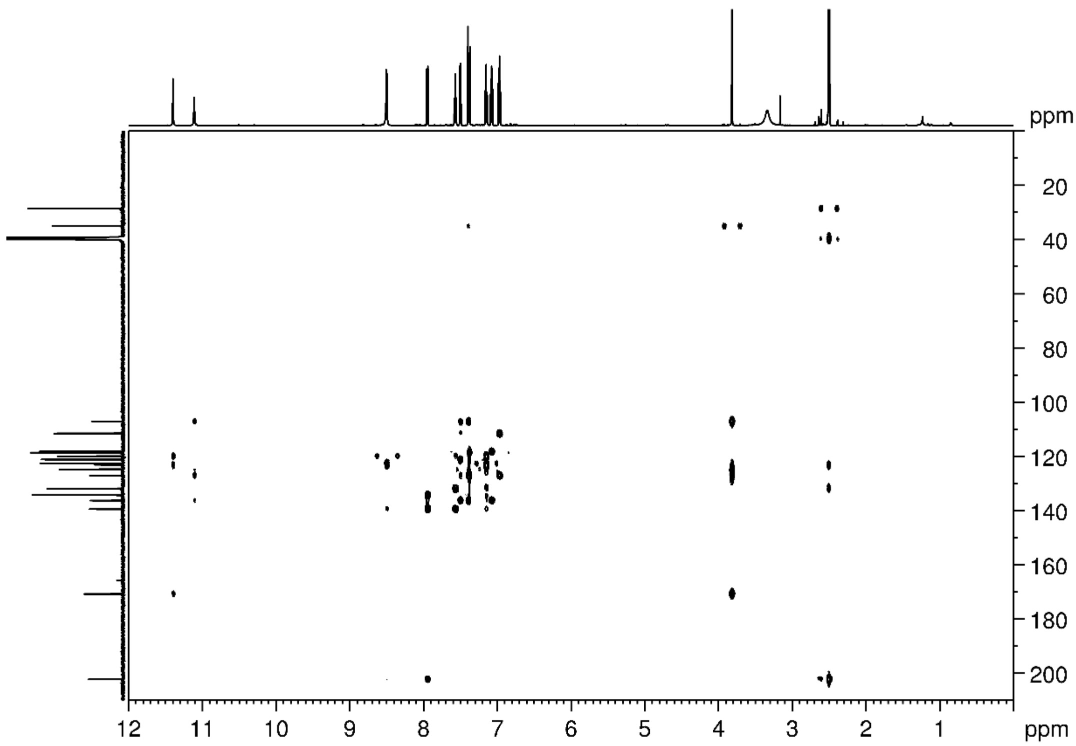

Figure S41. ^{1}H-^{13}C HMBC NMR spectrum of lindolin F (3f) in DMSO-d_6.

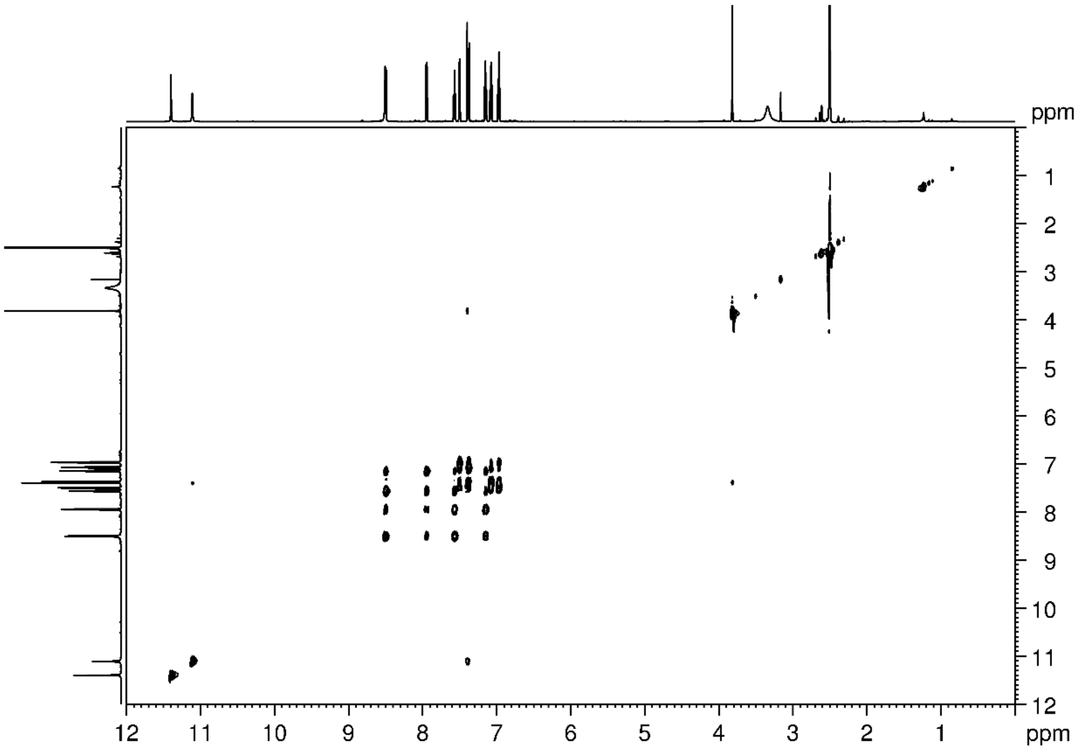

Figure S42. **^{1}H-^{1}H TOCSY NMR spectrum of lindolin F (3f) in DMSO-d_6.**

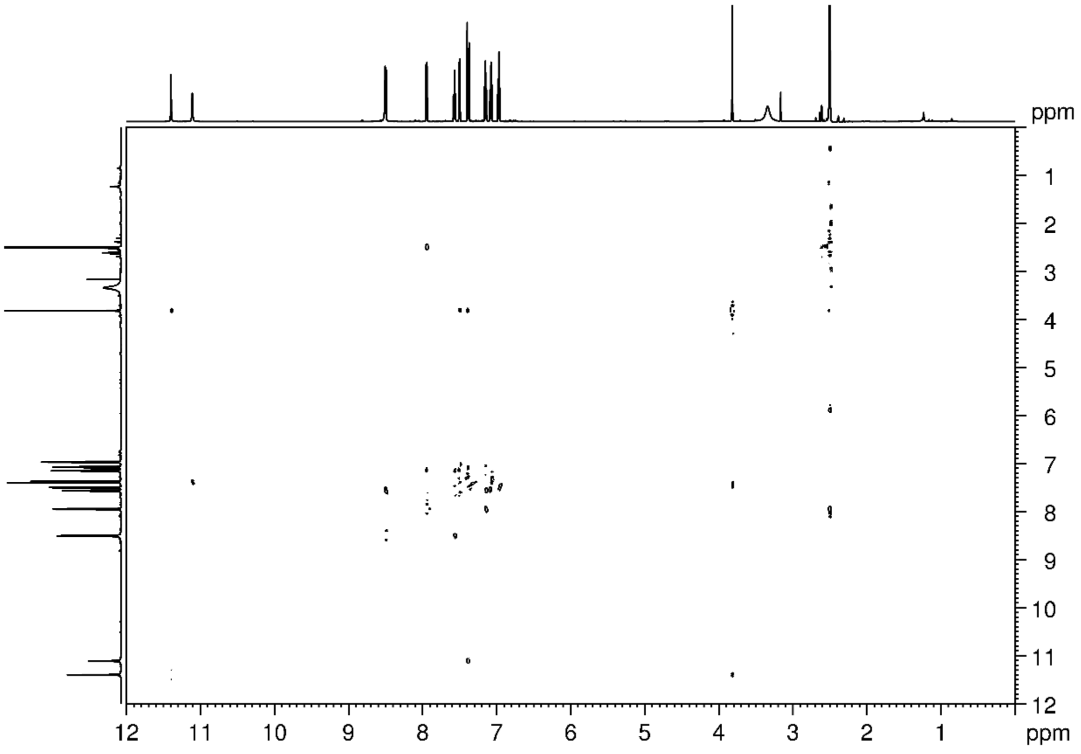

Figure S43. **^{1}H-^{1}H ROESY NMR spectrum of lindolin F (3f) in DMSO-d_6.**

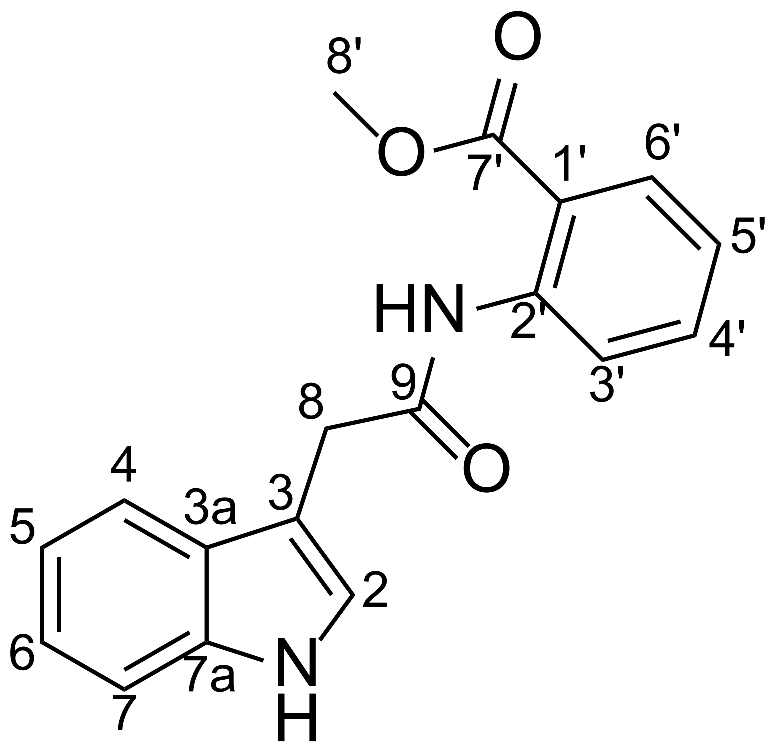

Figure S44. Atom numbering of lindolin G (3g).

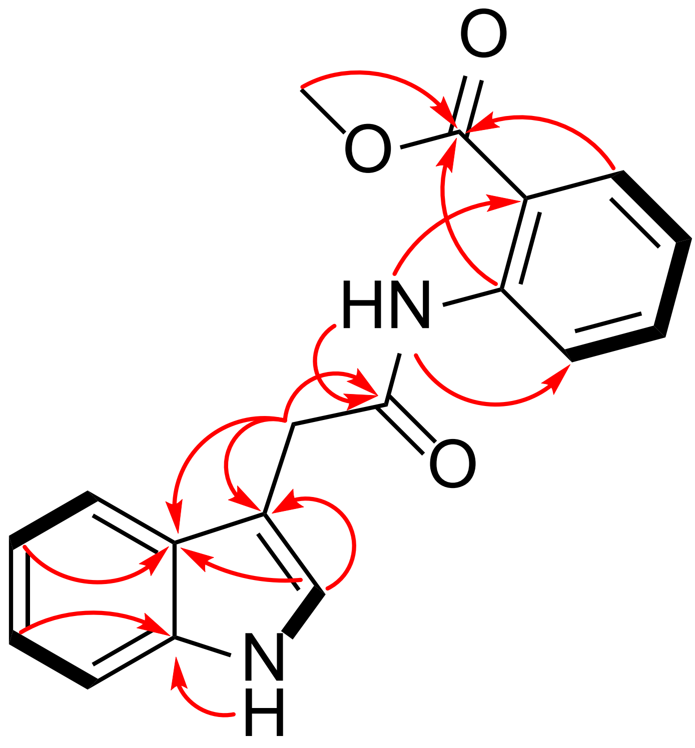

Figure S45. ^{1}H-^{1}H COSY (bold bonds) and ^{1}H-^{13}C HMBC (arrows) key correlations of lindolin G (3g).

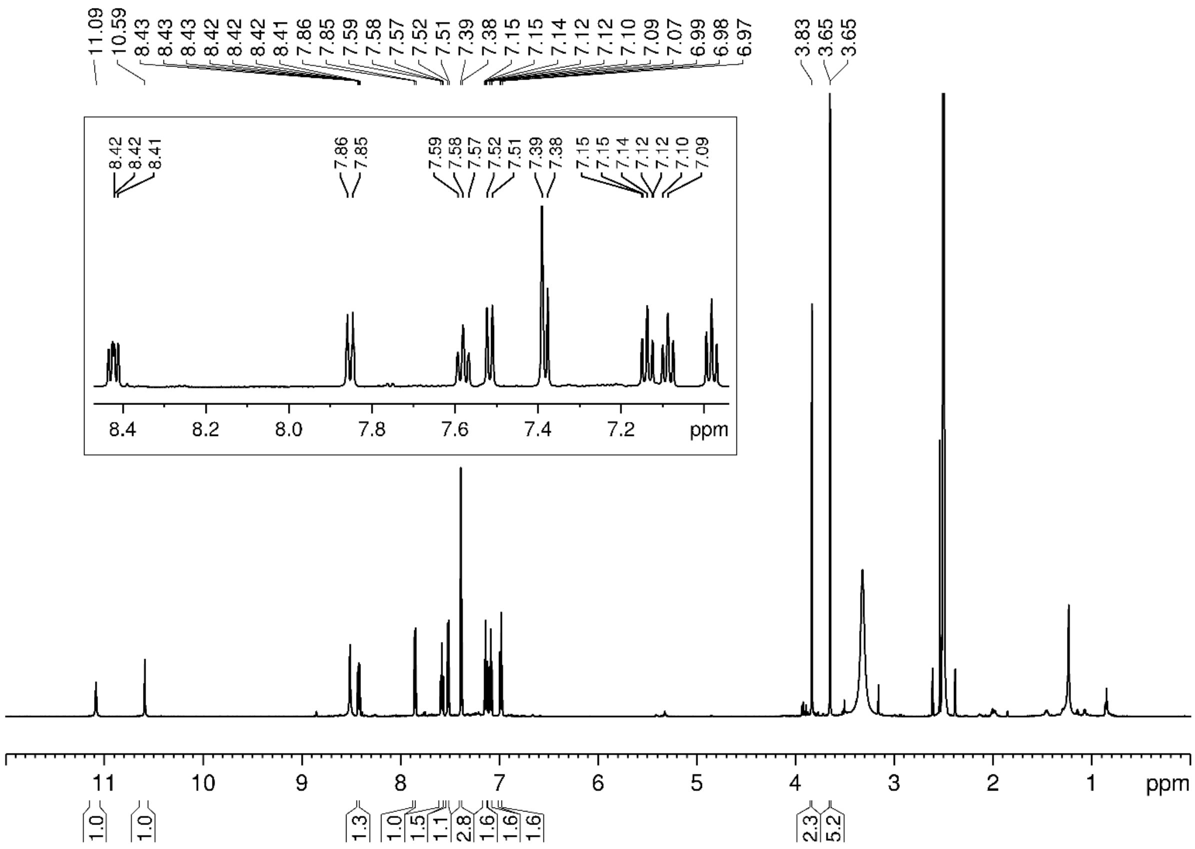

Figure S46. ^{1}H NMR spectrum of lindolin G (3g) in DMSO-d_6 recorded at 600 MHz.

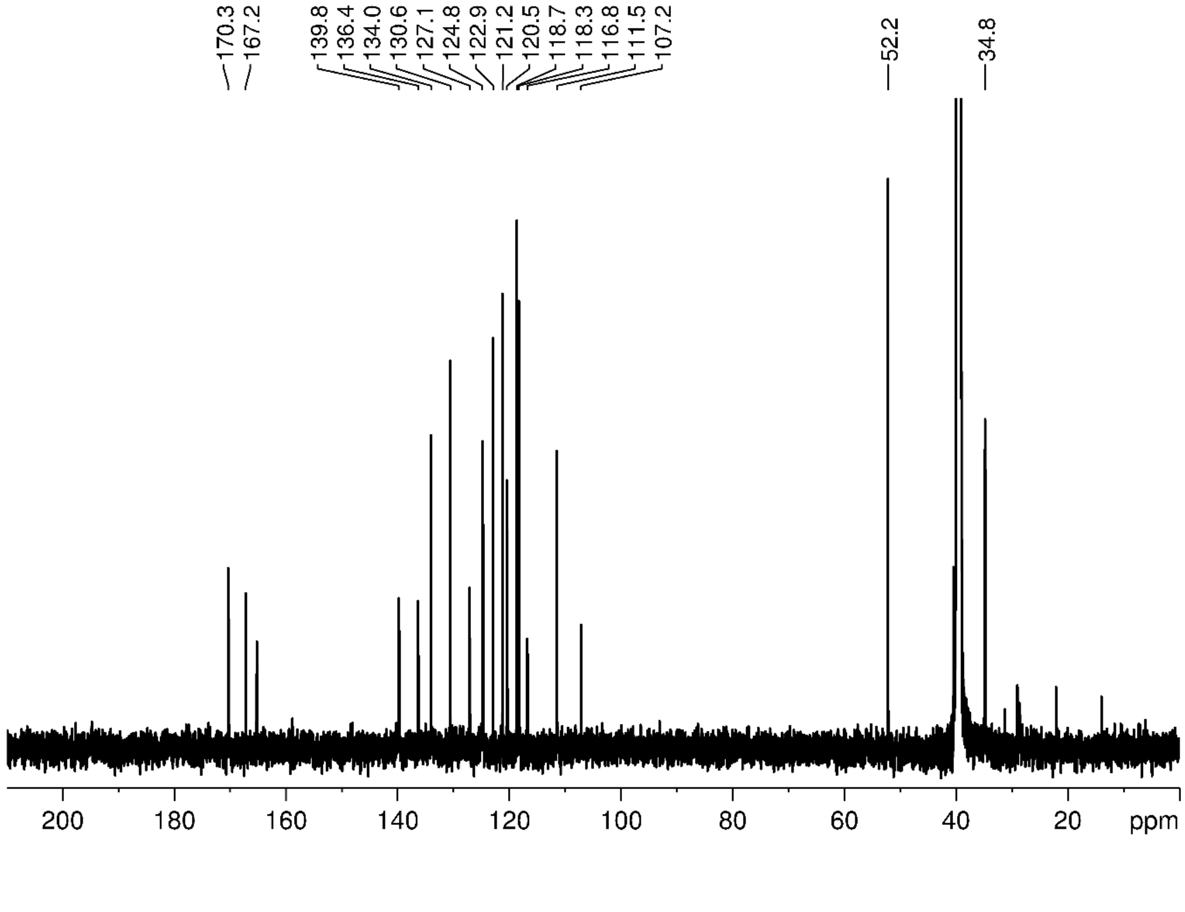

Figure S47. ^{13}C NMR spectrum of lindolin G (3g) in DMSO-d_6 recorded at 125 MHz.

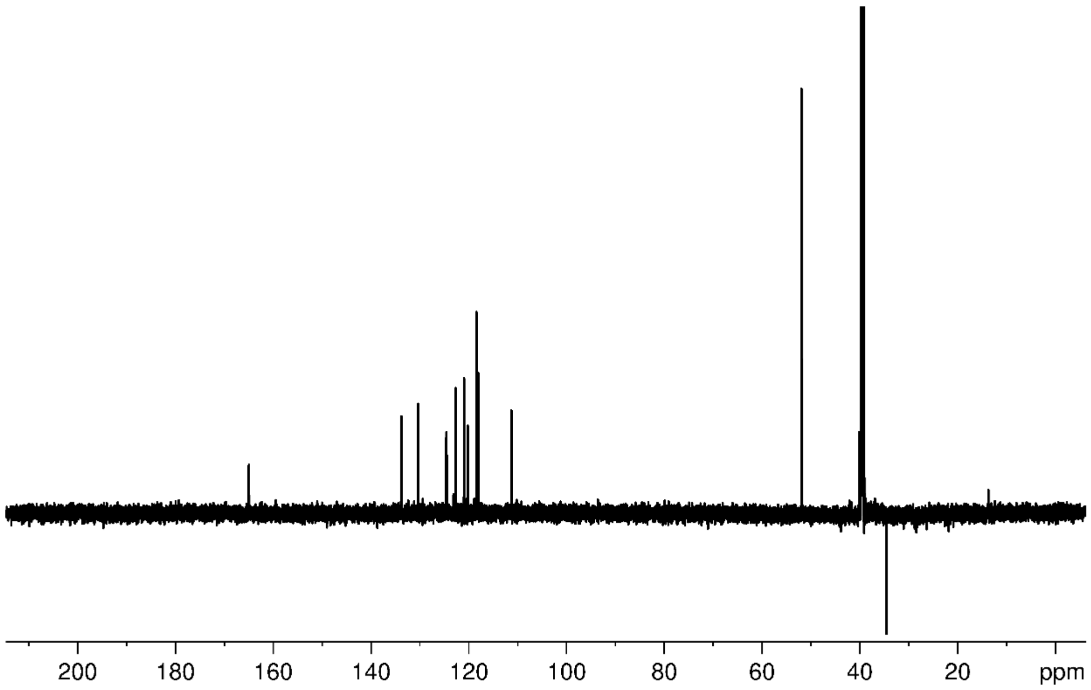

Figure S48. DEPT-135 NMR spectrum of lindolin G (3g) in DMSO-d_6.

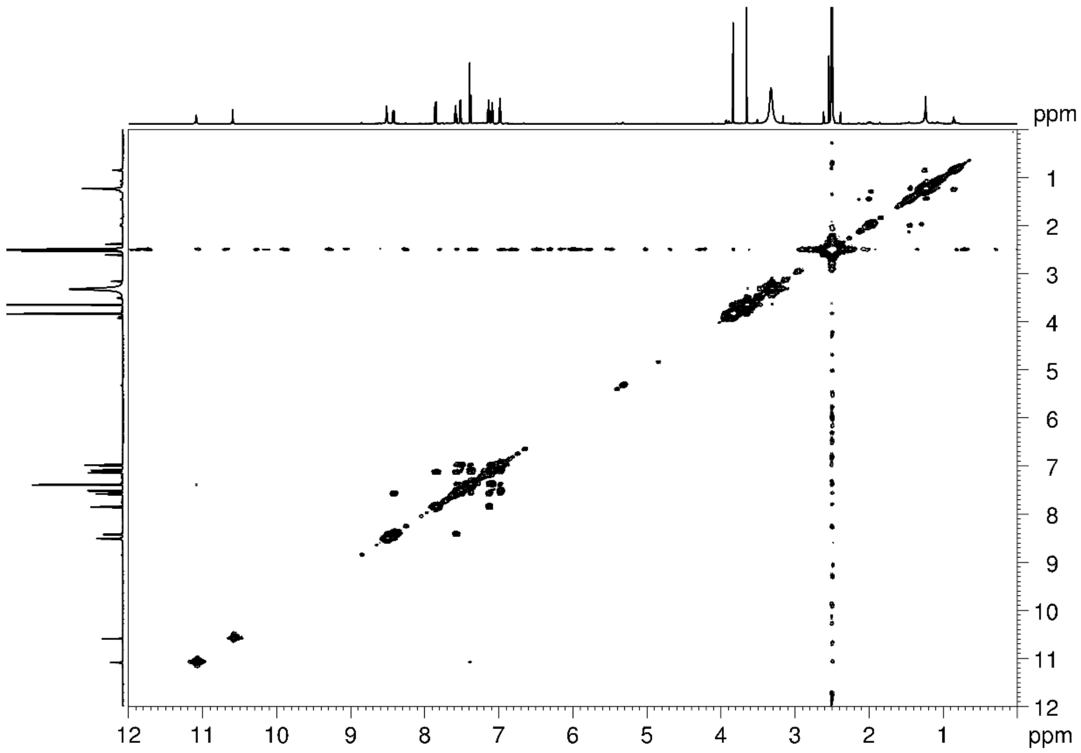

Figure S49. ^{1}H-^{1}H COSY NMR spectrum of lindolin G (3g) in DMSO-d_6.

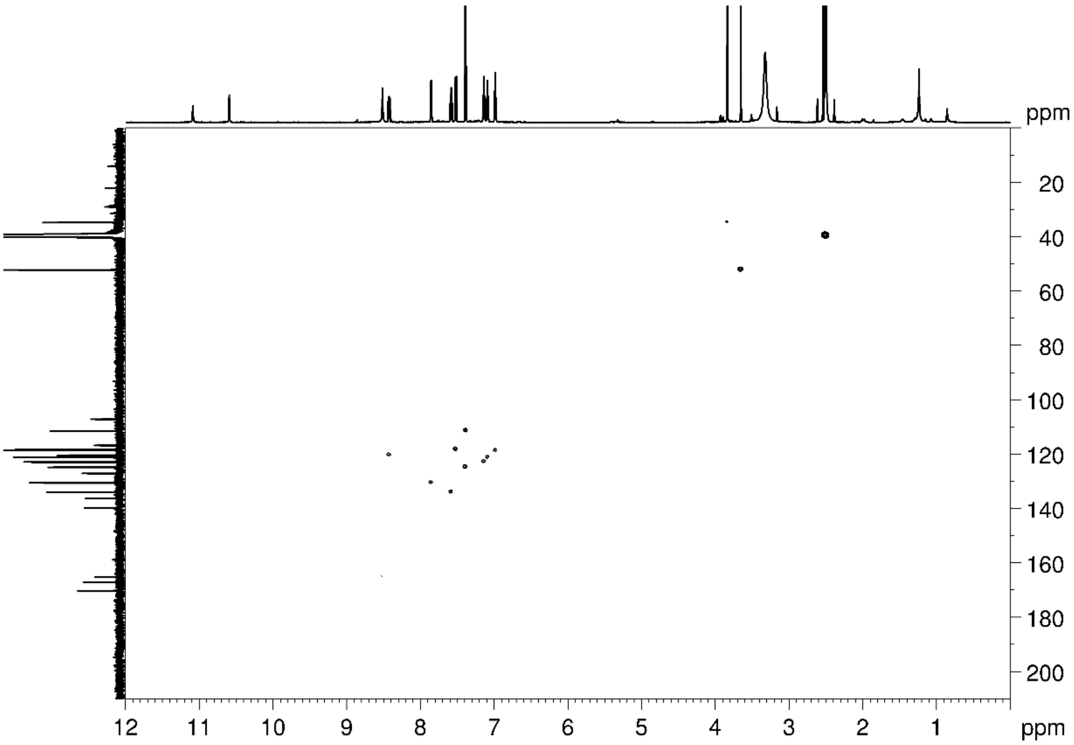

Figure S50. ^{1}H-^{13}C **HSQC NMR spectrum of lindolin G (3g) in DMSO-*d*$_6$.**

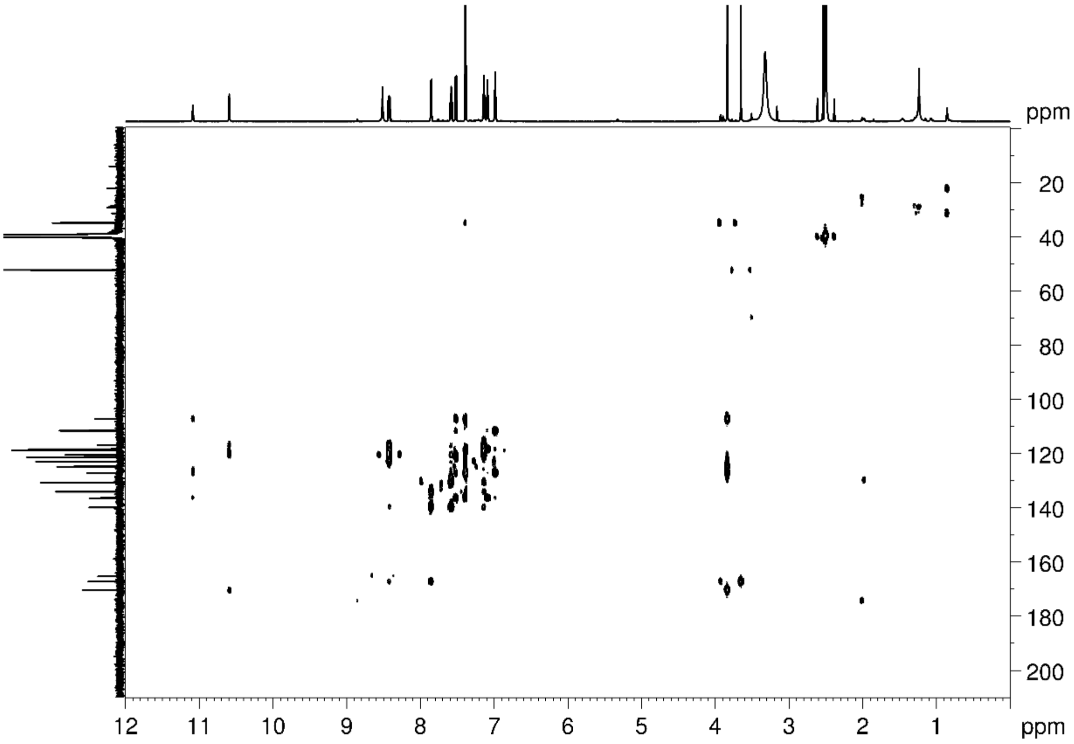

Figure S51. ^{1}H-^{13}C **HMBC NMR spectrum of lindolin G (3g) in DMSO-*d*$_6$.**

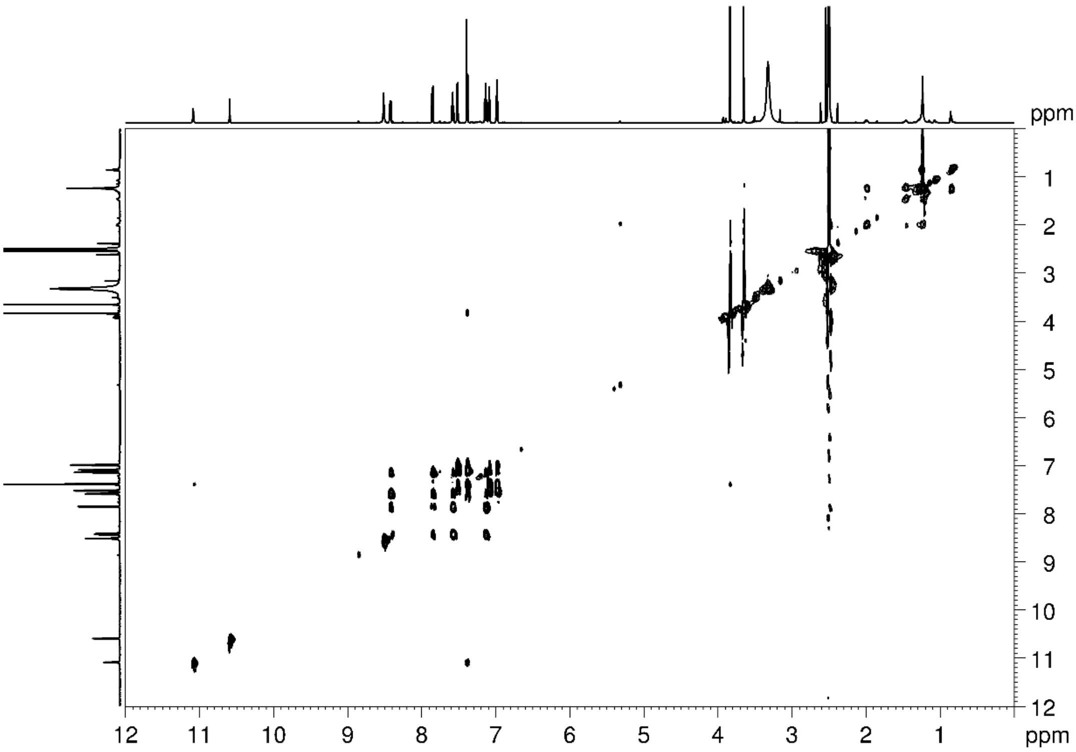

Figure S52. **^1H-^1H TOCSY NMR spectrum of lindolin G (3g) in DMSO-*d*$_6$.**

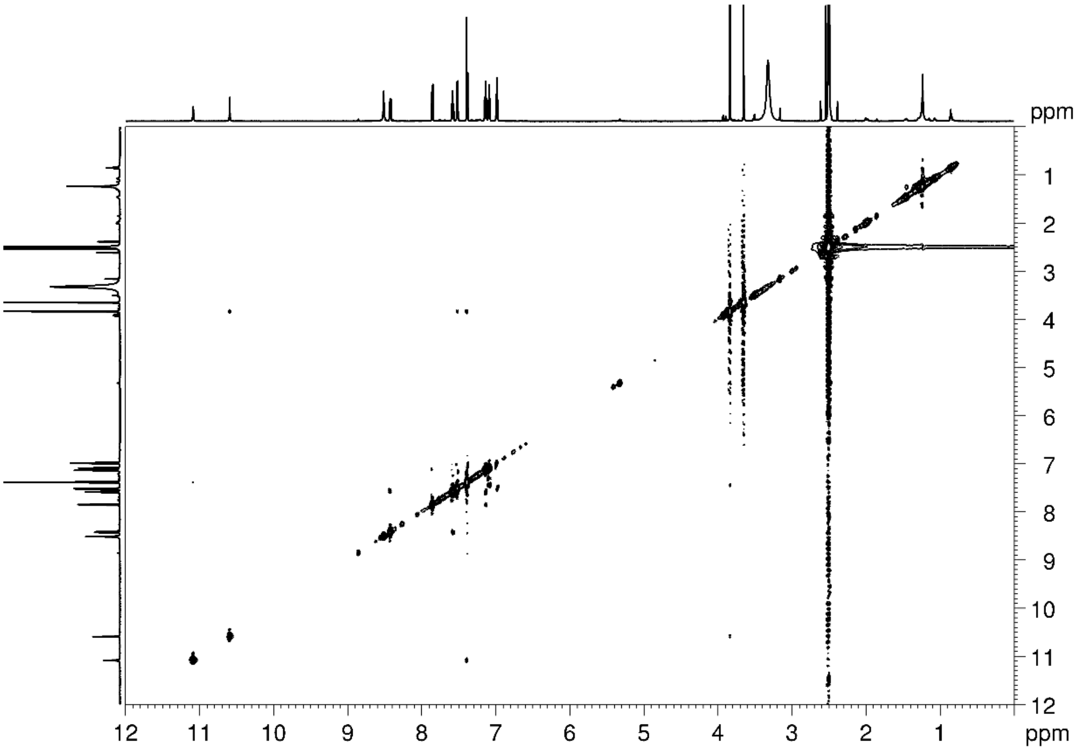

Figure S53. **^1H-^1H ROESY NMR spectrum of lindolin G (3g) in DMSO-*d*$_6$.**

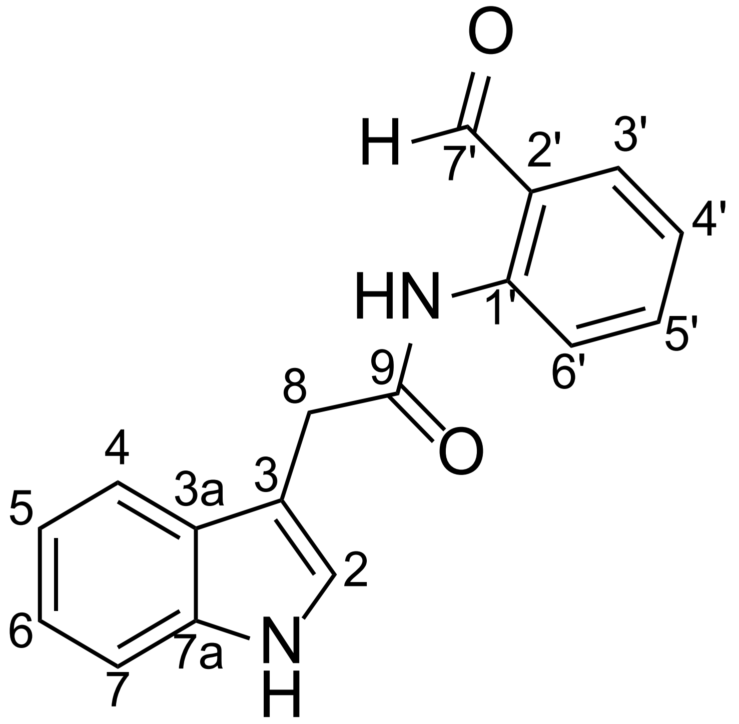

Figure S54. **Atom numbering of lindolin H (3h).**

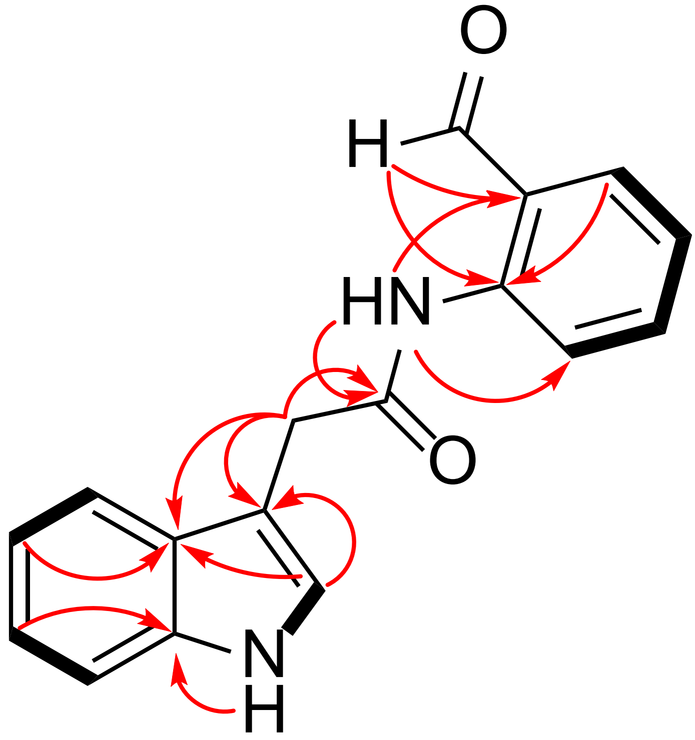

Figure S55. **^{1}H-^{1}H COSY (bold bonds) and ^{1}H-^{13}C HMBC (arrows) key correlations of lindolin H (3h).**

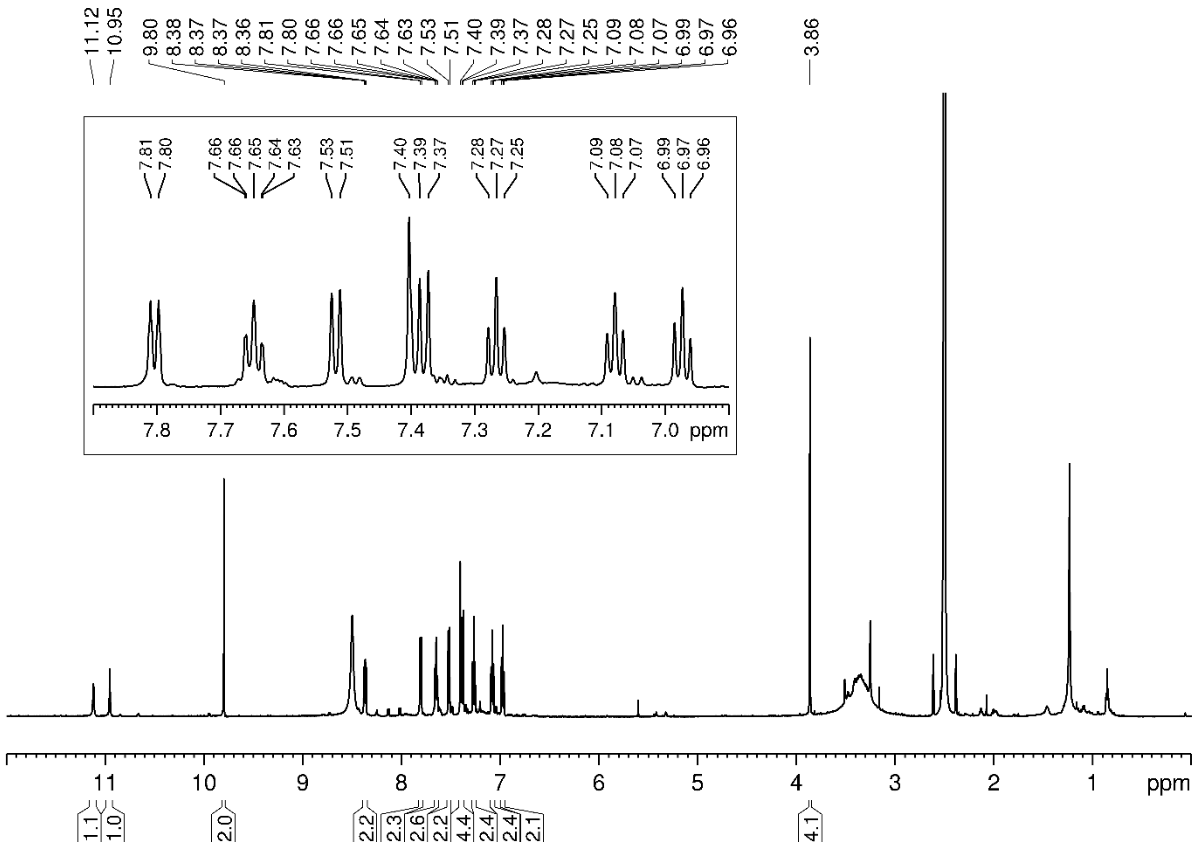

Figure S56. ^{1}H NMR spectrum of lindolin H (3h) in DMSO-d_6 recorded at 600 MHz.

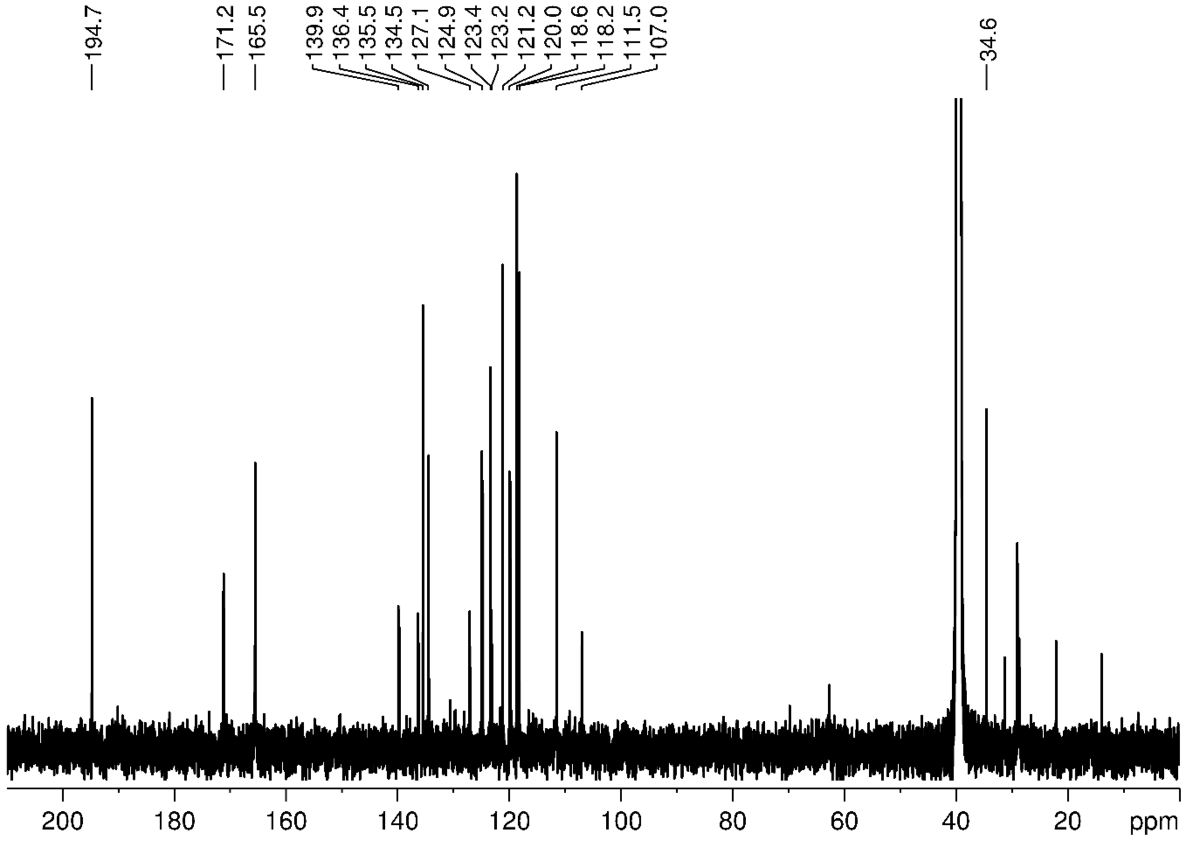

Figure S57. ^{13}C NMR spectrum of lindolin H (3h) in DMSO-d_6 recorded at 125 MHz.

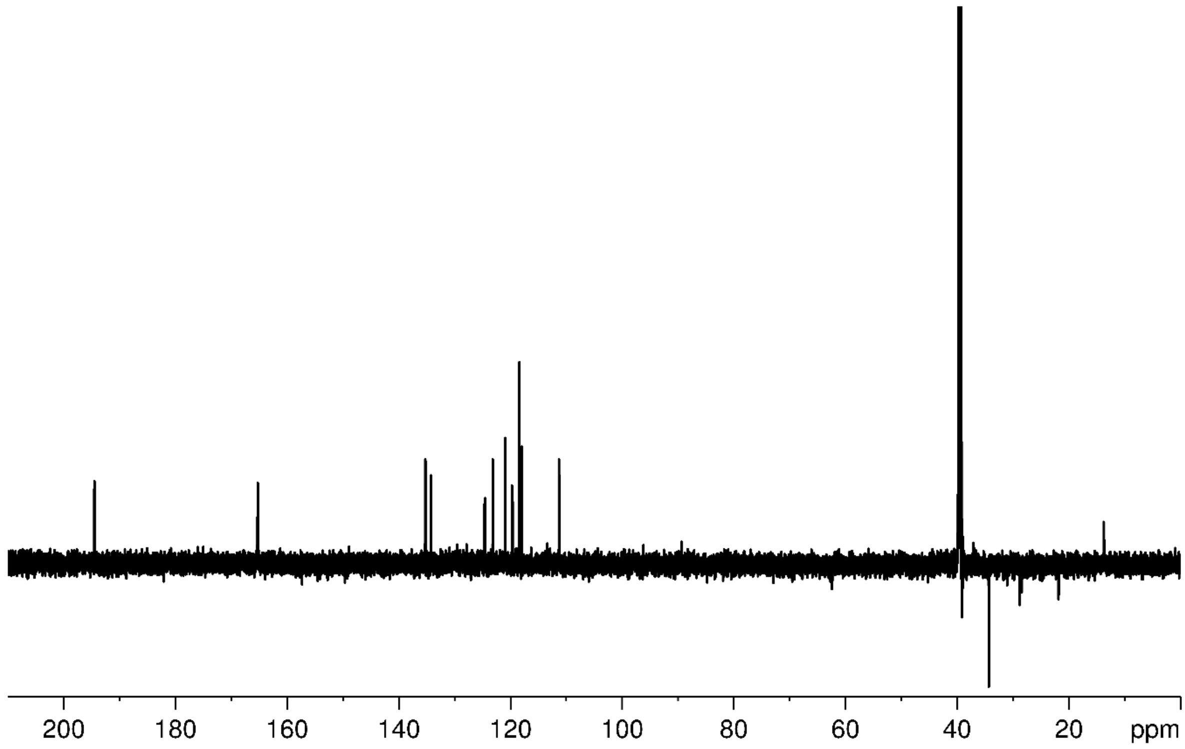

Figure S58. DEPT-135 NMR spectrum of lindolin H (3h) in DMSO-d_6.

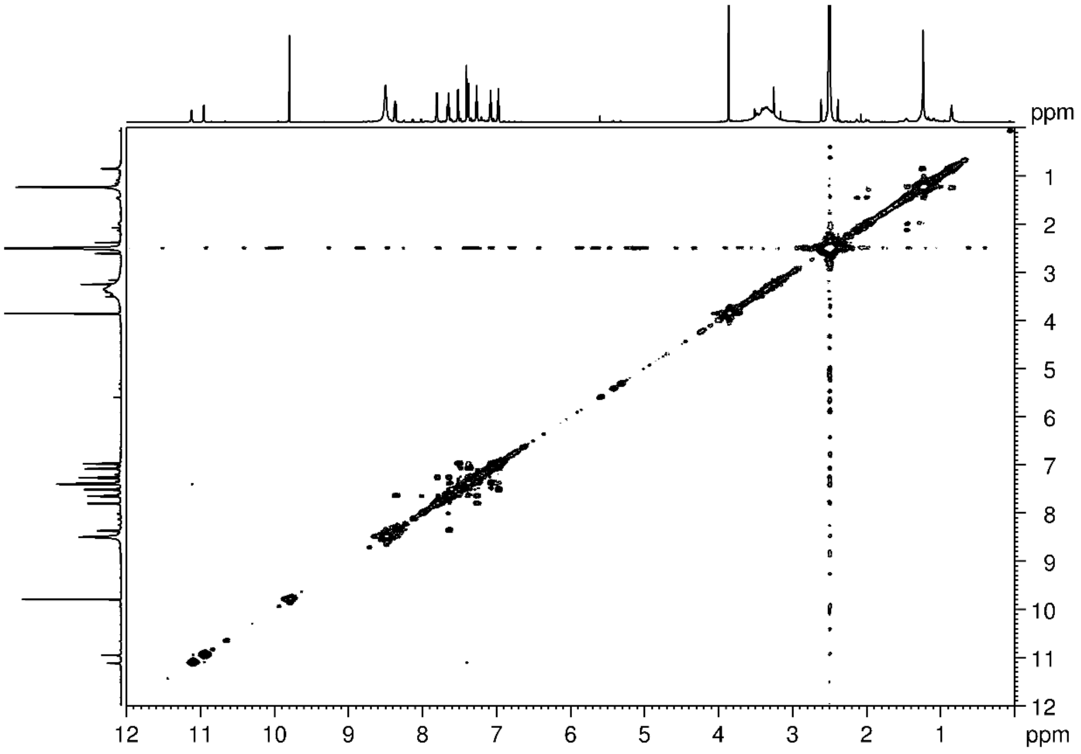

Figure S59. ^1H-^1H COSY NMR spectrum of lindolin H (3h) in DMSO-d_6.

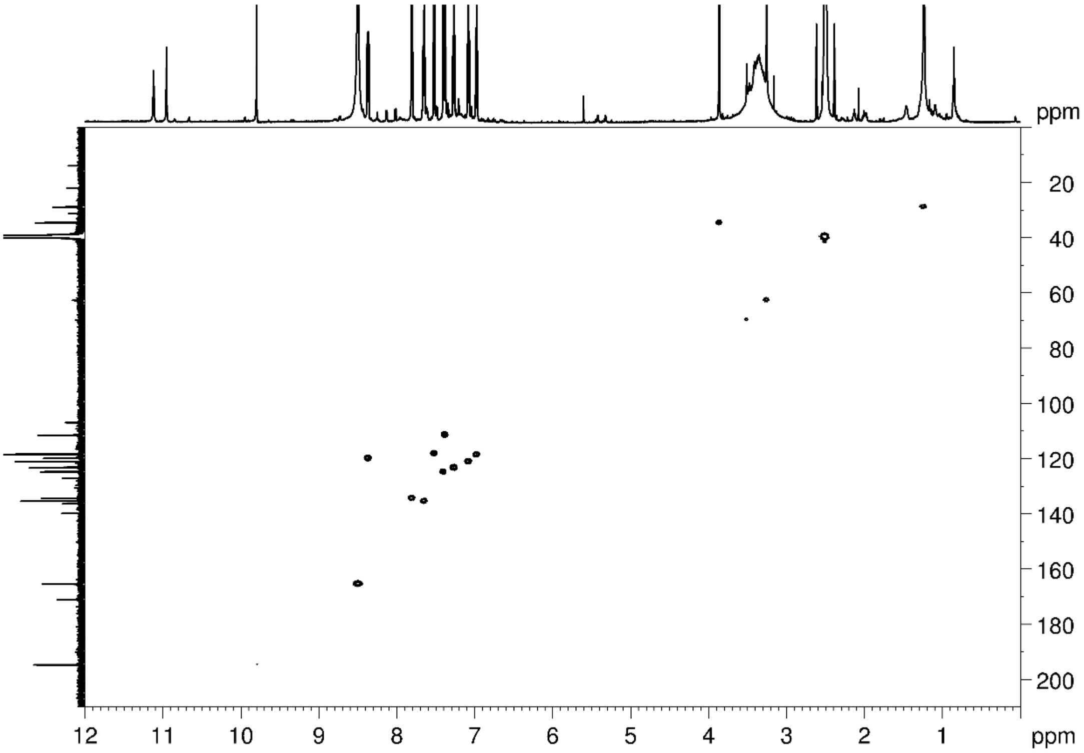

Figure S60. ^{1}H-^{13}C **HSQC NMR spectrum of lindolin H (3h) in DMSO-*d*$_6$.**

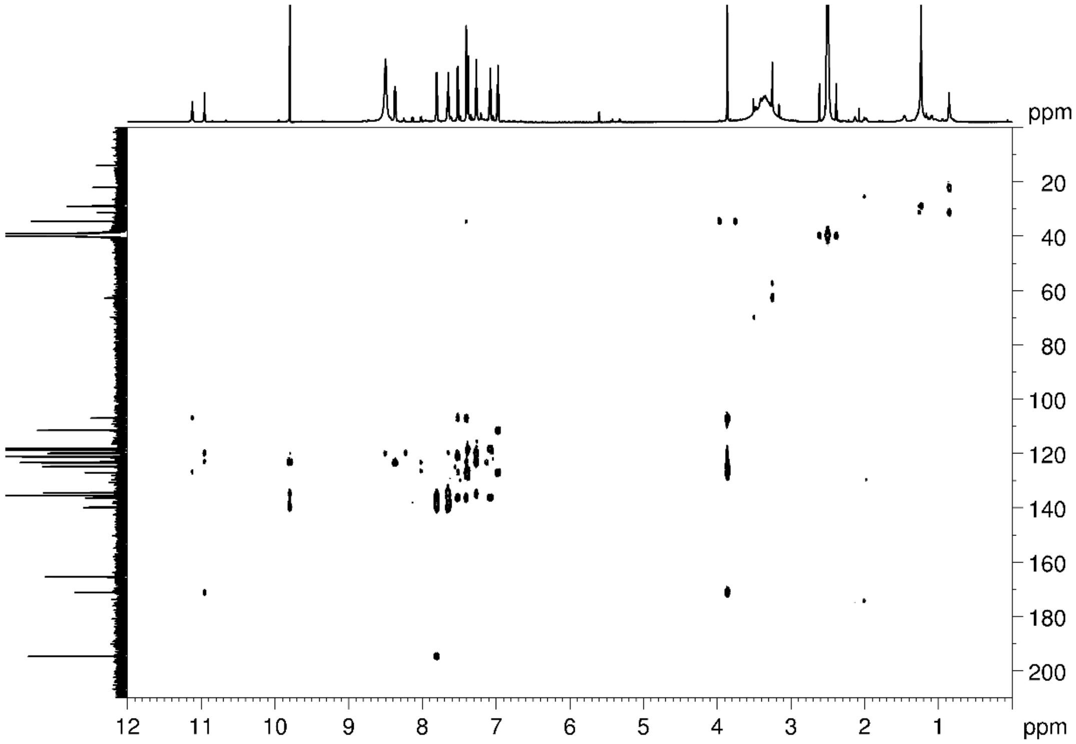

Figure S61. ^{1}H-^{13}C **HMBC NMR spectrum of lindolin H (3h) in DMSO-*d*$_6$.**

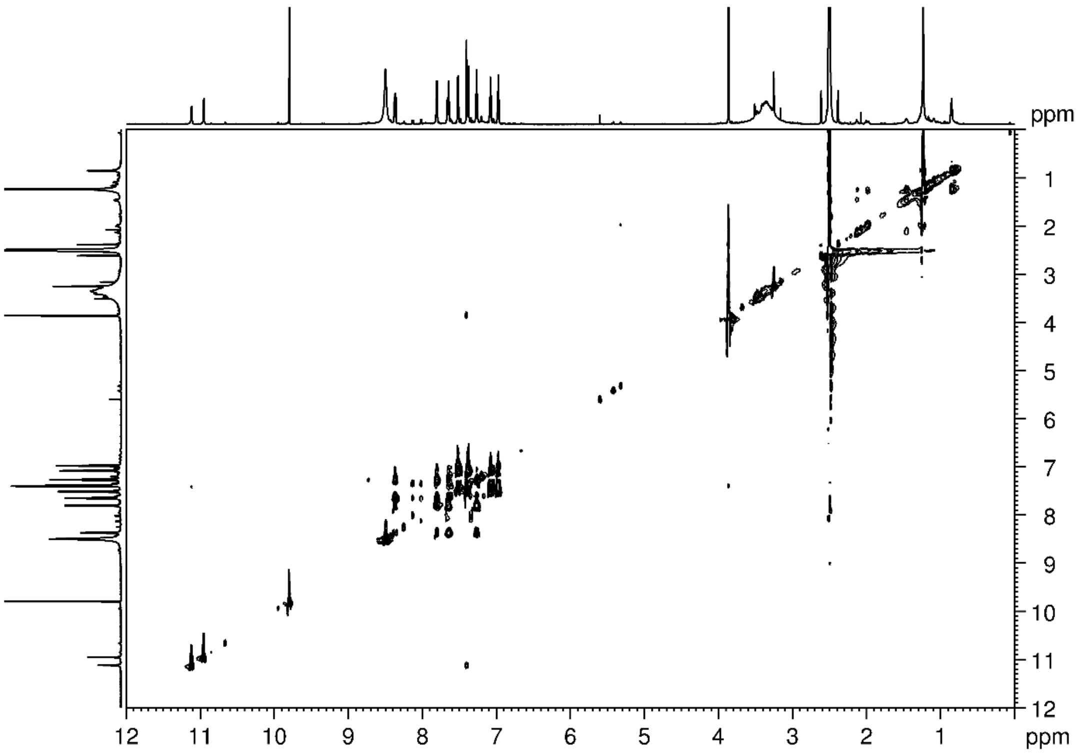

Figure S62. **^{1}H-^{1}H TOCSY NMR spectrum of lindolin H (3h) in DMSO-*d_6*.**

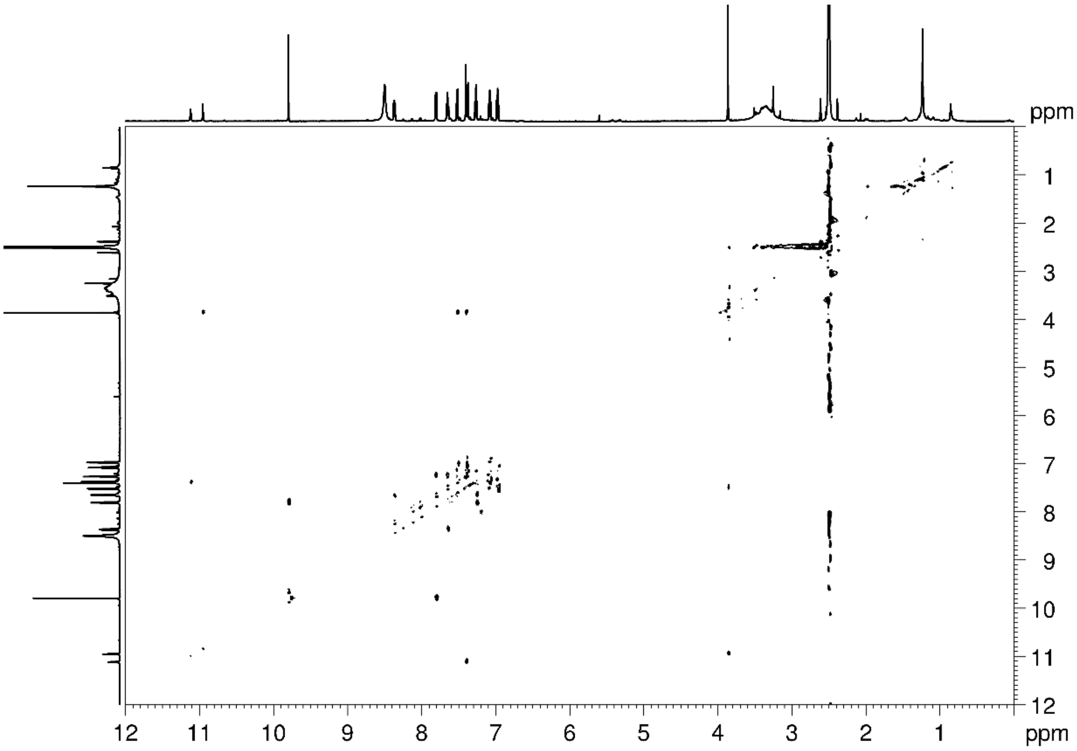

Figure S63. **^{1}H-^{1}H ROESY NMR spectrum of lindolin H (3h) in DMSO-*d_6*.**

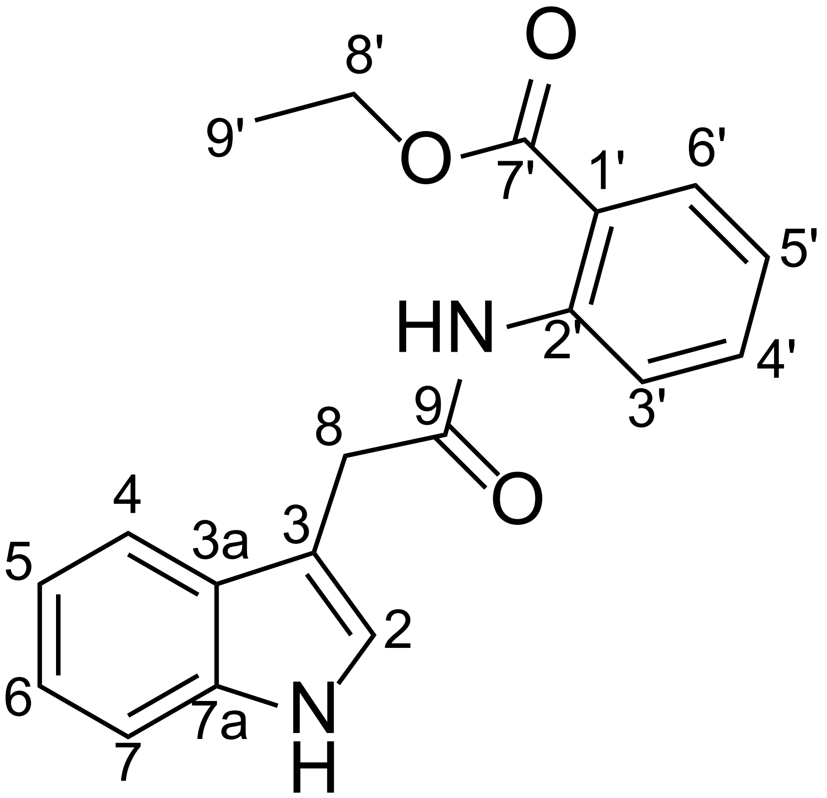

Figure S64. Atom numbering of lindolin I (3i).

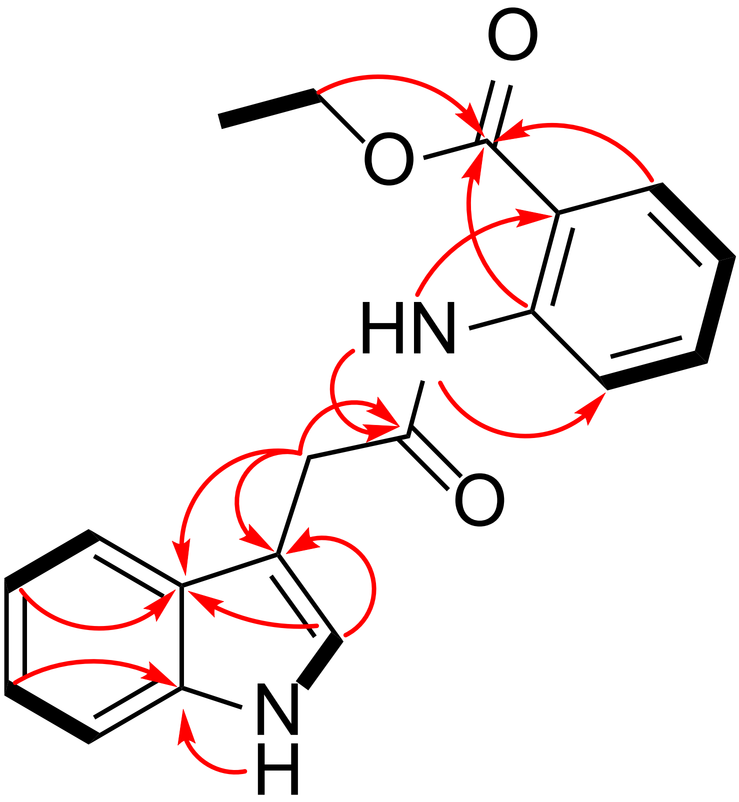

Figure S65. ^{1}H-^{1}H COSY (bold bonds) and ^{1}H-^{13}C HMBC (arrows) key correlations of lindolin I (3i).

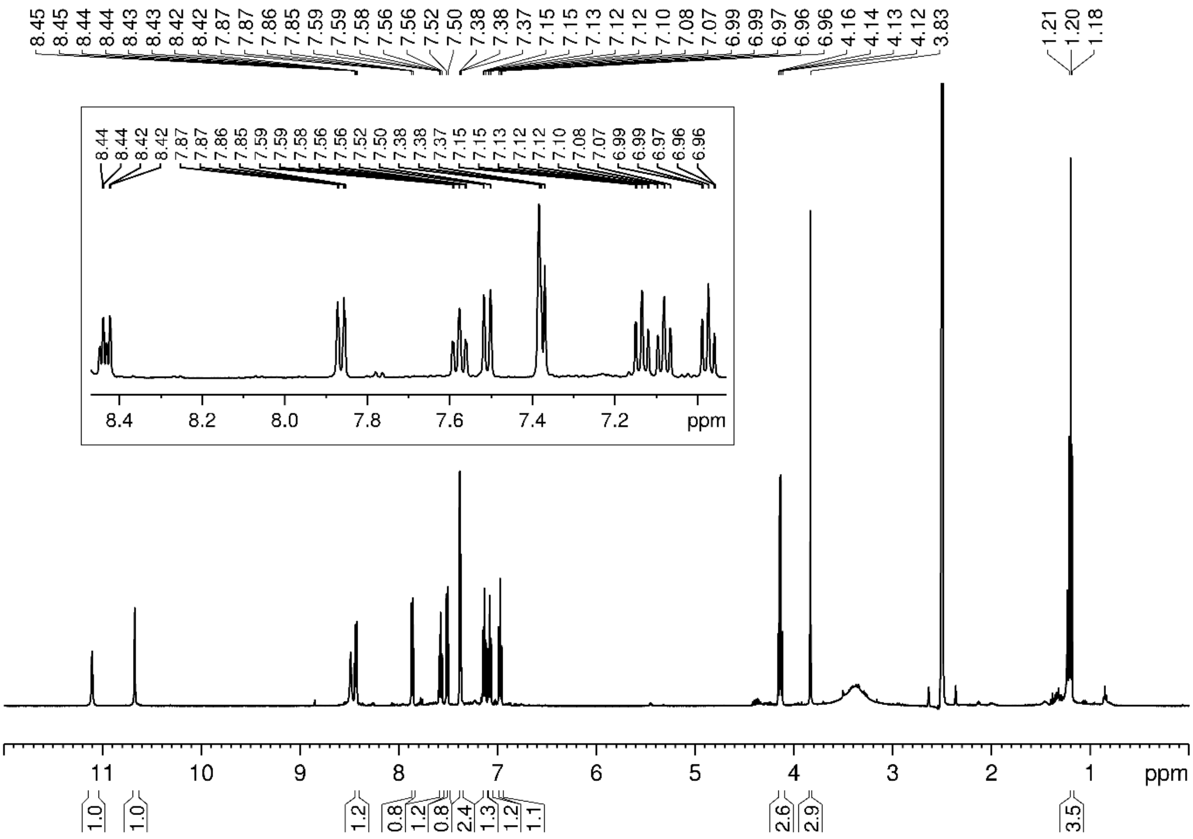

Figure S66. ^{1}H **NMR spectrum of lindolin I (3i) in DMSO-d_6 recorded at 500 MHz.**

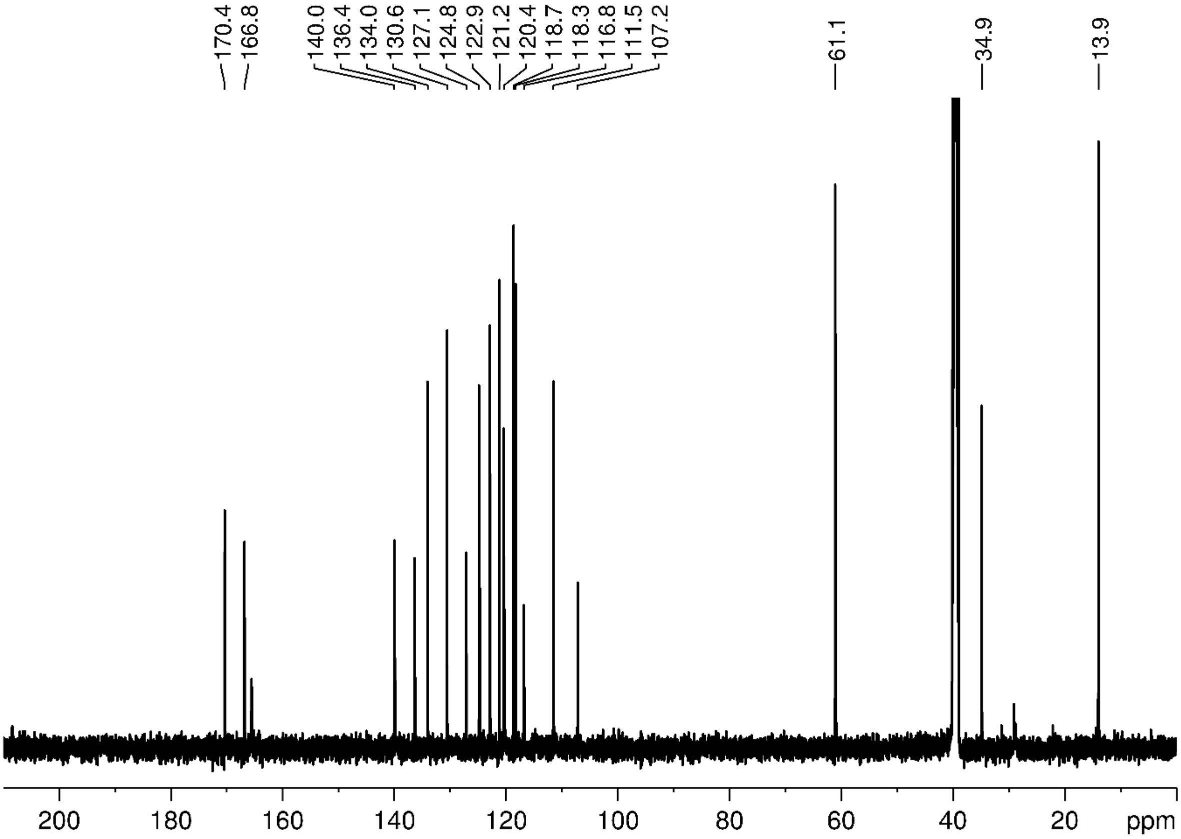

Figure S67. ^{13}C **NMR spectrum of lindolin I (3i) in DMSO-d_6 recorded at 125 MHz.**

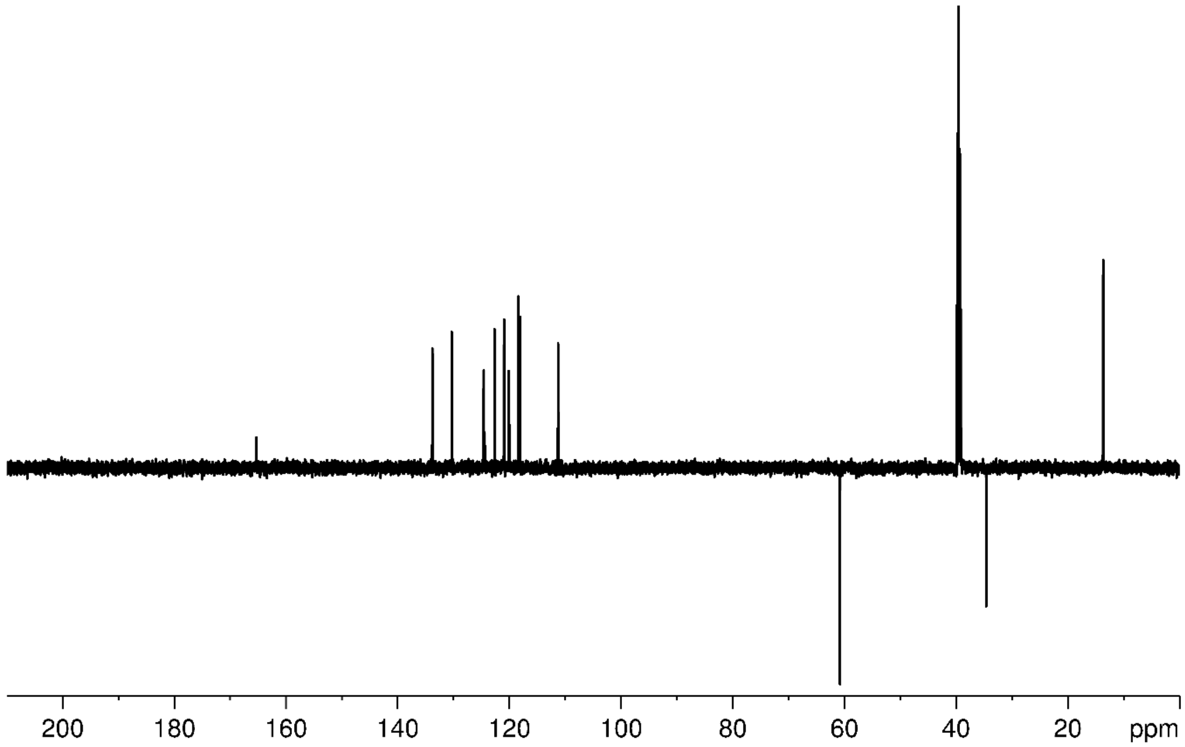

Figure S68. DEPT-135 NMR spectrum of lindolin I (3i) in DMSO-d_6.

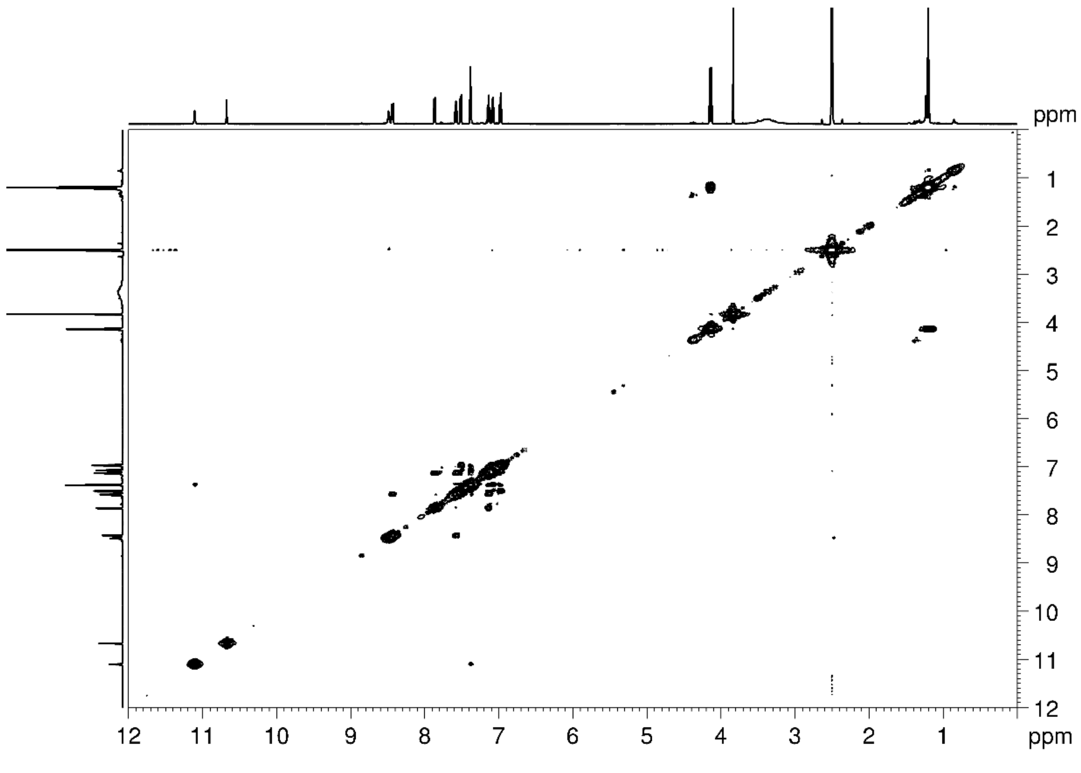

Figure S69. ^{1}H-^{1}H COSY NMR spectrum of lindolin I (3i) in DMSO-d_6.

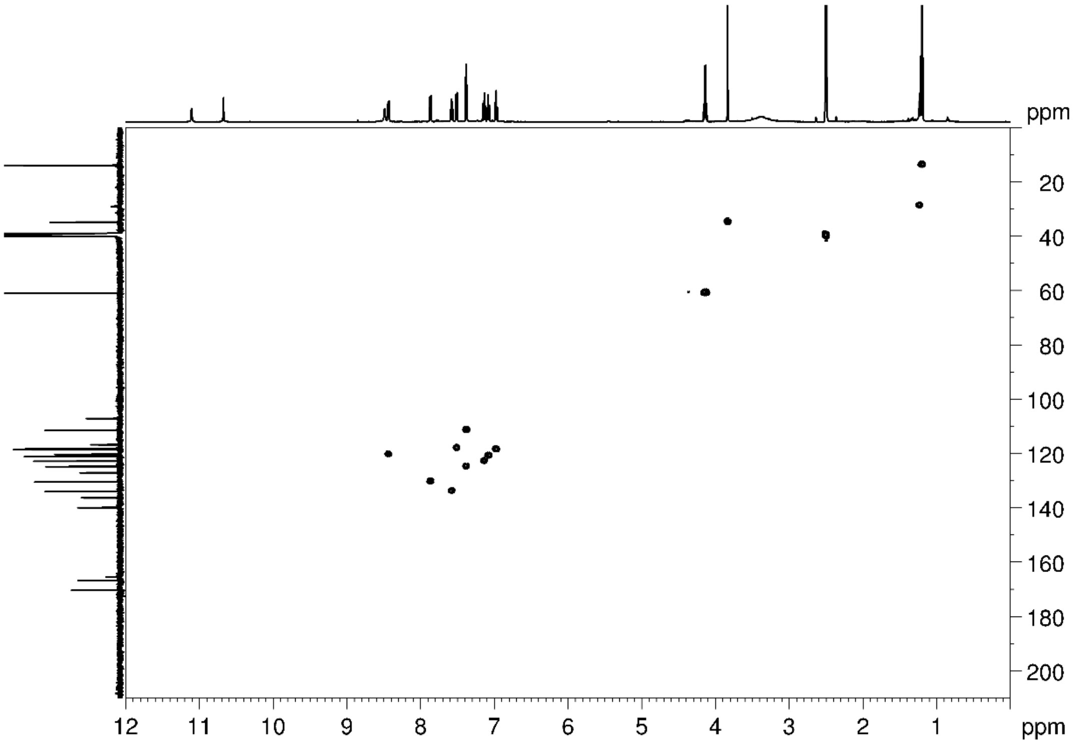

Figure S70. ^1H-^{13}C **HSQC NMR spectrum of lindolin I (3i) in DMSO-*d*$_6$.**

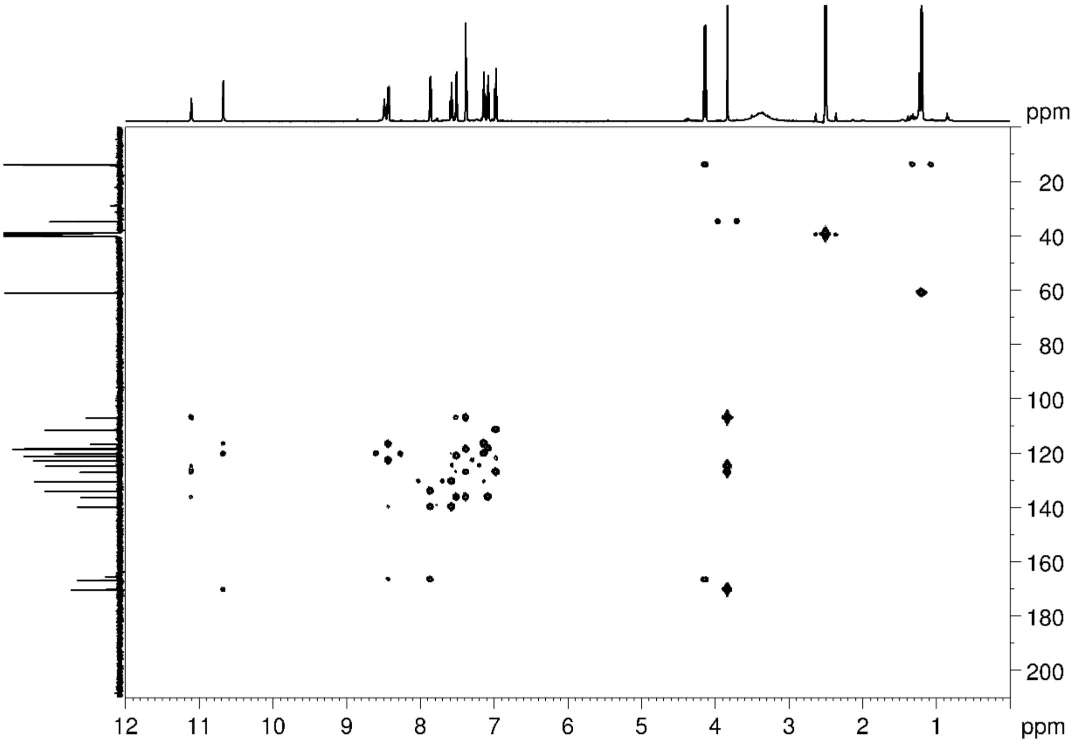

Figure S71. ^1H-^{13}C **HMBC NMR spectrum of lindolin I (3i) in DMSO-*d*$_6$.**

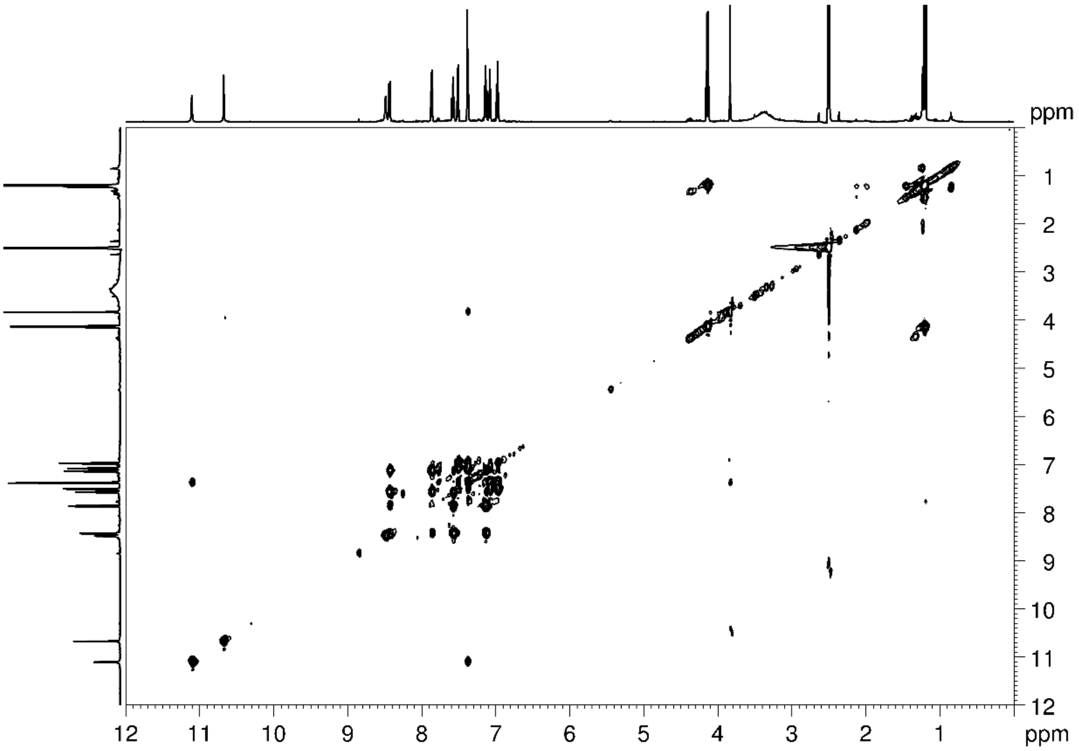

Figure S72. **^{1}H-^{1}H TOCSY NMR spectrum of lindolin I (3i) in DMSO-*d_6*.**

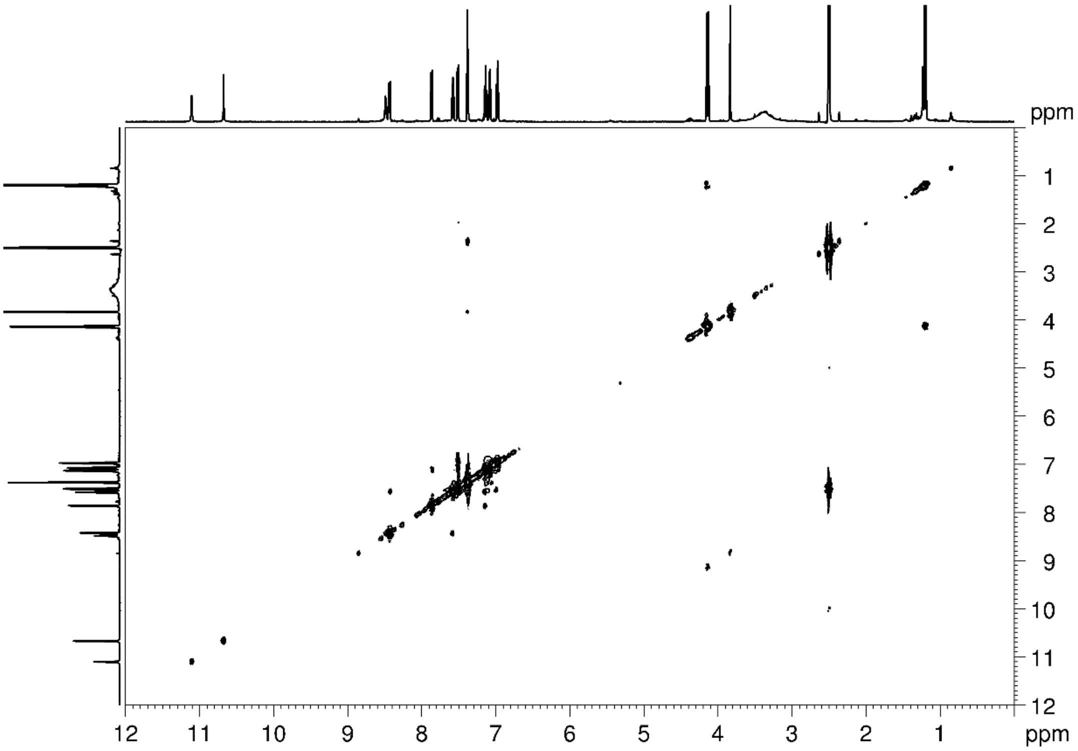

Figure S73. **^{1}H-^{1}H ROESY NMR spectrum of lindolin I (3i) in DMSO-*d_6*.**

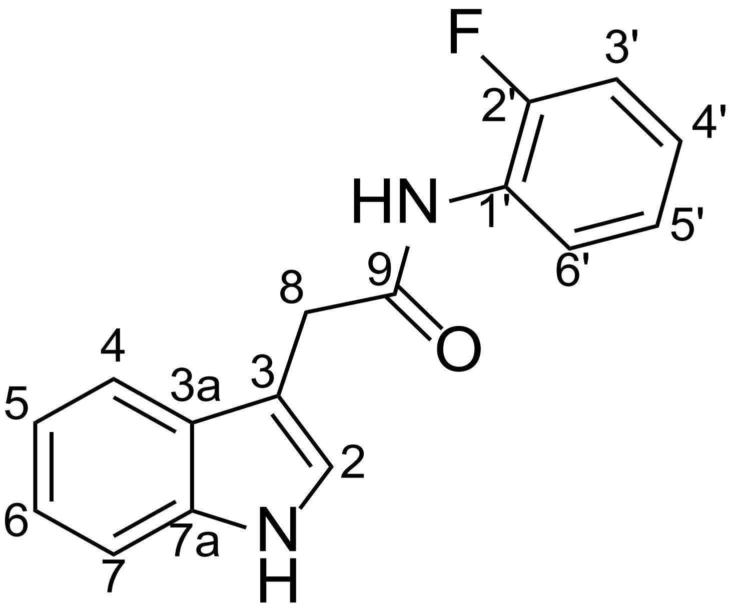

Figure S74. **Atom numbering of lindolin J (3j).**

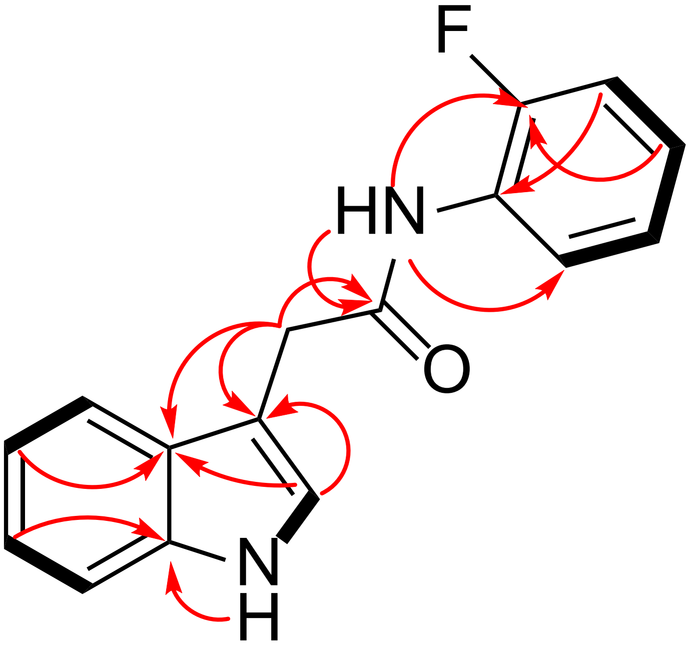

Figure S75. **^{1}H-^{1}H COSY (bold bonds) and ^{1}H-^{13}C HMBC (arrows) key correlations of lindolin J (3j).**

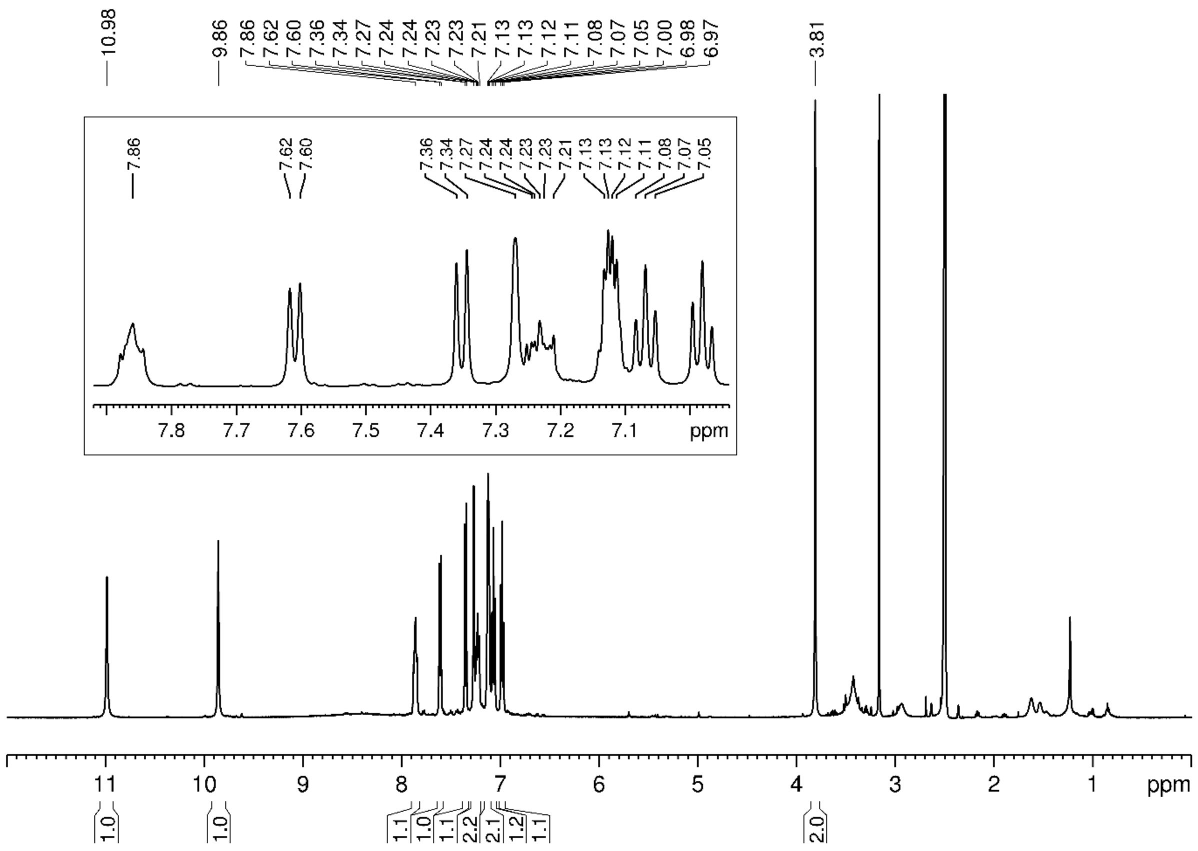

Figure S76. ^{1}**H NMR spectrum of lindolin J (3j) in DMSO-d_6 recorded at 500 MHz.**

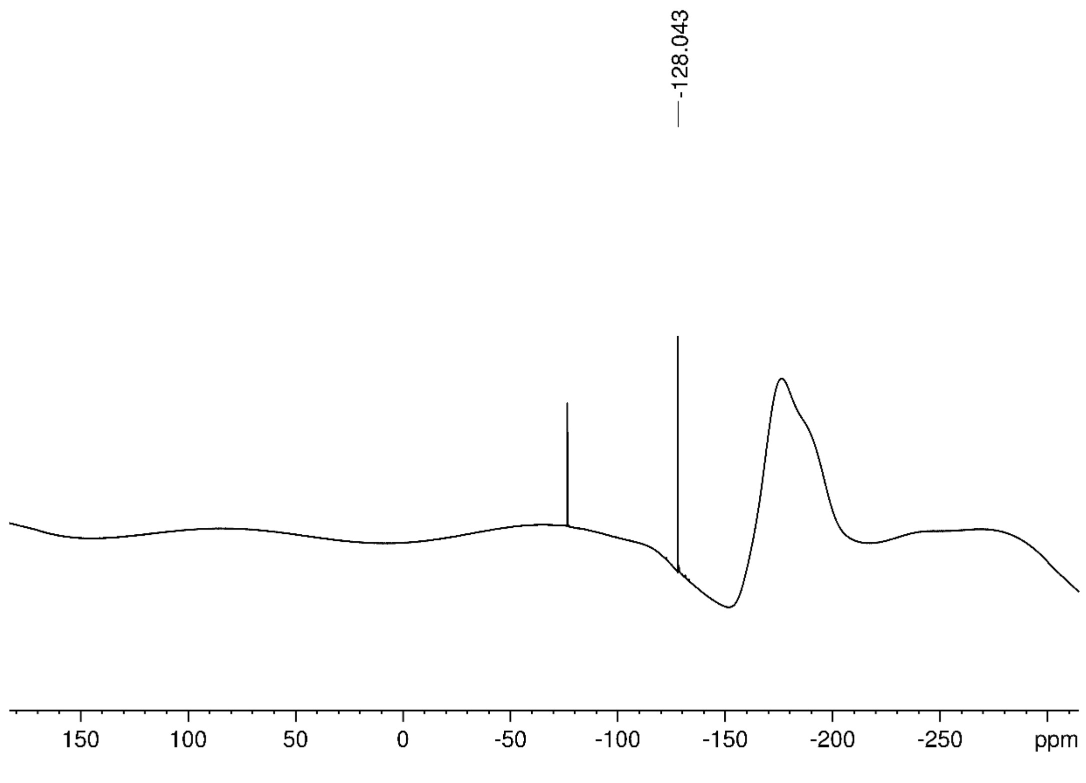

Figure S77. ^{19}**F NMR spectrum of lindolin J (3j) in DMSO-d_6 recorded at 470 MHz.**

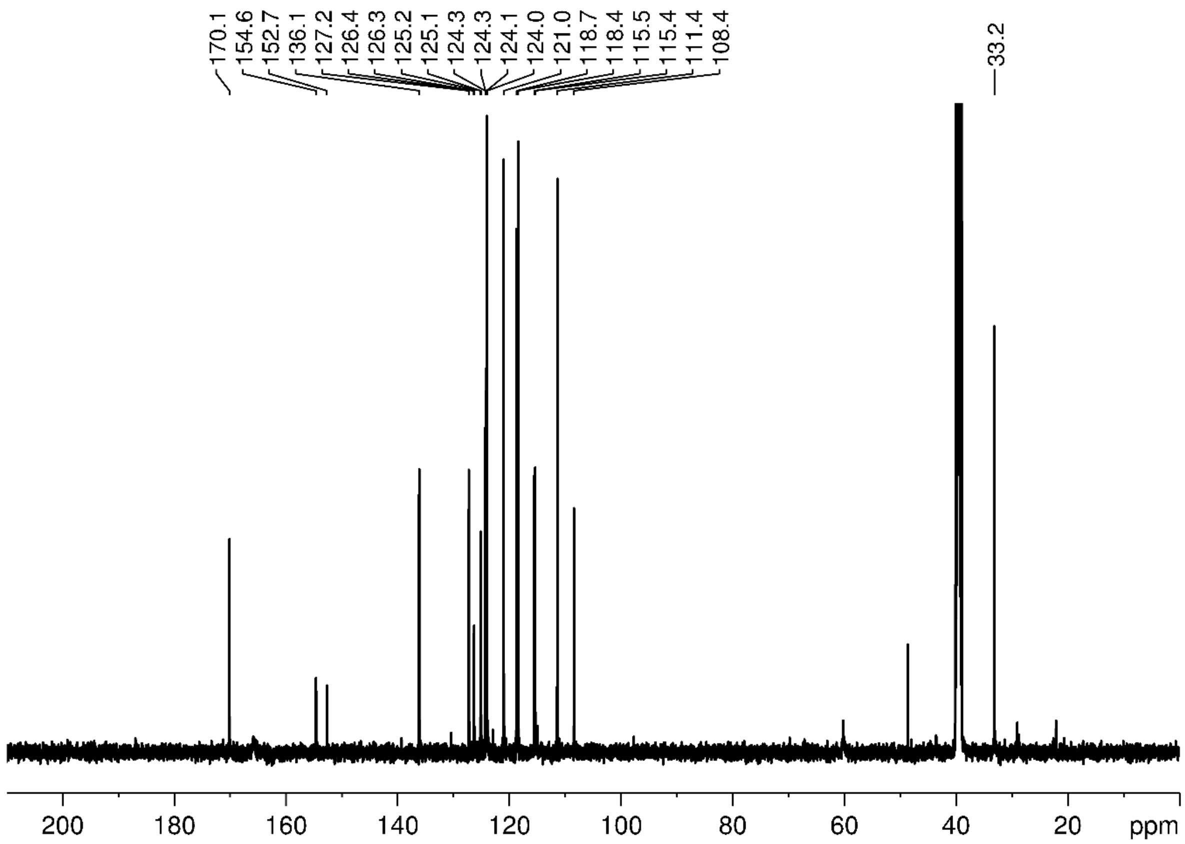

Figure S78. ^{13}C NMR spectrum of lindolin J (3j) in DMSO-d_6 recorded at 125 MHz.

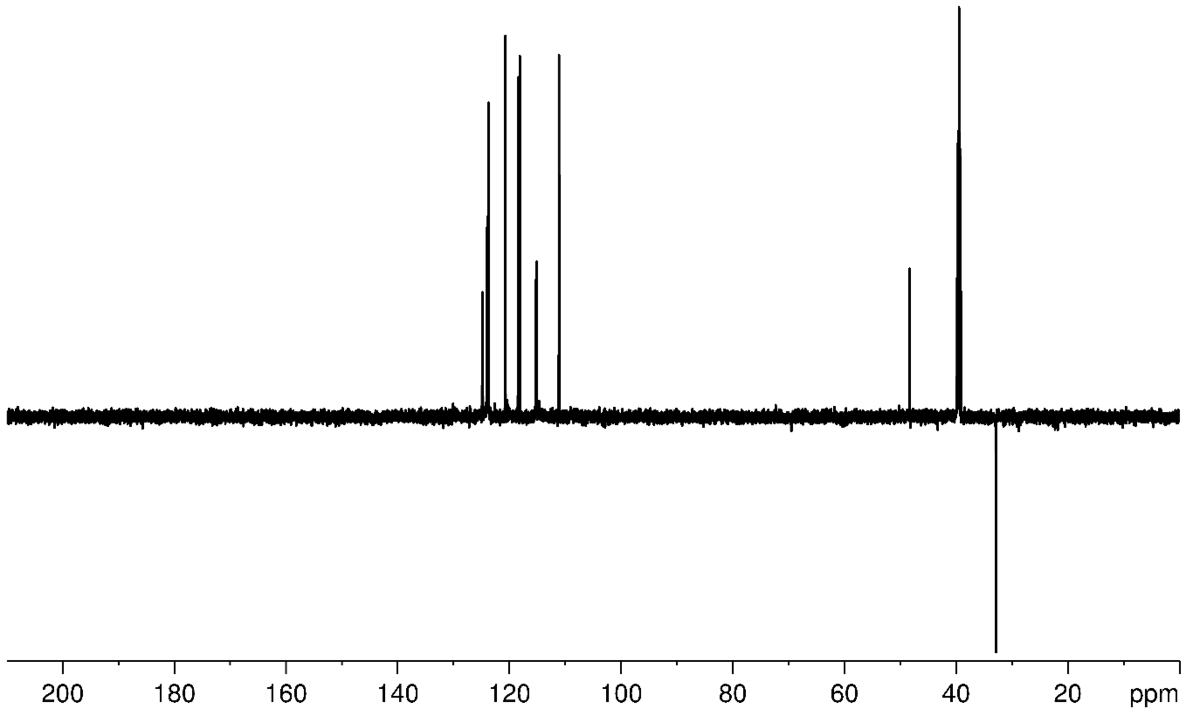

Figure S79. DEPT-135 NMR spectrum of lindolin J (3j) in DMSO-d_6.

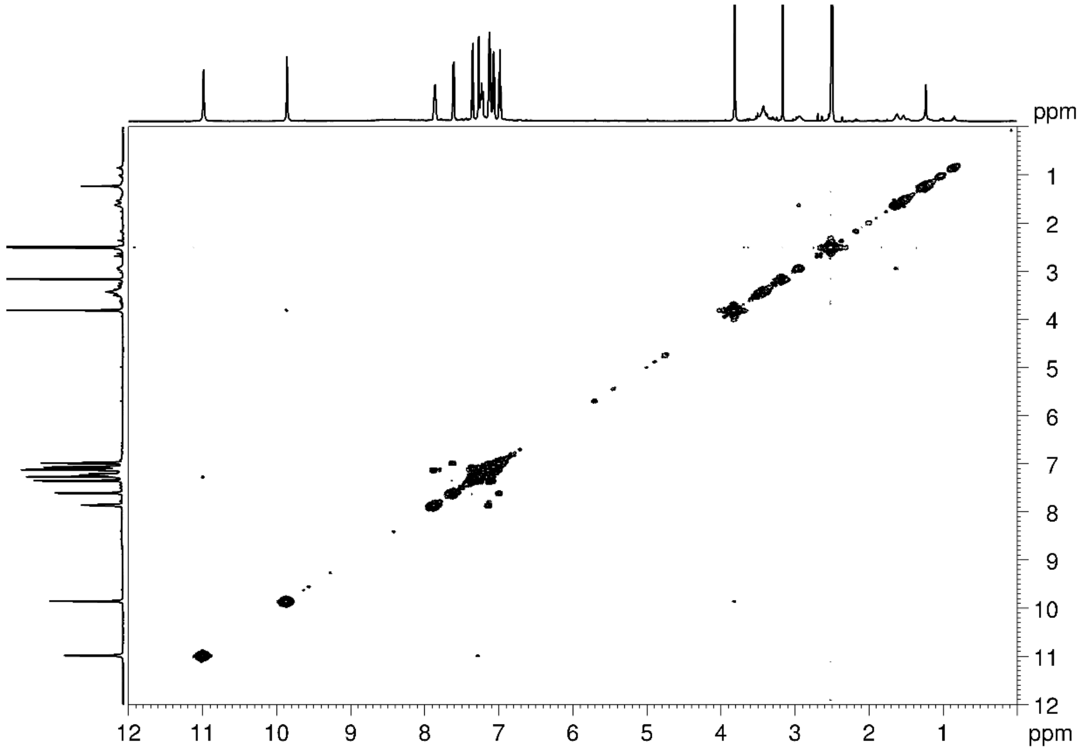

Figure S80. ^1H-^1H **COSY NMR spectrum of lindolin J (3j) in DMSO-*d*$_6$.**

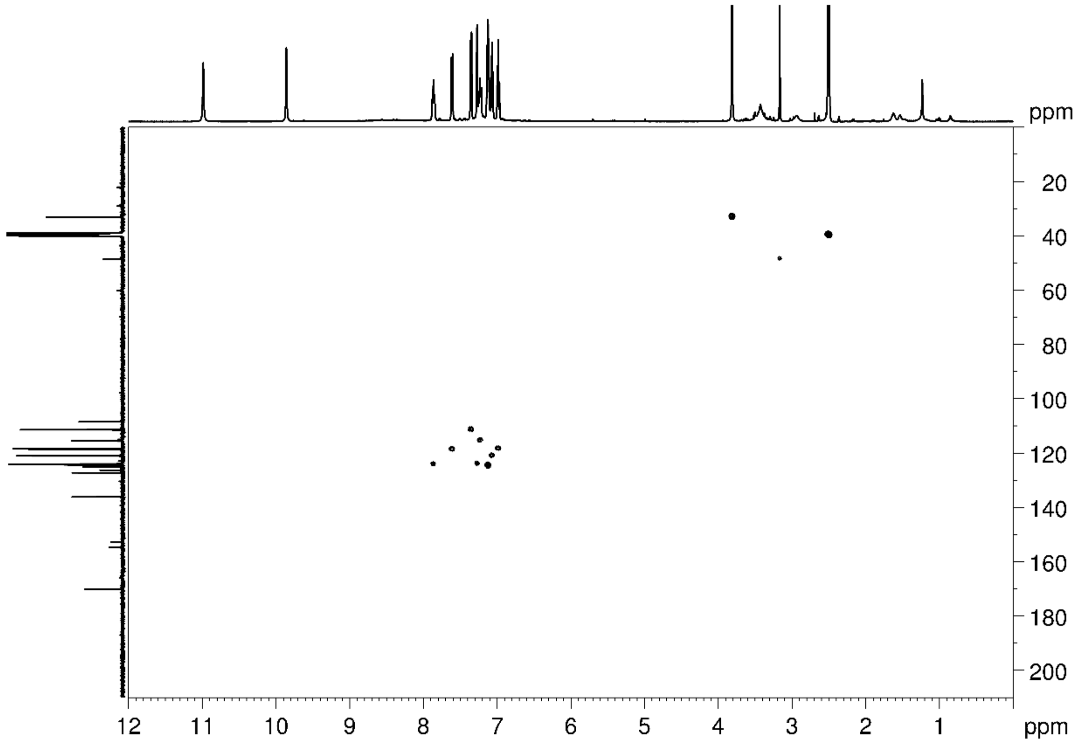

Figure S81. ^1H-^{13}C **HSQC NMR spectrum of lindolin J (3j) in DMSO-*d*$_6$.**

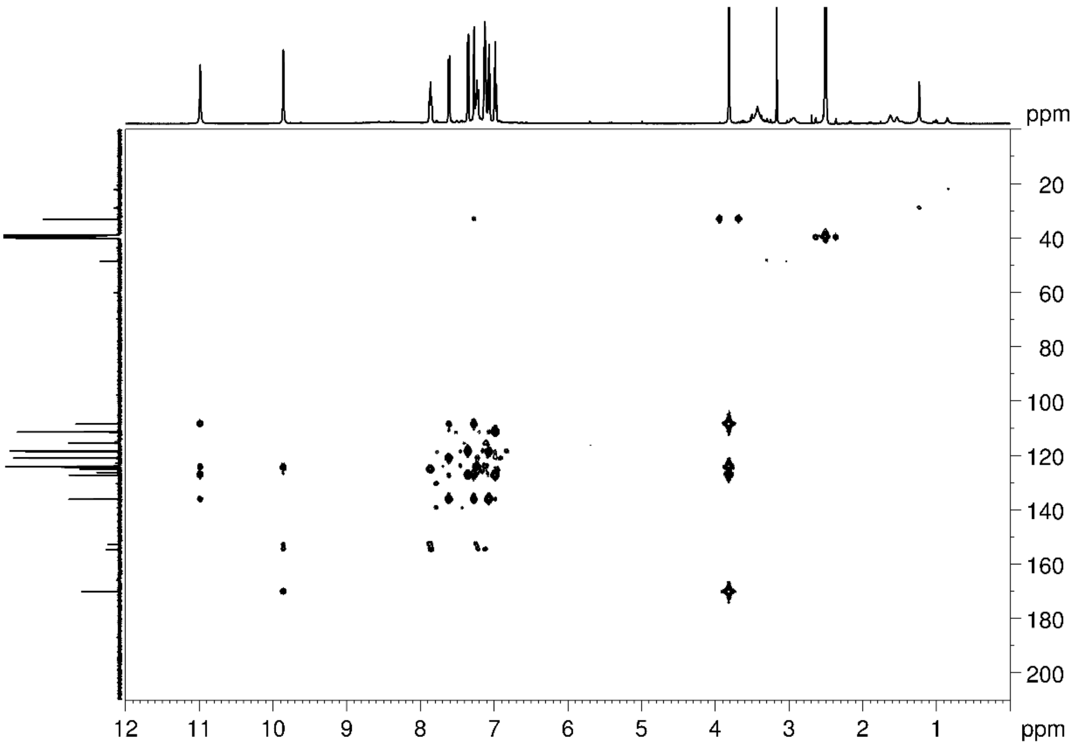

Figure S82. ^{1}H-^{13}C **HMBC NMR spectrum of lindolin J (3j) in DMSO-*d*$_6$.**

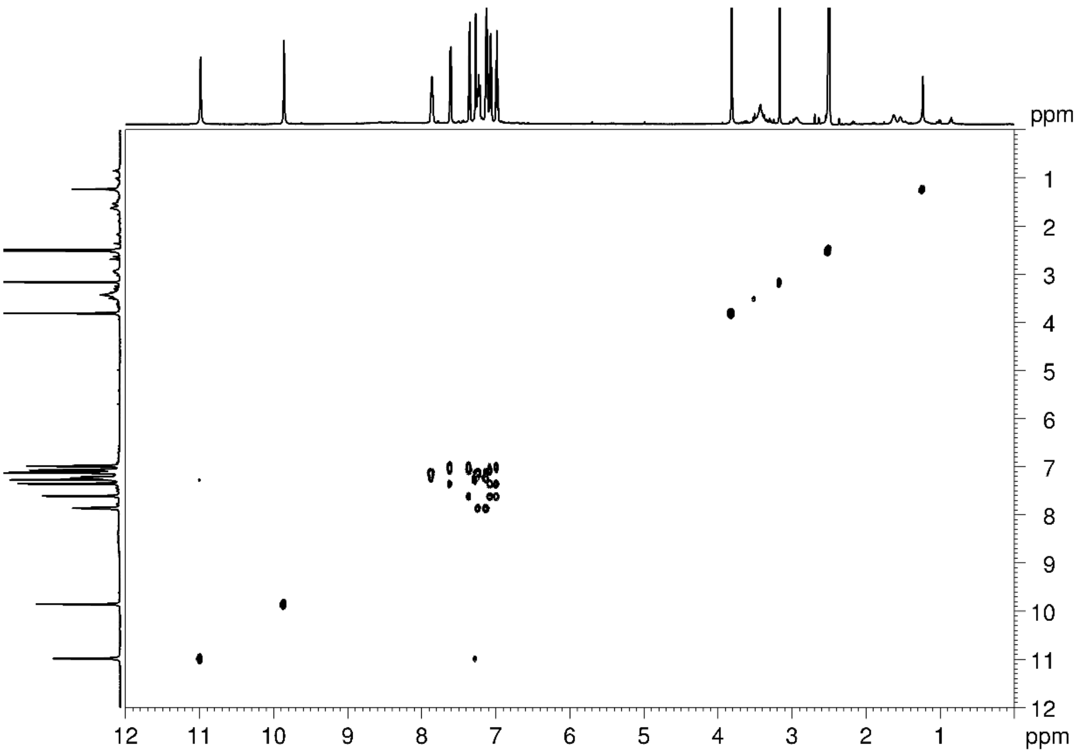

Figure S83. ^{1}H-^{1}H **TOCSY NMR spectrum of lindolin J (3j) in DMSO-*d*$_6$.**

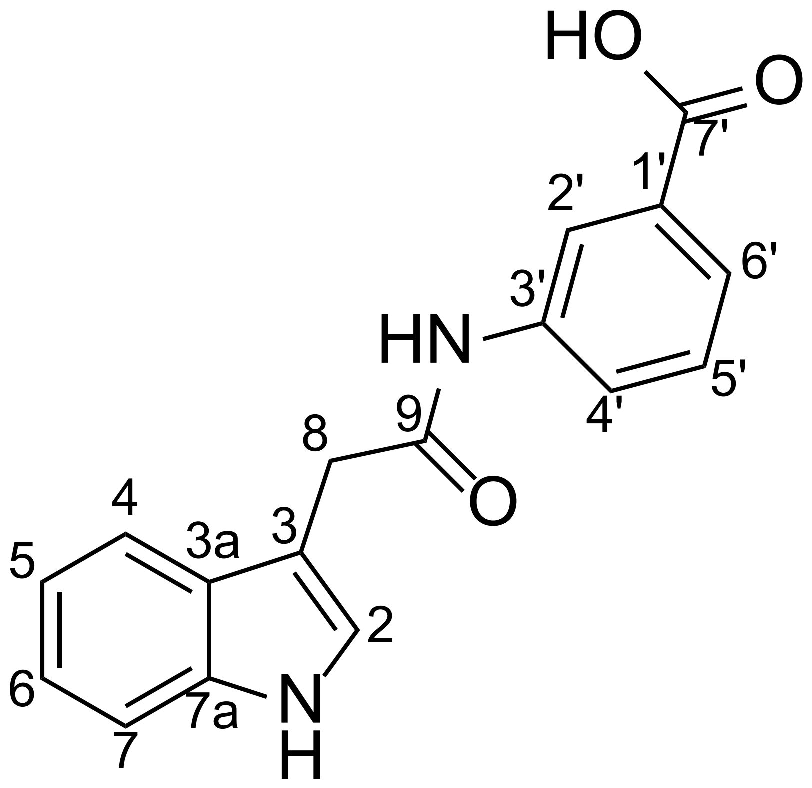

Figure S84. **Atom numbering of lindolin K (3k).**

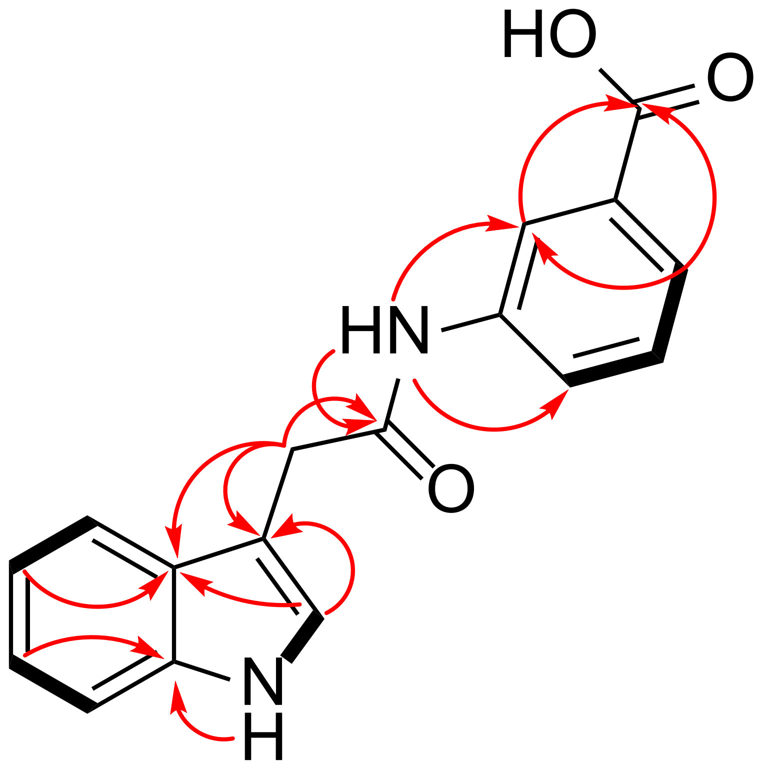

Figure S85. **^{1}H-^{1}H COSY (bold bonds) and ^{1}H-^{13}C HMBC (arrows) key correlations of lindolin K (3k).**

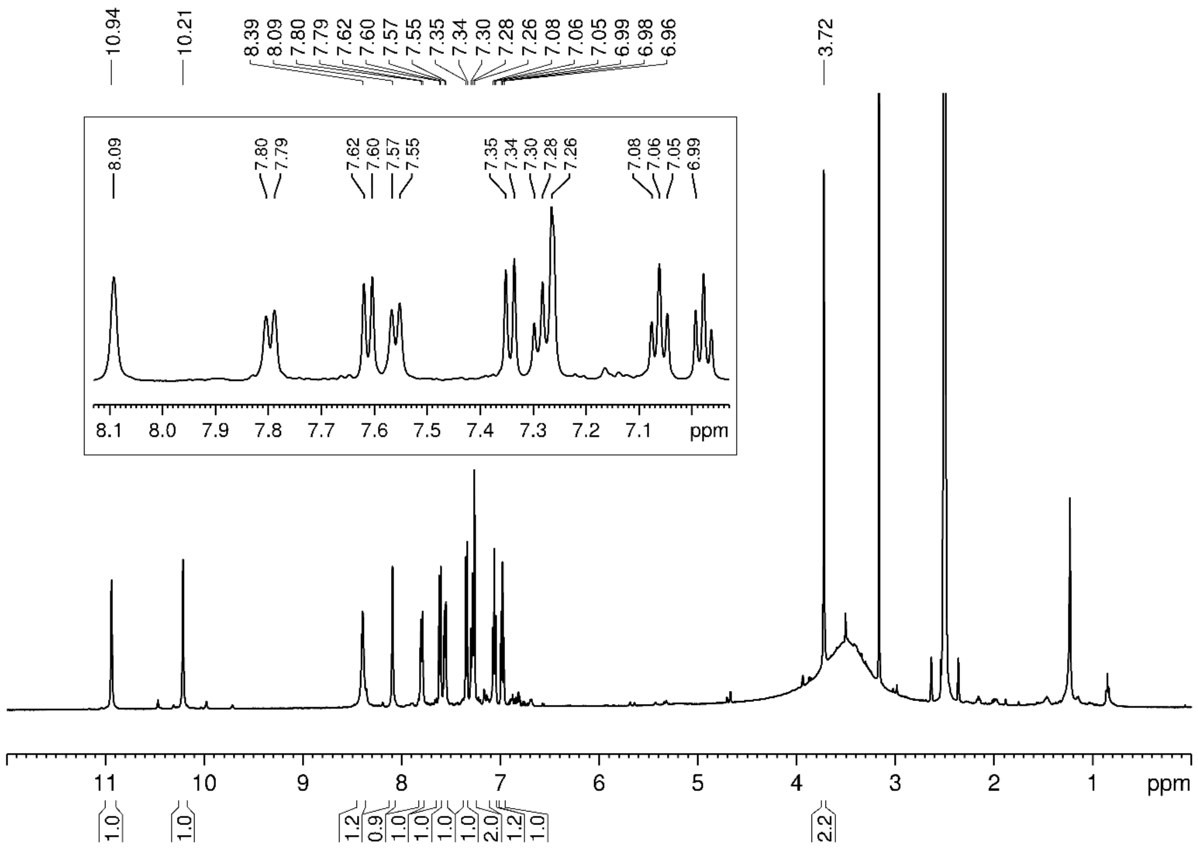

Figure S86. ^{1}H NMR spectrum of lindolin K (3k) in DMSO-d_6 recorded at 500 MHz.

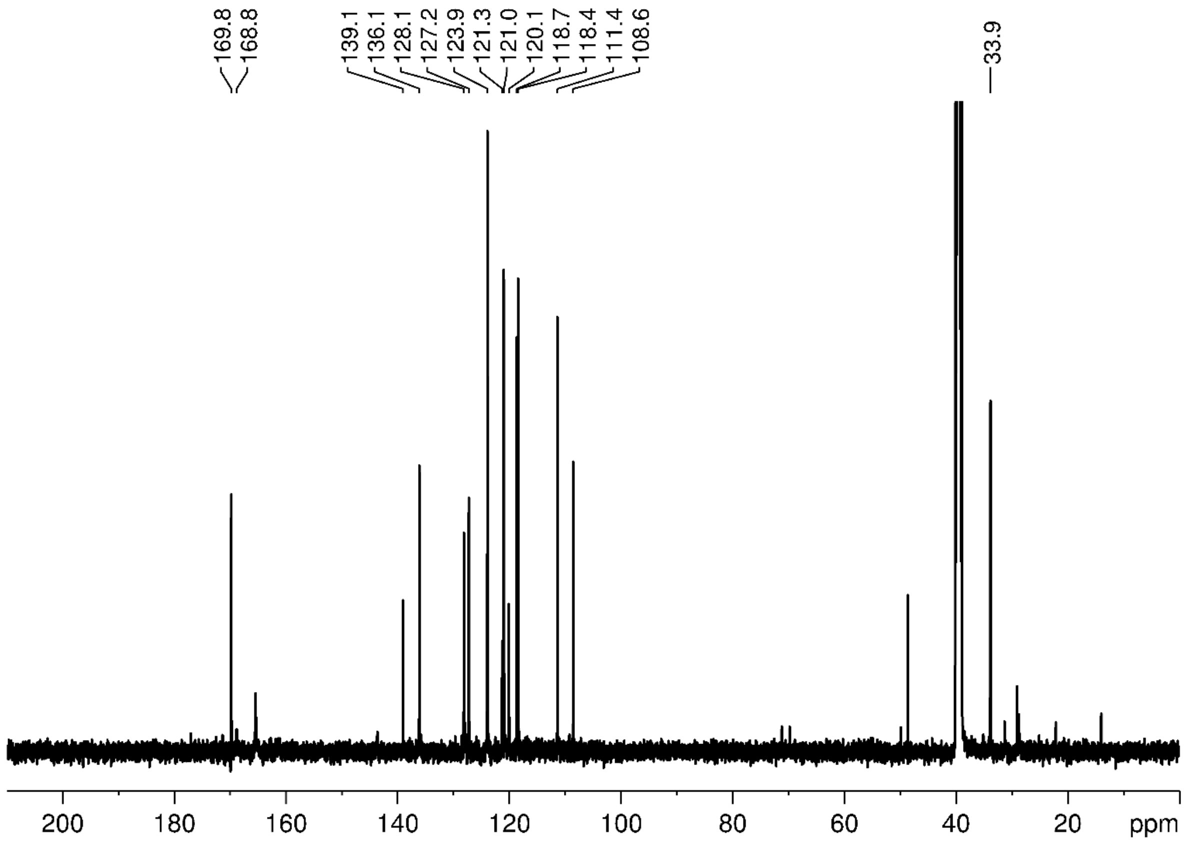

Figure S87. ^{13}C NMR spectrum of lindolin K (3k) in DMSO-d_6 recorded at 125 MHz.

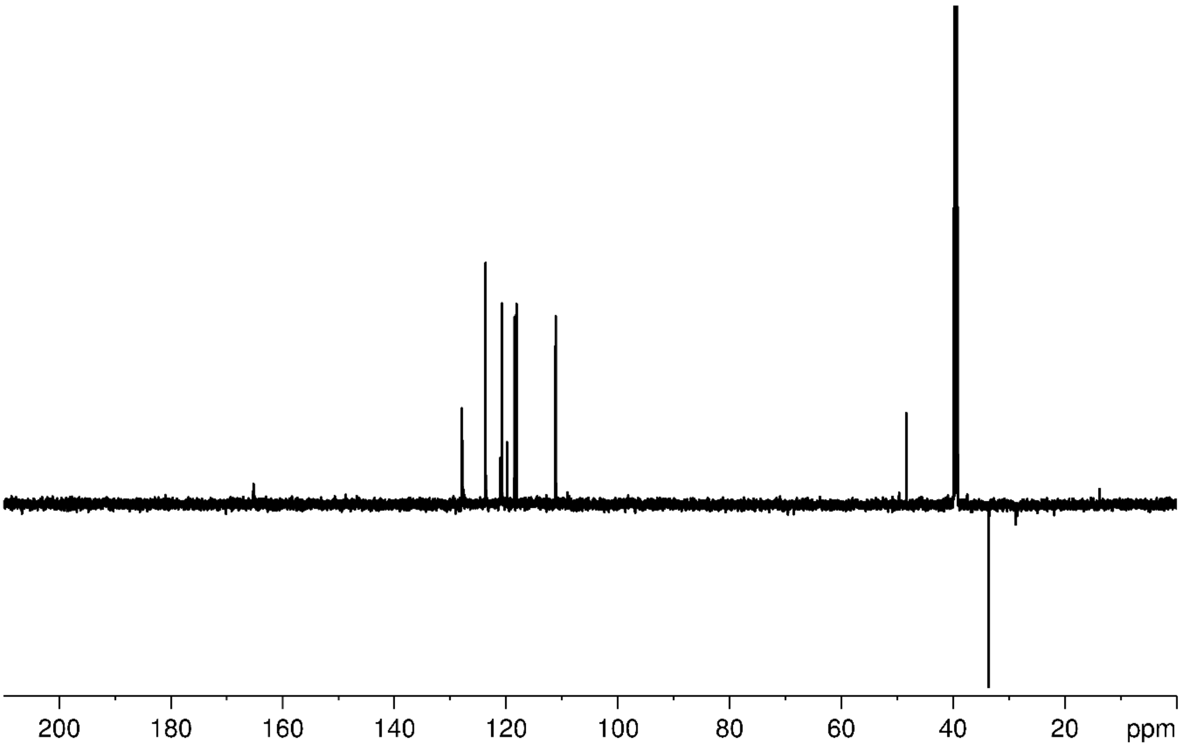

Figure S88. DEPT-135 NMR spectrum of lindolin K (3k) in DMSO-d_6.

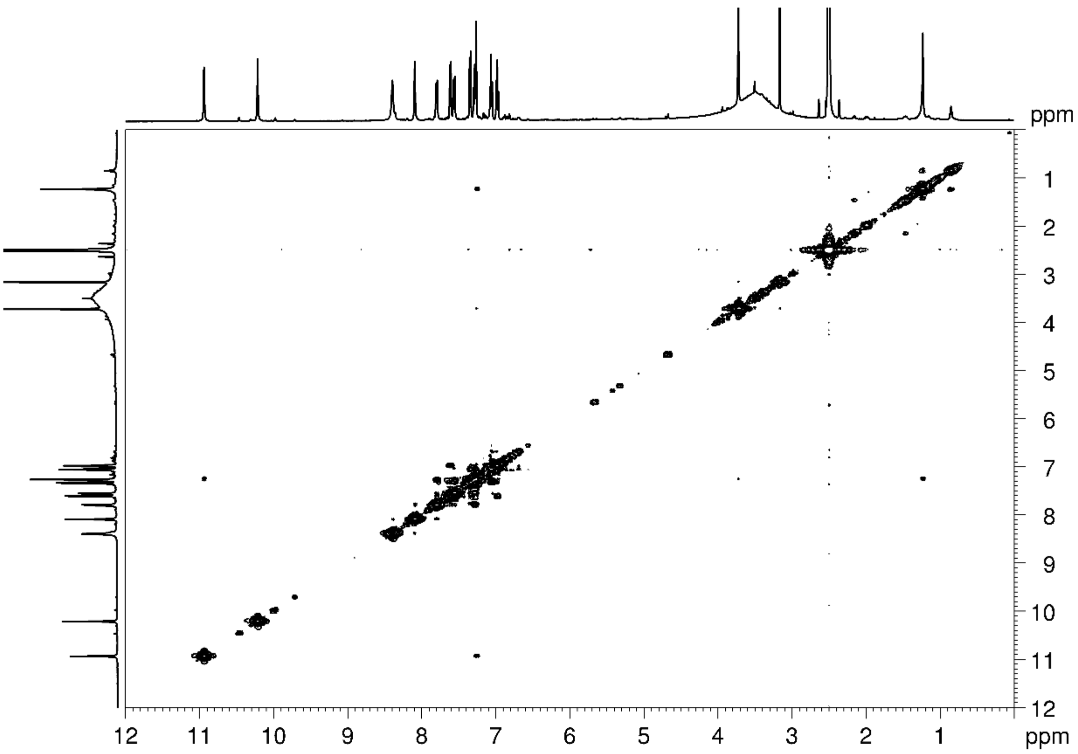

Figure S89. ^1H-^1H COSY NMR spectrum of lindolin K (3k) in DMSO-d_6.

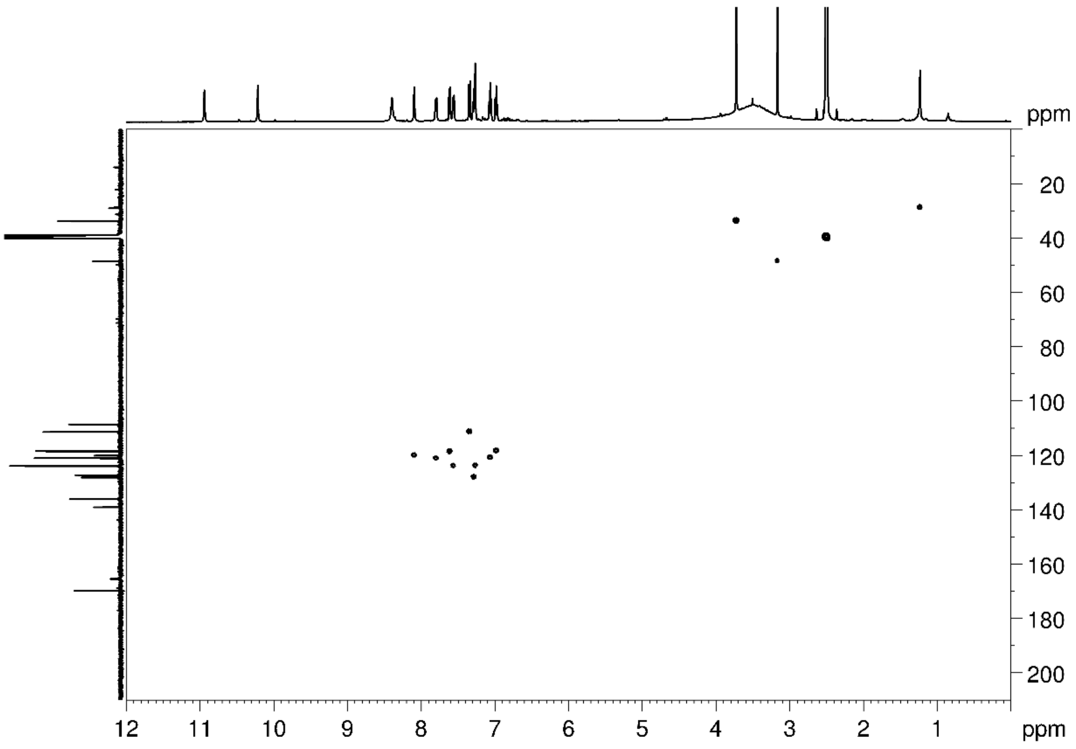

Figure S90. ^{1}H-^{13}C **HSQC NMR spectrum of lindolin K (3k) in DMSO-*d*$_6$.**

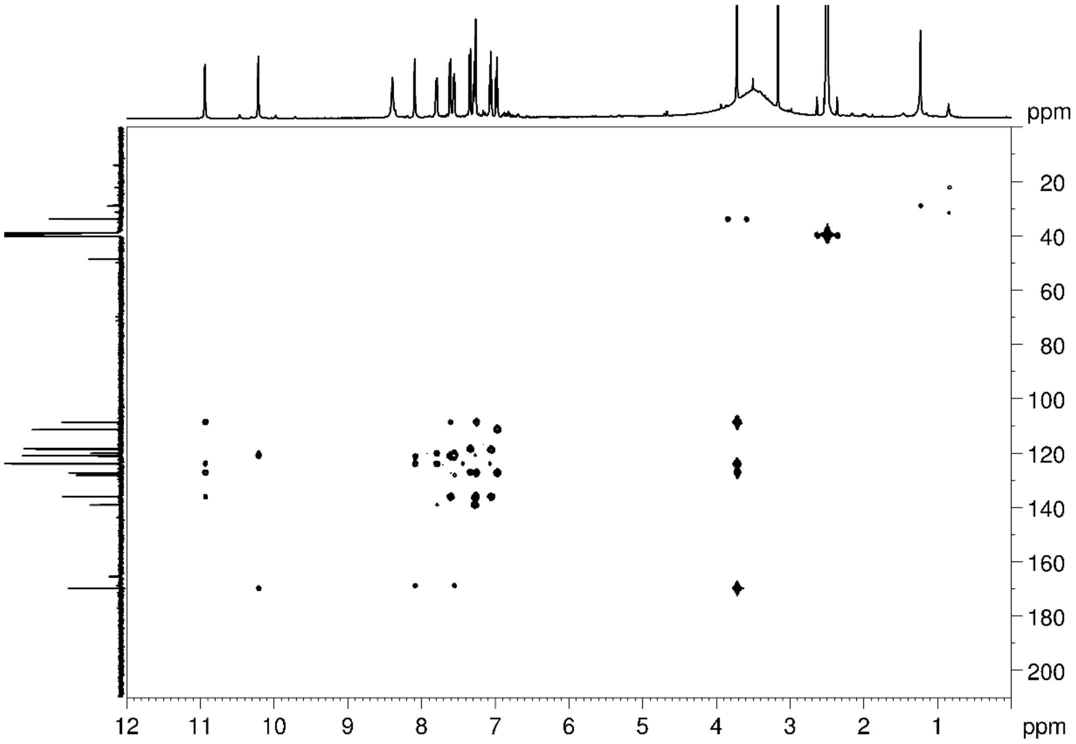

Figure S91. ^{1}H-^{13}C **HMBC NMR spectrum of lindolin K (3k) in DMSO-*d*$_6$.**

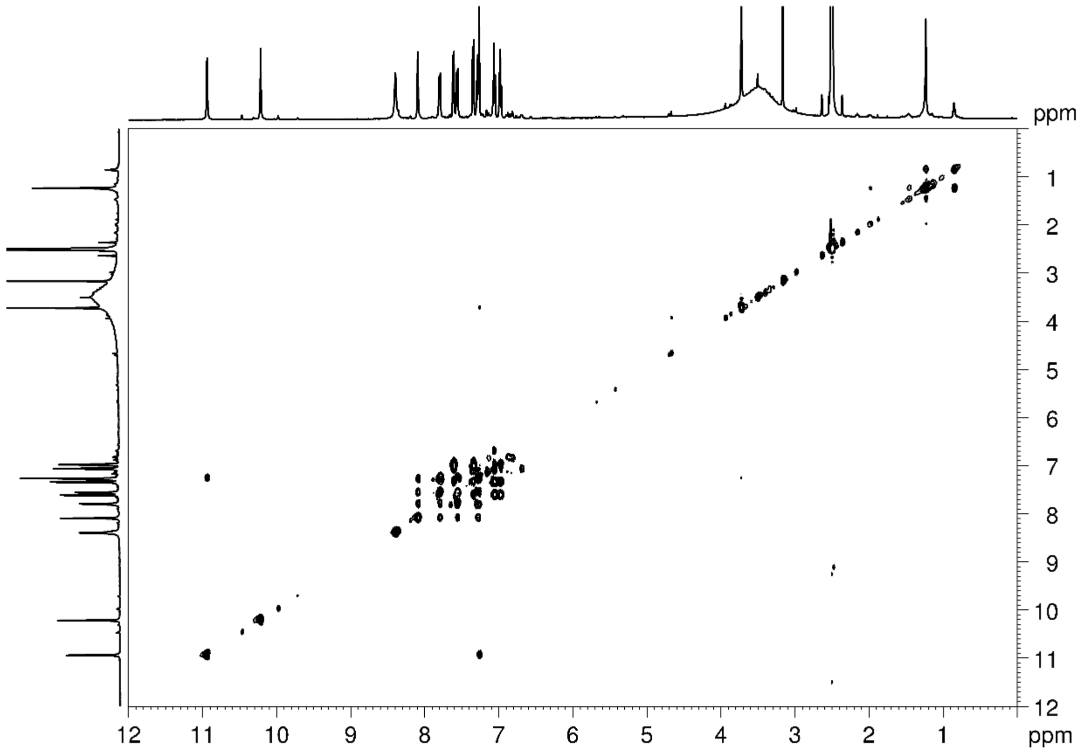

Figure S92. ^{1}H-^{1}H **TOCSY NMR spectrum of lindolin K (3k) in DMSO-*d*$_6$.**

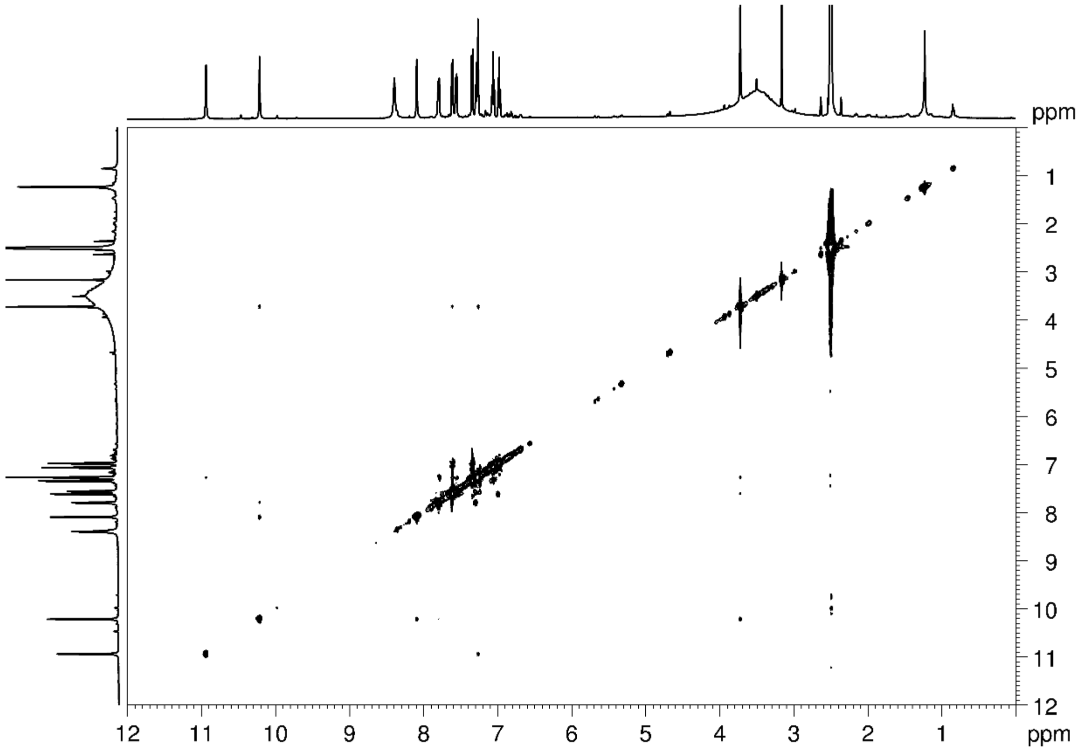

Figure S93. ^{1}H-^{1}H **ROESY NMR spectrum of lindolin K (3k) in DMSO-*d*$_6$.**

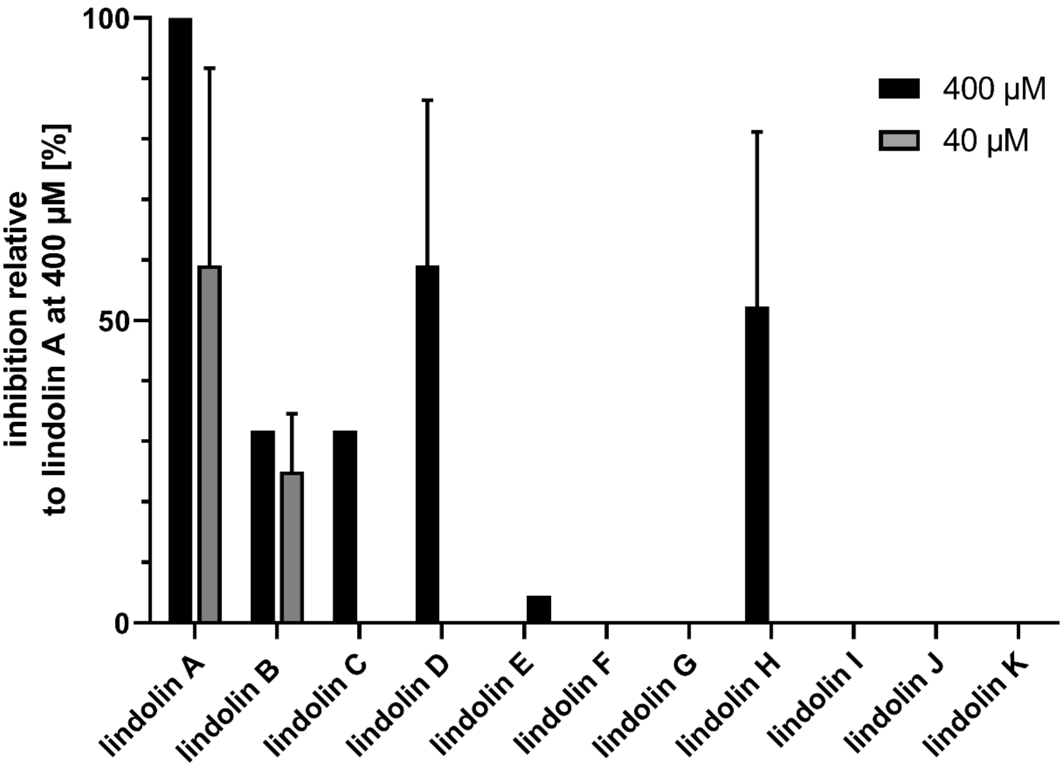

Figure S94. Anti-oomycete activity against *Phytophthora megasperma*.

The oomycete *Phytophthora megasperma* CBS 687.79 was cultivated on PDB agar plates in presence of **3a-3k** at 400 µM and 40 µM for eight days and colony diameter was determined as described.[1] Activity of lindolin A was defined as 100%. Each experiment was carried out in triplicate.

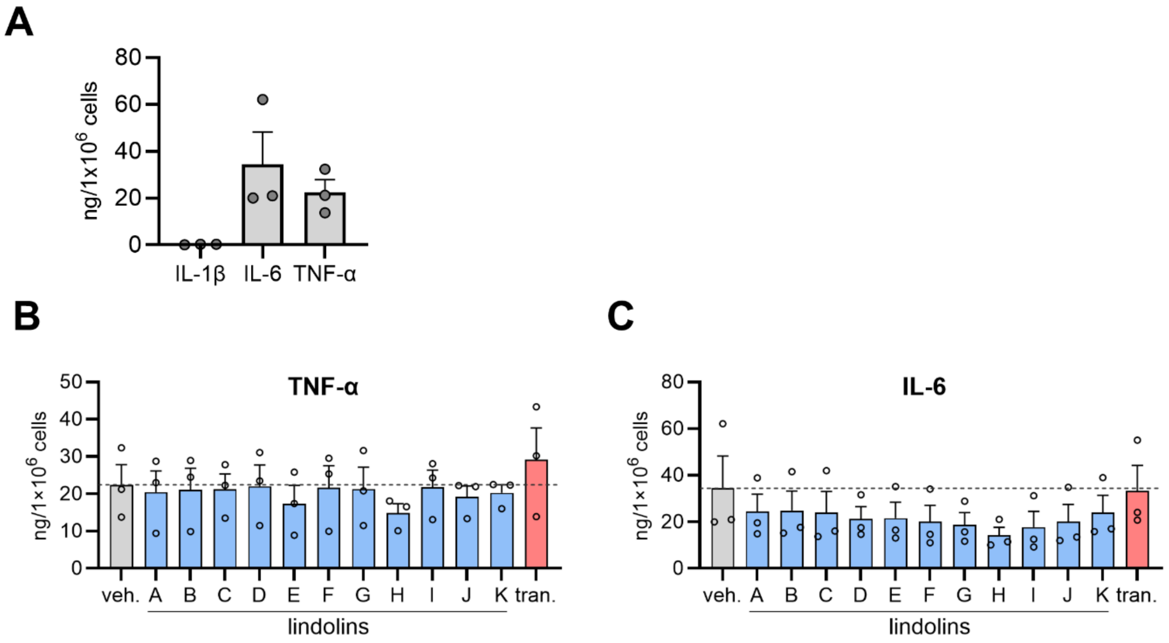

Figure S95. Impact of lindolins and tranilast on cytokine production in M1 macrophages.

Human M0 macrophages (1 x 10^6 cells) were pre-treated with 50 µM lindolins, 50 µM tranilast or 0.1% DMSO (as vehicle control) for 30 minutes prior to polarization with LPS/IFNγ for additional 24 h at 37 °C. Pro-inflammatory cytokines (IL-1β, TNF-α and IL-6) were measured by ELISA and shown as ng/1x10^6 cells in bar charts as single values with mean + SEM. Production of pro-inflammatory cytokines is shown in vehicle-treated macrophages (**A**), as well as, the impact of lindolins and tranilast on (**B**) TNF-α and (**C**) IL-6 formation.

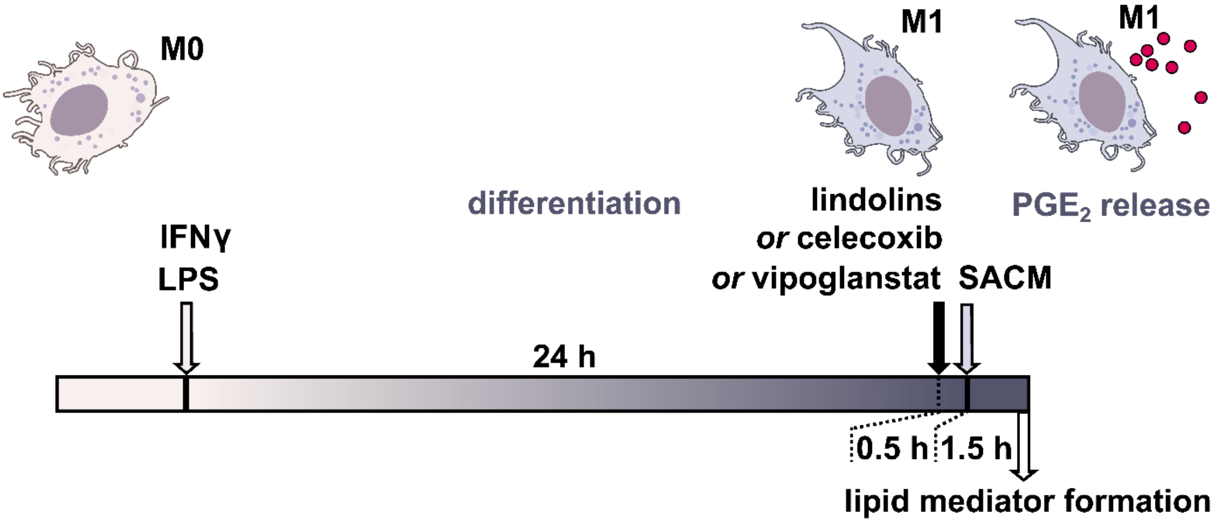

Figure S96. Visualization of short-term experiment with M1 macrophages to determine COX-2 and mPGES-1 enzyme inhibition.

M1 macrophages were pretreated with lindolin A (**3a**) (30 µM), celecoxib (3 µM), vipoglanstat (3 nM) or vehicle (DMSO, 0.1%) for 30 minutes prior to stimulation with 1% *Staphylococcus aureus*-conditioned medium (SACM) in PBS (pH 7.4; supplemented with 1 mM $CaCl_2$) for another 90 min at 37 °C. Produced lipid mediators were analyzed in cell supernatants by UHPLC-MS/MS (Figures 5A, 5B).

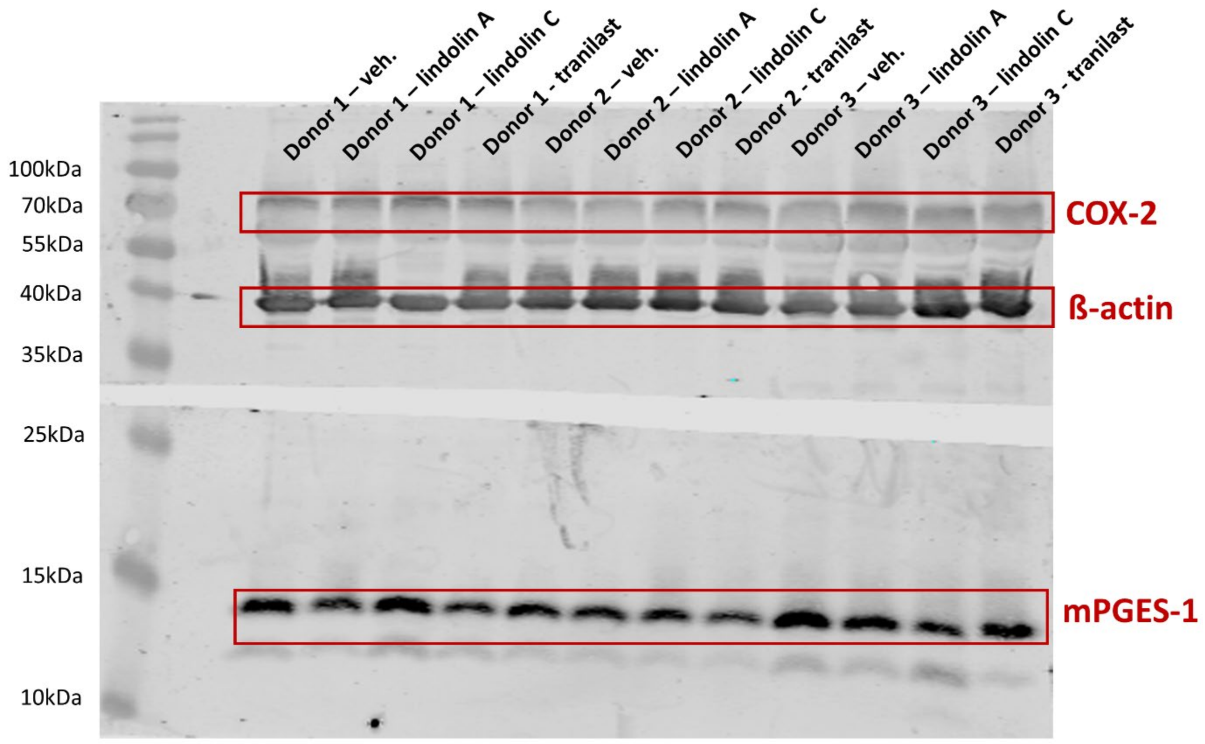

Figure S97. Western Blot to detect COX-2, β-actin and mPGES-1 in human primary M1 macrophages.

References

(1) Rassbach, J.; Hilsberg, N.; Haensch, V. G.; Dörner, S.; Gressler, J.; Sonnabend, R.; Semm, C.; Voigt, K.; Hertweck, C.; Gressler, M. Non-canonical two-step biosynthesis of anti-oomycete indole alkaloids in Kickxellales. *Fungal Biol. Biotechnol.* **2023**, *10* (1), 19. DOI: 10.1186/s40694-023-00166-x.

(2) Weigert, F. J.; Roberts, J. D. Carbon-13 Nuclear Magnetic Resonance Spectroscopy. Determination of Carbon-Fluorine Couplings. *J. Am. Chem. Soc.* **1970**, *93* (10), 2361–2369.

(3) Krauss, J.; Knorr, V.; Manhardt, V.; Scheffels, S.; Bracher, F. Synthesis of platensimycin analogues and their antibiotic potency. *Arch. Pharm. (Weinheim)* **2008**, *341* (6), 386-392. DOI: 10.1002/ardp.200700177.
